# Supplementary material for: Use of technology to prevent, detect, manage and control hypertension in sub-Saharan Africa: a systematic review
Source: BMJ Open. 2022 Apr 4;12(4):e058840. doi: 10.1136/bmjopen-2021-058840 (PMC8984054; doi:10.1136/bmjopen-2021-058840)
Supplement: Supplementary data [file bmjopen-2021-058840supp001.pdf]

Test S1. Search terms

MEDLINE/Embase:
exp Cardiovascular Diseases/ or exp Hypertension/ or ("cardiovascular disease*" or CVD* or "hypertensi*" or "high blood pressure" or "blood pressure" or stroke or "heart disease").tw. and exp "Africa South of the Sahara"/ or (Sahara* or sub-Sahara* or "sub-Sahara* Africa" or SSA or Cameroon or "Central African Republic" or Chad or Congo or "democratic republic of the Congo" or "equatorial Guinea" or Gabon or "Sao Tome and Principe" or Burundi or Djibouti or Eritrea or Ethiopia or Kenya or Rwanda or Somalia or "south Sudan" or Sudan or Tanzania or Uganda or Angola or Botswana or Eswatini or Lesotho or Malawi or Mozambique or Namibia or Zambia or Zimbabwe or Benin or "Burkina Faso" or "Cabo Verde" or "Cote d'Ivoire" or Gambia or Ghana or Guinea or "Guinea-Bissau" or Liberia or Mali or Mauritania or Niger or Nigeria or Senegal or "Sierra Leone" or Togo).tw. and exp Artificial Intelligence/ or exp Artificial Intelligence/ or exp Cell Phone/ or exp "Internet of Things"/ or exp Telemedicine/ or ("artificial intelligence" or ai or "machine learning" or "deep learning" or "neural network*" or "reinforcement learning" or "naive bayes" or "decision tree" or "random forest" or "support vector machine" or "k-nearest neighbour" or "linear discriminant analysis" or classification or clustering or "supervised learning" or telehealth or telemedicine or mhealth or "mobile phone" or smartphone or "internet of things" or "cell phone").tw. OR (exp Artificial Intelligence/ or ("artificial intelligence" or ai or "machine learning").tw.) and ("logistic regression" or "logit regression" or "multiple regression").tw. and exp diagnosis/ or exp therapeutics exp treatment outcome/ or (prevent* or screen* or detection or diagnos* or management or treatment or control).tw.
Web of Science:
TS = (prevent* or screen* or diagnos* or treatment or therapeutics or management or control) and S =("artificial intelligence" or ai or "machine learning" or "deep learning" or "neural network*" or "reinforcement learning" or "naive bayes" or "decision tree" or "random forest" or "support vector machine" or "k-nearest neighbour" or "linear discriminant analysis" or classification or clustering or "supervised learning" or telehealth or telemedicine or mhealth or "mobile phone" or smartphone or "internet of things" or "cell phone") OR (TS=("artificial intelligence" or ai or "machine learning") AND TS=("logistic regression" or "logit regression" or "multiple regression")) and TS = ("Africa south of Sahara" or "Sahara" or "sub-Sahara*" or "sub-Sahara* Africa" or SSA or Cameroon or “Central African Republic” or Chad or Congo or “democratic republic of the Congo” or “equatorial guinea” or Gabon or “Sao Tome and Principe” or Burundi or Djibouti or Eritrea or Ethiopia or Kenya or Rwanda or Somalia or “South Sudan” or Sudan or Tanzania or Uganda or Angola or Botswana or Eswatini or Lesotho or Malawi or Mozambique or Namibia or Zambia or Zimbabwe or Benin or “Burkina Faso” or “Cabo Verde” or “Cote d’Ivoire” or Gambia or Ghana or Guinea or “Guinea-Bissau” or Liberia or Mali or Mauritania or Niger or Nigeria or Senegal or “Sierra Leone” or Togo) and TS=(cardiovascular disease CVD* or hypertensi* or high blood pressure or blood pressure or stroke or heart disease)
Scopus:
((TITLE-ABS-KEY ("cardiovascular disease*" OR cvd* OR "hypertensi*" OR "high blood pressure" OR "blood pressure" OR stroke OR "heart disease"))) AND (TITLE-ABS-KEY ("artificial intelligence" OR ai OR "machine learning" OR "deep learning" OR "neural network*" OR "reinforcement learning" OR "naive bayes" OR "decision tree" OR "random forest" OR "support vector machine" OR "k-nearest neighbour" OR "linear discriminant analysis" OR classification OR clustering OR "supervised learning" OR telehealth OR telemedicine OR mhealth OR "mobile phone" OR smartphone OR "internet of things" OR "cell phone") OR (TITLE-ABS-KEY ("artificial intelligence" OR ai OR "machine learning") AND TITLE-ABS-KEY ("logistic regression" OR "logit regression" OR "multiple regression"))) AND (TITLE-ABS-KEY ("Africa south of sahara" OR "sahara" OR "sub-sahara*" OR "sub-sahara* africa" OR ssa OR cameroon OR "Central African Republic" OR chad OR congo OR "democratic republic of the Congo" OR "equatorial guinea" OR gabon OR "Sao Tome and Principe" OR burundi OR djibouti OR eritrea OR ethiopia OR kenya OR rwanda OR somalia OR "South Sudan" OR sudan OR tanzania OR uganda OR angola OR botswana OR eswatini OR lesotho OR malawi OR mozambique OR namibia OR zambia OR zimbabwe OR benin OR "Burkina Faso" OR "Cabo Verde" OR "Cote d'Ivoire" OR gambia OR ghana OR guinea OR "Guinea-Bissau" OR liberia OR mali OR mauritania OR niger OR nigeria OR senegal OR "Sierra Leone" OR togo)) AND (TITLE-ABS-KEY (prevent* OR screen* OR diagnos* OR treatment OR therapeutics OR management OR control))

Table S1. Articles excluded on reading full text.

Authors	Source	Year	Title	Reason:
T.; Njuguna Mercer, B.; Bloomfield, G. S.; Dick, J.; Finkelstein, E.; Kamano, J.; Mwangi, A.; Naanyu, V.; Pastakia, S. D.; Valente, T. W.; Vedanthan, R.; Akwanalo, C.	Trials	2019	Strengthening Referral Networks for Management of Hypertension Across the Health System (STRENGTHS) in western Kenya: a study protocol of a cluster randomized trial	No KETs
I.; Joloba Ddumba, S.; Kakande, B.	Transactions of the Royal Society of Tropical Medicine and Hygiene	2019	Motivations for participating in the use of mobile smart phone app focusing on monitoring of hypertension among older persons in Uganda	Insufficient detail
F. S.; Adusei Sarfo, N.; Ampofo, M.; Kpeme, F. K.; Ovbiagele, B.	Journal of the Neurological Sciences	2018	Pilot trial of a tele-rehab intervention to improve outcomes after stroke in Ghana: A feasibility and user satisfaction study	Not hypertension
F. S.; Adamu Sarfo, S.; Awuah, D.; Sarfo-Kantanka, O.; Ovbiagele, B.	Journal of the Neurological Sciences	2017	Potential role of tele-rehabilitation to address barriers to implementation of physical therapy among West African stroke survivors: A cross-sectional survey	Not hypertension
F.; Pudney Lorgat, E.; van Deventer, H.; Chitsaz, S.	Cardiovascular Journal of Africa	2012	Robotically controlled ablation for atrial fibrillation: the first real-world experience in Africa with the Hansen robotic system	Not hypertension
R.; Kamano Vedanthan, J. H.; Naanyu, V.; Delong, A. K.; Were, M. C.; Finkelstein, E. A.; Menya, D.; Akwanalo, C. O.; Bloomfield, G. S.; Binanay, C. A.; Velazquez, E. J.; Hogan, J. W.; Horowitz, C. R.; Inui, T. S.; Kimaiyo, S.; Fuster, V.	Trials [Electronic Resource]	2014	Optimizing linkage and retention to hypertension care in rural Kenya (LARK hypertension study): study protocol for a randomized controlled trial	No KETs
E.; Nyota Oduor, T.; Wachira, C.; Osebe, S.; Remy, S. L.; Walcott, A.	Journal	Year	Medication Management Companion (MMC) for a rural kenyan community	No KETs
J. T.; Tham Kamwesiga, K.; Guidetti, S.	Disability and Rehabilitation	2017	Experiences of using mobile phones in everyday life among persons with stroke and their families in Uganda - a qualitative study	Not hypertension
J. T.; Eriksson Kamwesiga, G. M.; Tham, K.; Fors, U.; Ndiwalana, A.; von Koch, L.; Guidetti, S.	Global Health	2018	A feasibility study of a mobile phone supported family-centred ADL intervention, F@ce TM, after stroke in Uganda	Not hypertension
F. K.; Adoubi Diby, A.; Gnaba, A.; Ouattara, P.; Ayegnon, K. G.; Boidy, K.; Azagoh-Kouadio, R.; Meneas, G.; Manga, D.; Coulibaly, A.; Sall, F.; Nguessan, E.; Ehui, E.; Yangni-Angate, K. H.	European Research in Telemedicine	2015	Tele-expertise in the interpretation of the electrocardiogram of a black African population in the Ivory Coast (sub-Saharan Africa)	Not hypertension
G. S.; Wang Bloomfield, T. Y.; Boulware, L. E.; Califf, R. M.; Hernandez, A. F.; Velazquez, E. J.; Peterson, E. D.; Li, J. S.	Global heart	2015	Implementation of management strategies for diabetes and hypertension: from local to global health in cardiovascular diseases	No KETs
T.; Dewyer Aliku, A.; Namuyonga, J.; Ssinabulya, I.; Kamarembo, J.; Okello, E.; Bua, B.; Asiimwe, A.; Odong, F.; Akech, R.; Beaton, A.; DeStigter, K.; Lwabi, P.; Sable, C.	Global Heart	2018	Telemedicine Support of Cardiac Care In Northern Uganda: Leveraging Hand-held Echocardiography and Task Shifting	Not hypertension
M.; Sarfo Nichols, F. S.; Singh, A.; Qanungo, S.; Treiber, F.; Ovbiagele, B.; Saulson, R.; Patel, S.; Jenkins, C.	American Journal of the Medical Sciences	2017	Assessing Mobile Health Capacity and Task Shifting Strategies to Improve Hypertension Among Ghanaian Stroke Survivors	Not hypertension
H. L.; Duhig Nathan, K.; Vousden, N.; Lawley, E.; Seed, P. T.; Sandall, J.; Bellad, M. B.; Brown, A. C.; Chappell, L. C.; Goudar, S. S.; Gidiri, M. F.; Shennan, A. H.; Cradle- Trial Collaboration Grp	Trials	2018	Evaluation of a novel device for the management of high blood pressure and shock in pregnancy in low-resource settings: study protocol for a stepped-wedge cluster-randomised controlled trial (CRADLE-3 trial)	No KETs
J. S.; Inyiama Igwe, H. C.; Alo, U. R.; Ajah, I. A.	International Journal of Scientific and Technology Research	2020	Interpretation of eeg recordings for the purpose of diagnosing stroke disease	Not hypertension
J. S.; Inyiama Igwe, H. C.; Alo, U. R.; Ajah, I. A.	Journal		Classification of human brain signal for diagnosis of stroke disease using artificial neural network	Not hypertension
O.; Olabode Olabode, B. T.	Journal of Computer Science	2012	Cerebrovascular accident attack classification using multilayer feed forward artificial neural network with back propagation error	Not hypertension
N.; Imberti Maurizi, J. F.; Faragli, A.; Targetti, M.; Baldini, K.; Sall, A.; Cisse, A.; Gigli Berzolari, F.; Borrelli, P.; Avvantaggiato, F.; Perlini, S.; Marchionni, N.; Cecchi, F.; Parigi, G. B.; Olivotto, I.	European Heart Journal	2016	Comparative analysis of a 4-lead portable smartphone-based versus standard 12-lead electrocardiograph for cardiovascular screening in low-income settings	Not hypertension
N.; Faragli Maurizi, A.; Imberti, J.; Briante, N.; Targetti, M.; Baldini, K.; Sall, A.; Cisse, A.; Berzolari, F. G.; Borrelli, P.; Avvantaggiato, F.; Perlini, S.; Marchionni, N.; Cecchi, F.; Parigi, G.; Olivotto, I.	International Journal of Cardiology	2017	Cardiovascular screening in low-income settings using a novel 4-lead smartphone-based electrocardiograph (D-Heart®)	Not hypertension
J. J.; Salehian Manolakos, O.; Kraeker, C.; Manolakos, L.; Hunter, C. J.	Canadian Journal of Cardiology	2015	Rheumatic heart disease screening in Windhoek Namibia using portable echocardiography: A pilot project	Not hypertension
G. F.; Shirk Evans, A.; Muturi, P.; Soliman, E. Z.	Global Heart	2017	Feasibility of Using Mobile ECG Recording Technology to Detect Atrial Fibrillation in Low-Resource Settings	Not hypertension

Table S2. Quality analysis

	RefID	31	32	40	28	41	34	39	42	43	47	33	48	38	44	37	49	29	36	35	46	30	45
	First author	Leon	Bobrow	Vedanthan	Kingue	Owolabi	Hacking	Sarfo	Sarfo	Nichols	Adler	Vedanthan	Vedanthan	Nelissen	Cremers	Mannik	Aw	Ola-Olorun	Kleczka	Haricharan	Odour	Joubert	Barsky
	Year	2015	2016	2019	2013	2019	2016	2018	2019	2019	2020	2015	2020	2018	2019	2018	2020	2014	2018	2017	2019	2014	2019
SCREENING QUESTIONS	S1. Are there clear research questions?	Yes	Yes	Yes	Yes	Yes	Yes	Yes	Yes	Yes	Yes	Yes	Yes	Yes	Yes	Yes	Yes	Yes	Yes	Yes	Yes	Yes	Yes
	S2. Do the collected data allow to address the research questions?	Yes	Yes	Yes	Yes	Yes	Yes	Yes	Yes	Yes	Yes	Yes	Yes	Yes	Yes	Yes	Yes	Yes	Yes	Yes	Yes	Yes	Yes
1. QUALITATIVE STUDIES	1.1. Is the qualitative approach appropriate to answer the research question?	Yes					Yes			Yes	Yes	Yes		Yes	Yes	Yes	Yes	Yes		Yes	Yes		Yes
	1.2. Are the qualitative data collection methods adequate to address the research question?	Yes					Yes			Yes	Yes	Yes		Yes	Yes	Yes	Yes	Yes		Yes	Yes		Yes
	1.3. Are the findings adequately derived from the data?	Yes					Yes			Yes	Yes	Yes		Yes	Yes	Can't tell	Yes	Yes		Yes	Yes		Can't tell
	1.4. Is the interpretation of results sufficiently substantiated by data?	Yes					Yes			Yes	Yes	Yes		Yes	Yes	No	Yes	Yes		Yes	Yes		Can't tell
	1.5. Is there coherence between qualitative data sources, collection, analysis and interpretation?	Yes					Yes			Yes	Yes	Yes		Yes	Yes	Yes	Yes	Yes		Yes	Yes		Can't tell
2. RANDOMIZED CONTROLLED TRIALS	2.1. Is randomization appropriately performed?		Yes	No	No	Yes	No	Yes	Yes														Yes
	2.2. Are the groups comparable at baseline?		Yes	No	No	Yes	Yes	Yes	Yes														Can't tell
	2.3. Are there complete outcome data?		Yes	No	Yes	Yes	No	Yes	Yes														Can't tell
	2.4. Are outcome assessors blinded to the intervention provided?		Yes	No	No	Yes	Can't tell	Yes	Yes														Can't tell
	2.5 Did the participants adhere to the assigned intervention?		Yes	Can't tell	Yes	Yes	Yes	Yes	Yes														Can't tell
3. NON-RANDOMIZED STUDIES	3.1. Are the participants representative of the target population?												Yes	Yes	Yes			Yes	Yes	No			
	3.2. Are measurements appropriate regarding both the outcome and intervention (or exposure)?												Yes	No	Yes			Yes	Yes	Yes			
	3.3. Are there complete outcome data?												No	No	No			Yes	Yes	No			
	3.4. Are the confounders accounted for in the design and analysis?												Yes	Yes	Can't tell			Can't tell	Yes	Yes			
	3.5. During the study period, is the intervention administered (or exposure occurred) as intended?												No	No	Yes			Yes	Yes	Yes			
4. QUANTITATIVE DESCRIPTIVE STUDIES	4.1. Is the sampling strategy relevant to address the research question?															Yes	Yes					Yes	
	4.2. Is the sample representative of the target population?															Yes	No					Yes	
	4.3. Are the measurements appropriate?															Yes	Yes					Yes	
	4.4. Is the risk of nonresponse bias low?														Yes	Yes					Yes		
	4.5. Is the statistical analysis appropriate to answer the research question?														Yes	Yes					Yes		
5. MIXED METHODS STUDIES	5.1. Is there an adequate rationale for using a mixed methods design to address the research question?					Yes							Yes	Yes	Yes	Yes	Yes		Yes			Yes	
	5.2. Are the different components of the study effectively integrated to answer the research question?					Yes							Yes	Yes	Yes	Yes	Yes		Yes			Yes	
	5.3. Are the outputs of the integration of qualitative and quantitative components adequately interpreted?					Yes							Yes	Yes	Can't tell	Yes	Yes		Yes			Can't tell	
	5.4. Are divergences and inconsistencies between quantitative and qualitative results adequately addressed?					Yes							Yes	Can't tell	Yes	Yes	Yes		Yes			Can't tell	
	5.5. Do the different components of the study adhere to the quality criteria of each tradition of the methods involved?					No							No	Yes	Yes	Yes	Yes		No			No	
	% criteria met	100	100	0	40	90	70	100	100	100	100	100	70	73.33333333	82.35294118	86.66666667	92.30769231	90	90	80	100	62.5	30

Table S3. Extracted baseline and endpoint systolic blood pressure readings for randomised controlled trials

**Author**	**Title**	**Year**	**Duration**	**Intervention type**	**Primary outcome**	**Secondary outcome**	**Monitoring tools**	**Groups**	**Number of participants (baseline)**	**Number of participants (endpoint)**	**Groups**
Bobrow et al.	Mobile Phone Text Messages to Support Treatment Adherence in Adults With High Blood Pressure (StAR): A Single-Blind, Randomized Trial	2016	12 months	SMS-Text Adherence suppoRt. Patients with high BP received SMS. Information only: Messages for motivation for medicine collection/taking or attending clinic, messages for education of hypertension. Interactive: Received same messages and were able to respond for changing an appointment, and changing the timing and language of the text-messages	Change in Systolic Blood Pressure at 12-months from baseline	Proportion of participants achieving mean SBP <140mmHg and mean DBP <90mmHg.<br>Health status (measured by self report questionnarire).<br>Proportion of scheduled clinic appointments attended.<br>Retention in clinical care. Satisfaction with clinic services and care.<br>Hospital admissions.<br>Self-reported adherence to medication.<br>Basic hypertension knowledge.<br>Number and type of medication changes made during trial.<br>Number of clinic visits.	validated oscillometric device. Recorded six sequential readings at three-minute intervals. The mean blood pressure was calculated by discarding the initial reading and calculating the mean from the five remaining readings.	Usual Care (UC)<br>Information Only (IO)<br>Interactive (I)			
								**UC**	457	396	**UC**
								**IO**	457	406	**IO**
								**I**	458	394	**I**
Vedanthan et al.	Community Health Workers Improve Linkage to Hypertension Care in Western Kenya	2019	15 months	Patients with high BP. Tailored behavioral communication + mHealth. Paper based: Community health worker ugave tailored behavioural and motivational engagement. Smartphone: CHW did same as PB, but had real-time decision support and data entry linked to electronic health record.<br>Cluster randomised	Co-primary outcomes:<br>Linkage to care<br>Change in SBP	N/A	automated Omron blood pressure machine, standard protocol (as described by World Health Organisation)	Usual Care (UC)<br>Paper Based (PB)<br>Smartphone (SP)			
								**UC**	422	355	**UC**
								**PB**	451	395	**PB**
								**SP**	465	356	**SP**
Owolabi et al.	Randomized Trial of an Intervention to Improve Blood Pressure Control in Stroke Survivors	2019	12 months	Patients with stroke-onset within one year. Intervention: chronic care model components of delivery system redesign (increased follow-up visits, pre-appointment phone texts), self-management support (patient report card, post-clinic follow-up phone texts, waiting room educational video), and clinical information systems (patient report card as part of medical chart, hospital registry).	Mean change in systolic blood pressure at 12 months	N/A	BP measurements were obtained and averaged from each subject with use of the Omron HEM-907XL26 according to a standardized protocol provided by the manufacturer about cuff size, cuff application, body position, and time intervals when taking a measurement.				
								**UC (high BP)**	**79**	**74**	**UC**
								**Intervention (high Bl**	**89**	**84**	**Interventio**
								**UC (all records)**	**199**	**188**	**UC**
								**Intervention (all reco**	**199**	**186**	**Interventio**

Table S4. PRISMA checklist

Section and Topic	Item #	Checklist item	Location where item is reported
**TITLE**			
Title	1	Identify the report as a systematic review.	1
**ABSTRACT**			
Abstract	2	See the PRISMA 2020 for Abstracts checklist.	2
**INTRODUCTION**			
Rationale	3	Describe the rationale for the review in the context of existing knowledge.	3
Objectives	4	Provide an explicit statement of the objective(s) or question(s) the review addresses.	3
**METHODS**			
Eligibility criteria	5	Specify the inclusion and exclusion criteria for the review and how studies were grouped for the syntheses.	3
Information sources	6	Specify all databases, registers, websites, organisations, reference lists and other sources searched or consulted to identify studies. Specify the date when each source was last searched or consulted.	3
Search strategy	7	Present the full search strategies for all databases, registers and websites, including any filters and limits used.	Supplementary
Selection process	8	Specify the methods used to decide whether a study met the inclusion criteria of the review, including how many reviewers screened each record and each report retrieved, whether they worked independently, and if applicable, details of automation tools used in the process.	3
Data collection process	9	Specify the methods used to collect data from reports, including how many reviewers collected data from each report, whether they worked independently, any processes for obtaining or confirming data from study investigators, and if applicable, details of automation tools used in the process.	4
Data items	10a	List and define all outcomes for which data were sought. Specify whether all results that were compatible with each outcome domain in each study were sought (e.g. for all measures, time points, analyses), and if not, the methods used to decide which results to collect.	4
	10b	List and define all other variables for which data were sought (e.g. participant and intervention characteristics, funding sources). Describe any assumptions made about any missing or unclear information.	4
Study risk of bias assessment	11	Specify the methods used to assess risk of bias in the included studies, including details of the tool(s) used, how many reviewers assessed each study and whether they worked independently, and if applicable, details of automation tools used in the process.	4
Effect measures	12	Specify for each outcome the effect measure(s) (e.g. risk ratio, mean difference) used in the synthesis or presentation of results.	4
Synthesis methods	13a	Describe the processes used to decide which studies were eligible for each synthesis (e.g. tabulating the study intervention characteristics and comparing against the planned groups for each synthesis (item #5)).	4
	13b	Describe any methods required to prepare the data for presentation or synthesis, such as handling of missing summary statistics, or data conversions.	4
	13c	Describe any methods used to tabulate or visually display results of individual studies and syntheses.	4
	13d	Describe any methods used to synthesize results and provide a rationale for the choice(s). If meta-analysis was performed, describe the model(s), method(s) to identify the presence and extent of statistical heterogeneity, and software package(s) used.	4

Section and Topic	Item #	Checklist item	Location where item is reported
	13e	Describe any methods used to explore possible causes of heterogeneity among study results (e.g. subgroup analysis, meta-regression).	N/A
	13f	Describe any sensitivity analyses conducted to assess robustness of the synthesized results.	N/A
Reporting bias assessment	14	Describe any methods used to assess risk of bias due to missing results in a synthesis (arising from reporting biases).	N/A
Certainty assessment	15	Describe any methods used to assess certainty (or confidence) in the body of evidence for an outcome.	N/A
**RESULTS**			
Study selection	16a	Describe the results of the search and selection process, from the number of records identified in the search to the number of studies included in the review, ideally using a flow diagram.	4
	16b	Cite studies that might appear to meet the inclusion criteria, but which were excluded, and explain why they were excluded.	Supplementary
Study characteristics	17	Cite each included study and present its characteristics.	Table 1
Risk of bias in studies	18	Present assessments of risk of bias for each included study.	7 and Supplementary
Results of individual studies	19	For all outcomes, present, for each study: (a) summary statistics for each group (where appropriate) and (b) an effect estimate and its precision (e.g. confidence/credible interval), ideally using structured tables or plots.	8
Results of syntheses	20a	For each synthesis, briefly summarise the characteristics and risk of bias among contributing studies.	8
	20b	Present results of all statistical syntheses conducted. If meta-analysis was done, present for each the summary estimate and its precision (e.g. confidence/credible interval) and measures of statistical heterogeneity. If comparing groups, describe the direction of the effect.	8
	20c	Present results of all investigations of possible causes of heterogeneity among study results.	N/A
	20d	Present results of all sensitivity analyses conducted to assess the robustness of the synthesized results.	N/A
Reporting biases	21	Present assessments of risk of bias due to missing results (arising from reporting biases) for each synthesis assessed.	N/A
Certainty of evidence	22	Present assessments of certainty (or confidence) in the body of evidence for each outcome assessed.	N/A
**DISCUSSION**			
Discussion	23a	Provide a general interpretation of the results in the context of other evidence.	8
	23b	Discuss any limitations of the evidence included in the review.	8
	23c	Discuss any limitations of the review processes used.	9
	23d	Discuss implications of the results for practice, policy, and future research.	9
**OTHER INFORMATION**			

Section and Topic	Item #	Checklist item	Location where item is reported
Registration and protocol	24a	Provide registration information for the review, including register name and registration number, or state that the review was not registered.	3
	24b	Indicate where the review protocol can be accessed, or state that a protocol was not prepared.	3
	24c	Describe and explain any amendments to information provided at registration or in the protocol.	3
Support	25	Describe sources of financial or non-financial support for the review, and the role of the funders or sponsors in the review.	1, 4, 9
Competing interests	26	Declare any competing interests of review authors.	9
Availability of data, code and other materials	27	Report which of the following are publicly available and where they can be found: template data collection forms; data extracted from included studies; data used for all analyses; analytic code; any other materials used in the review.	N/A
