# Supplementary material for: Use of technology to prevent, detect, manage and control hypertension in sub-Saharan Africa: a systematic review
Source: BMJ Open. 2022 Apr 4;12(4):e058840. doi: 10.1136/bmjopen-2021-058840 (PMC8984054; doi:10.1136/bmjopen-2021-058840)
Supplement: Author's manuscript [file bmjopen-2021-058840.draft_revisions.pdf]

BMJ Open

BMJ Open is committed to open peer review. As part of this commitment we make the peer review history of every article we publish publicly available.

When an article is published we post the peer reviewers' comments and the authors' responses online. We also post the versions of the paper that were used during peer review. These are the versions that the peer review comments apply to.

The versions of the paper that follow are the versions that were submitted during the peer review process. They are not the versions of record or the final published versions. They should not be cited or distributed as the published version of this manuscript.

BMJ Open is an open access journal and the full, final, typeset and author-corrected version of record of the manuscript is available on our site with no access controls, subscription charges or pay-per-view fees (http://bmjopen.bmj.com).

If you have any questions on BMJ Open's open peer review process please email info.bmjopen@bmj.com

BMJ Open

Use of technology to prevent, detect, manage, and control hypertension in sub-Saharan Africa: a systematic review

Journal:	*BMJ Open*
Manuscript ID	bmjopen-2021-058840
Article Type:	Original research
Date Submitted by the Author:	29-Oct-2021
Complete List of Authors:	Stokes, Katherine; University of Warwick,<br>Oronti, Busola; University of Warwick<br>Cappuccio, Francesco Paolo; University of Warwick, Warwick Medical School, Division of Health Sciences<br>Pecchia, Leandro ; University of Warwick,
Keywords:	Hypertension < CARDIOLOGY, Telemedicine < BIOTECHNOLOGY & BIOINFORMATICS, Information technology < BIOTECHNOLOGY & BIOINFORMATICS

SCHOLARONE™
Manuscripts

I, the Submitting Author has the right to grant and does grant on behalf of all authors of the Work (as defined in the below author licence), an exclusive licence and/or a non-exclusive licence for contributions from authors who are: i) UK Crown employees; ii) where BMJ has agreed a CC-BY licence shall apply, and/or iii) in accordance with the terms applicable for US Federal Government officers or employees acting as part of their official duties; on a worldwide, perpetual, irrevocable, royalty-free basis to BMJ Publishing Group Ltd ("BMJ") its licensees and where the relevant Journal is co-owned by BMJ to the co-owners of the Journal, to publish the Work in this journal and any other BMJ products and to exploit all rights, as set out in our licence.

The Submitting Author accepts and understands that any supply made under these terms is made by BMJ to the Submitting Author unless you are acting as an employee on behalf of your employer or a postgraduate student of an affiliated institution which is paying any applicable article publishing charge ("APC") for Open Access articles. Where the Submitting Author wishes to make the Work available on an Open Access basis (and intends to pay the relevant APC), the terms of reuse of such Open Access shall be governed by a Creative Commons licence – details of these licences and which Creative Commons licence will apply to this Work are set out in our licence referred to above.

Other than as permitted in any relevant BMJ Author's Self Archiving Policies, I confirm this Work has not been accepted for publication elsewhere, is not being considered for publication elsewhere and does not duplicate material already published. I confirm all authors consent to publication of this Work and authorise the granting of this licence.

For peer review only

Title	**Use of technology to prevent, detect, manage, and control hypertension in sub-Saharan Africa: a systematic review**
Authors' names	Katy Stokes[1*], Busola Oronti[1], Francesco P Cappuccio[2], Leandro Pecchia[1]
Address for each author	1. School of Engineering, University of Warwick, Coventry, UK<br>2. Medical School, University of Warwick, Coventry, UK
Authors' names and positions	Katy Stokes<br>PhD student<br>Busola Oronti<br>PhD student<br>Francesco P Cappuccio<br>Professor<br>Leandro Pecchia<br>Professor
Corresponding author	*Correspondence to: Katy.Stokes@warwick.ac.uk

For peer review only

Abstract

Objective To identify and assess the use of technologies, including mobile health technology, internet of things devices and artificial intelligence used in hypertension healthcare in sub-Saharan Africa (SSA).

Design Systematic review.

Data sources Medline, Embase, Scopus and Web of Science.

Eligibility criteria Studies addressing outcomes related to the use of technologies for hypertension healthcare (all points in the healthcare cascade) in SSA.

Methods Databases were searched from inception to Aug 2, 2021. Screening, data extraction and risk of bias assessment were done in duplicate. Data was extracted on study design, setting, technology(s) employed and outcomes. Blood pressure (BP) reduction due to intervention was extracted from a subset of randomised controlled trials. Methodological quality was assessed using the Mixed Methods Appraisal Tool (MMAT).

Results 1717 hits were retrieved, 1206 de-duplicated studies were screened, and 67 full texts were assessed for eligibility. 22 studies were included, all reported on clinical investigations. Two studies were observational, and 20 evaluated technology-based interventions. Outcomes included BP reduction/control, treatment adherence, retention in care, awareness/knowledge of hypertension and completeness of medical records. All studies utilised mobile technology, three linked with IoT devices. SMS was the most popular method of targeting patients (n=6). Moderate BP reduction was achieved in three randomised controlled trials. Patients and healthcare providers reported positive perceptions towards the technologies. No studies using AI were identified.

Conclusions There are a range of successful applications of key enabling technologies in SSA, including BP reduction, increased health knowledge and treatment adherence following targeted mobile technology interventions. There is evidence to support use of mobile technology for hypertension management in SSA. However, current application of technologies is highly heterogenous and key barriers exist, limiting efficacy and uptake in SSA. More research is needed, addressing objective measures such as BP reduction in robust randomised studies.

Registration PROSPERO CRD42020223043.

Funding KS is funded by the Medical Research Council Doctoral Training Partnership [grant number MR/N014294/1].

For peer review only

Strengths and limitations of this study

- This is the first systematic review for use of technologies for hypertension healthcare in sub-Saharan Africa, providing a comprehensive review of the state of the art.
- Heterogeneity of included studies was too high for meta-analysis; therefore, results are reported narratively.
- Grey literature was not searched.

Article Summary

What is already known on this topic

- Evidence for use of mHealth in SSA is growing but still limited.
- We identified three general/scoping reviews concerning use of mobile health in SSA for addressing non-communicable disease diagnosis or treatment and a single non-systematic review of the use of telehealth in the hypertension control cascade in SSA.
- We found no published systematic reviews or meta-analyses on use of technologies for hypertension healthcare in SSA.

What this study adds

- Application of technologies for hypertension healthcare is highly heterogeneous and key barriers limit efficacy and uptake in SSA.

For peer review only - http://bmjopen.bmj.com/site/about/guidelines.xhtml

- Our study suggests that mobile technology interventions can be successful in reducing BP, increasing health knowledge, and improving treatment adherence in SSA.
- More high-quality primary research is required to improve our understanding of the effects of different types of interventions on different populations in SSA.

For peer review only

Introduction

Cardiovascular disease (CVD) remains the most common cause of death due to non-communicable disease worldwide, with 78% of deaths occurring in low- and middle-income countries (LMICs).[1 2] Hypertension (high blood pressure (BP)) is considered by the World Health Organisation (WHO) to be the leading risk factor for developing CVD[1] and by the Pan-African Society of Cardiology (PASCAR) as the highest priority area for reducing heart disease and stroke in Africa.[3] In sub-Saharan Africa (SSA), the prevalence of hypertension is high, especially in younger subjects, estimated at 46% of the adult population, in contrast with 35% in high-income countries.[3] Reasons proposed for this include urbanization, increase in life expectancy and lifestyle factors such as poor diet, physical inactivity and smoking.[4] A meta-analysis performed by Atakite et al. in 2011 reports that of those with hypertension in SSA, only 27% were aware of their condition, 18% were receiving treatment and 7% had controlled blood pressure.[5]

Low numbers of trained healthcare providers combined with a lack of evidence-based guidance and a high-cost in accessing healthcare services for patients in SSA are major challenges.[6] Cost-effective technologies will likely play a critical role to overcome such barriers, through decision support tools;[7 8] dissemination of health information including education and treatment reminders[9 10] and collection and storage of medical data.[11 12] Indeed, the value of information and communication technologies (ICT) to health services has been recognised by the WHO for over ten years.[13] eHealth is defined by the WHO as "the cost-effective and secure use of ICT in support of health and health-related fields, including health-care services, health surveillance, health literacy, and health education, knowledge and research".[14] In this way, eHealth can be delivered through several Key Enabling Technologies (KETs): mobile phone technology, Artificial Intelligence (AI) and the Internet of Things (IoT). Mobile phone use is high in SSA, 45% of the population subscribe to mobile services and this use is projected to increase.[15] Research interests into use of mobile phones for healthcare purposes in SSA concern either infectious disease or maternal and child health,[16-18] but attention to NCD is growing.[19 20] AI has many possible definitions, in essence describing a motivation to replicate and automate human cognitive functions, having a myriad of healthcare applications[21] which have been exploited in high-income countries. Although research in LMICs is relatively limited,[22] drivers such as high disease burden, few qualified health care workers and increasing phone and internet connection may drive a rapid advance in AI for healthcare in LMICs.[23] Wahl et al. describe uses of AI in resource-poor settings, including expert systems assisting or compensating for a lack of personnel, health monitoring using natural language processing and signal processing for diagnostics.[22]

Successful application of the afore mentioned technologies for tackling hypertension relies on a strong evidence base in design and implementation. In this way, this work seeks to systematically review the literature regarding the application of mobile phone technologies, AI and the IoT as KETs for healthcare provision for hypertension in SSA. The primary objective is to determine how and which KETs have been used, secondary concerns include study design, setting, quality and findings of outcomes relating to hypertension.

Methods

Search strategy and selection criteria

The systematic review of KETs for healthcare provision for hypertension in SSA followed Preferred Reporting Items for Systematic Reviews and Meta-Analyses (PRISMA) 2020 guidelines.[24] We searched Embase, MEDLINE, Web of Science and Scopus electronic databases. The search was run from database inception to Nov 23rd, 2020 and updated Aug 2nd, 2021.

Search terms covered hypertension (e.g., "hypertension", "high blood pressure"), Artificial Intelligence (e.g., "AI", "machine learning"), mobile phones (e.g., "mobile phon*", "mobile"), internet of things (e.g., "internet of things", "iot"), point of healthcare cascade (e.g., "prevention", "screening") and countries of SSA (full strings in appendix).

Studies seeking to assess the application of KETs in SSA, for any point in the healthcare cascade for hypertension, were considered for inclusion. There were no restrictions set on study methodology in terms of participant recruitment, age, or comorbidity. Studies were required to be conducted using populations from SSA countries, or from a pool of countries including at least one SSA country. For inclusion, studies must have provided an evaluation of the use of KETs for any aspect of healthcare for hypertension or used AI models for predicting or detecting significant events. Studies which focused only on prevalence or risk factors, i.e., used statistical methods but did not develop AI based predictive models, were considered out of the scope of this review. The study protocol was registered with PROSPERO: International Prospective Register of Systematic Reviews and is found at: http://www.crd.york.ac.uk/prospero – registration number CRD42020223043. Ethical approval was not required.

For peer review only - http://bmjopen.bmj.com/site/about/guidelines.xhtml

Data analysis

Screening was completed independently by two authors (KS and BO). A reference search was conducted on any relevant review articles retrieved. For included studies, data were abstracted to a shared Microsoft Excel document, covering study design, study setting and population (age, demographics, comorbidity), details of KET used, study outcomes, controls/comparators (where applicable), target user (where applicable) and indications of acceptability to user (if provided). For randomised controlled trials, we sought to extract mean baseline and endpoint blood pressure measurements (in mm Hg), with standard deviation, for the intervention and control groups. If standard deviation was not reported, it was calculated using the confidence interval, as described in the Cochrane Handbook for Systematic Reviews of Interventions.[25] In the event that participants were lost to follow-up, the final number of participants that completed the study protocol was extracted. As heterogeneity among studies was high (no two studies evaluated the same intervention), we used a random-effects model to establish the effect of KET based interventions on systolic blood pressure reduction. We did the analysis in Open Meta-Analyst,[26] an open-source, cross-platform software for meta-analysis.

Methodological quality was assessed independently by two review authors (KS and BO) using the 2018 Mixed Methods Appraisal Tool (MMAT)[27] for assessing the quality of either quantitative, qualitative, or mixed methods studies. Criteria were graded as 'unmet', 'met' or 'can't tell'. For mixed methods studies, provided most criteria were met (three or more out of five) for each component, the components were considered to have adhered to their respective quality criteria (criterion 5.5).

Role of funding source

The funder of the study had no role in study design, data collection, data analysis, data interpretation, or writing of the report. Patients and the public were not involved in this research.

Results

Searching MEDLINE, Scopus, Embase and Web of Science yielded 1717 titles. After duplicate removal, 1206 titles/abstracts were screened, with 1139 excluded. Of the remaining 67 full-texts, 18 studies were found to meet the inclusion criteria (**figure 1**). A further four studies were identified through a linear search of the bibliographies of relevant reviews identified during the initial screening. **Table 1** contains a summary of the characteristics of the included studies.

Figure 1. Study selection

Table 1. Characteristics of included studies

Study design, participants, location, and aims were highly heterogenous (**table 1**). The distribution of studies by country is shown in **figure 2**. All included articles reported on clinical investigations.[28-49] Two were observational studies [30 46] and 20 concerned evaluation of KET based interventions.[28 29 31-45 47-49] Both observational studies utilised mixed methods to explore either current use of technology for hypertension management or hypertension prevalence, understanding and awareness. Several of the interventional studies fell within the same larger study, granting fourteen unique experimental studies. Separate articles within these studies reported on different aspects such as impact of intervention, feasibility, and perceptions. Eight studies were randomised controlled trials.[28 31 32 34 39-43 45 47] Quantitative primary outcomes were BP reduction and BP control. Other outcomes included treatment adherence, retention in care, awareness/knowledge of hypertension and completeness of medical records. Length of exposure was highly heterogeneous, ranging from 17 weeks to two years.

Quality was highest for qualitative and quantitative descriptive methods and relatively low for randomised or non-randomised studies (appendix). 11 of 13 studies comprising qualitative components satisfied all criteria,[29 31 33-35 38 43 46 47 49] whereas insufficient reporting of results in two studies meant it was not possible to determine if findings had been adequately derived or substantiated from the data.[37 45] In terms of randomised controlled trials, four met all quality criteria[32 39 41 42], three did not use appropriate randomisation methods[28 34 40] and three did not report complete outcome data.[34 40 45] Notably, Barsky et al. did not report sufficient information to judge four out of five criteria[45] and Vedanthan et al. failed to meet any criteria.[40] Lack of complete outcome data was also an issue in four out of six non-randomised studies.[35 38 44 48] Three of four quantitative descriptive studies met all quality criteria[30 37 42] with one study subject to voluntary selection bias, which was discussed by the authors.[49]

All studies employed adult populations, with varying age requirements (table 1). Five studies used subjects or data collected in a predominantly urban setting,[31 34-36 38], six in rural locations,[28 33 37 40 45 46] two in both[30 41] and one study did not provide specific location or population demographic details.[29] The majority of the

experimental research recruited subjects with elevated BP[40] or confirmed hypertension/prescribed anti-hypertensives,[28 29 31 33 34 36 38 39 47] with the aim of improving hypertension control, treatment adherence or health knowledge, otherwise, the aim of the study was to test a health tool for identifying hypertension or general CVD risk factors within a certain population.[37] In terms of comorbidity or other conditions, one study focused on patients having recently suffered stroke (with or without hypertension),[41] one focused on diabetic patients,[28] one concerned HIV positive hypertensive subjects[46] and one employed a convenience sample of a Deaf community.[35] In three cases, participants were required to have access to a mobile phone for inclusion in the study.[31 41 47]

Figure 2: Distribution of studies across sub-Saharan Africa.
Countries are coloured based on the number of studies conducted (darker indicates more studies) and annotated with frequency.

Table 2 describes the technologies used and **figure 3** displays the frequency of use of each aspect of mobile technology. All studies utilised mobile phone technology, including SMS, phone calls and mobile applications (apps), either alone or in combination. Odour et al. evaluated the general use and perceptions of medical practitioners and patients towards technology, in particular mobile technology, in dealing with hypertension and HIV. IoT devices employed were automatic BP monitors and their use was also facilitated by mobile phones.

Table 2. Summary of KETs utilised in study pool

Figure 3. Frequency of use of different aspects of mobile technology among study pool.

SMS messages were mostly targeted to patients, for health knowledge improvement,[34 35] motivation/improved treatment adherence[29 39 41] or both.[31] Content included reminders for taking medication or attending clinics/appointments, educational information covering general healthy lifestyle suggestions (e.g., eating habits, exercise), or hypertension information (e.g., symptoms, further health consequences, medication information). SMS were also used in combination with other elements in broader interventions to facilitate decision support for healthcare providers (e.g., through direct feedback of risk stratification)[28 37] or to provide an interactive connection between patients and pharmacists[29] or specialist telehealth connection (e.g., direct feedback from cardiologist to pharmacists).[28 44]

Apps were targeted to health care providers, most frequently community health workers (CHWs). Three studies utilised apps designed to mediate BP reading collection and dissemination of results for risk assessment and follow-up.[42 45 49] The DREAM-GLOBAL app received BP readings from an automatic monitor via Bluetooth (UA-767 Plus BT), relayed the readings to a remote central server, which then calculated the average reading and transmitted the patients results to their primary health care provider by fax, and to the patient themselves by SMS. [45] For high BP readings, the patient was notified to seek advice with their health care provider. Similarly, in the Phone-based Intervention under Nurse Guidance after Stroke (PINGS) study, the same Bluetooth BP monitor was linked with an app for monitoring and reporting measurements as well as medication intake. Participants monitored their own BP, following training from a study nurse. Levels of medication intake were monitored, and tailored motivational SMS were delivered to participants based on these results.[39] The AFYACHAT mobile app functioned in a similar way, BP readings were entered by the CHW, along with other patient data, the app then provided an algorithmic risk stratification (based on WHO's Prevention of CVD: Pocket Guidelines for Assessment and Management of Cardiovascular Risk, 2007) via SMS.[37 49] The Decision-Support and Integrated Record-Keeping (DESIRE) tool, an app designed for nurses to use on tablets, also provided clinical decision support, here through the AMPATH hypertension management algorithm, which is based on WHO clinical algorithms.[33] The DESIRE tool included functions for data entry and validation, decision support, alerts, and reminders, and viewing historical data on. Other authors discussed the feasibility of mobile technology facilitated screening for hypertension. Joubert et al. collected survey data using a tablet computer to collect and relay patient information to a central database.[30] Another focus of research was digitisation and storage of patient data from previous paper-based systems. Kleczkaa et al. described use of rubber stamp templates containing checklists of clinical practice guidelines; smartphone cameras were used to take images of these templates which were then manually synched to a cloud-based database, with plans for further automation.[36] A cloud-based health records system was also utilised as part of the ComHIP hypertension improvement project, which facilitated delivery of SMS and aimed to allow all levels of health providers access to patients' records.

Concerning outcomes relating either to reduction of BP or improved BP control, the majority of experimental studies reported that their interventions resulted in improvements.[28 32 38 40 42] However, analysis by Nelissen et al.[38] found that the mHealth app element of their intervention was not associated with the observed BP improvements, based on duration of patient activity measured by the app.[38] Four studies detected no difference[41] or statistically insignificant changes[32 39 40] between control and intervention groups. Vedanthan et al. observed

For peer review only

significant reduction in both systolic and diastolic BP regardless of whether their tablet-based decision support tool was used by nurses or clinical officers but did not have a control group. Heterogeneity in both outcomes investigated and reported prevented quantitative comparison. Results from three randomised controlled trials which reported baseline and endpoint values for systolic BP are presented in figure 4. It should be emphasised that these trials differed greatly in their intervention plan, study design and location (see table 1), and therefore, it was not appropriate to report an overall effect. Bobrow et al. and Owolabi et al. met all quality criteria, however Vedanthan et al.[48] failed to meet any, with authors describing difficulties in data collection and high levels of missing data. Some authors stated it had not been feasible to power studies to detect significant BP reduction, for example, the three month interim results of the PINGS trial did not find significant BP reduction due to the intervention until after nine months, when the proportion of participants with controlled BP became significantly higher in the intervention arm (46.7% vs 40%).[39 42] In some cases, authors noted that effects varied between subjects based on initial hypertension severity. An instance of this is Kingue et al., where greater overall improvements (BP improved or BP at target) in participants with stage 3 hypertension was observed.[28] Owolabi et al. also observed a significant reduction in blood pressure for a subset of subjects with baseline BP>140.90mmHg, but not an overall significant reduction for all participants.[41]

Figure 4. Change in mean systolic blood pressure (mmHg) between control and intervention groups.

In terms of health knowledge improvement, in one study CVD nursers reported that their own hypertension awareness and knowledge increased, as well as that of the community, due to the ComHIP project.[47] Hacking et al. found no statistical change in overall health knowledge, however medication adherence was significantly higher due to intervention and self-reported behaviour change improvements.[34] Haricharan et al. reported significant improvements in overall knowledge of healthy living and hypertension following exposure to informative SMS messaging.[35] PINGS resulted in significant medication adherence improvements,[39] and TELEMED-CAM saw significantly higher medical visit adherence in their intervention group.[28] Ola-Olorun et al. reported positive perceptions towards a SMS based medicine information exchange between patients and pharmacists, patients requested information on adverse medication effects.[29] Clinical documentation improved for all non-communicable diseases investigated by Kleczka et al., with a 21% improvement in hypertension documentation scoring.[36]

When perceptions towards the proposed technology were gathered, interventions were well received by patients[29 31 34 47] and health professionals.[29 37 44 47] Reported concerns included access to/stability of internet connection,[47] power availability,[37 44 47] cost,[44 47] increased workload,[38] understanding of SMS wording,[33] unfamiliarity with mobile technology or technology not being 'user-friendly',[34 44] and duplication in digital patient records.[49] Focus groups and interviews conducted by Adler et al. indicated that health providers and policy makers identified major challenges in the use of a cloud-based health records system, which would require heavy reliance on outside resources.[47]

Discussion

Our systematic review of the literature found broad and diverse applications of KETs for tackling hypertension in SSA. The findings indicated there is still relatively limited published research, particularly of controlled trials. All studies leveraged mobile phones for purposes of screening for hypertension, improving patient knowledge/treatment adherence or aiding non physician healthcare workers in providing hypertension care. Overall, studies reported success in these outcomes, with overwhelmingly positive responses from participants towards the use of KETs. However, very few studies were able to demonstrate statistically significant improvements over standard care, when evaluating objective measures such as BP reduction. This may indicate persistent difficulties in designing and implementing technology-based healthcare solutions in low-resource settings. Such difficulties were also evident from the quality analysis, for example a frequent issue identified in the quality analysis for both randomised and nonrandomised trials was a lack of complete outcome data, with authors describing difficulties with missing information and loss to follow-up as high as 50% in one study[35] and 58% in another,[48] an important consideration for future studies.

No publications using AI were identified in this study. Owoyemi et al.[50] suggested that reasons for this may include: limited available data, a lack of policy and legal framework, associated cost of uptake and inadequate infrastructure. However, our search yielded eight studies using logistic regression analysis to evaluate hypertension risk factors. Seven studies concerned hypertension prevalence and risk factors,[51-57] one study assessed determinants of follow-up BP control in stroke survivors,[58] and one sought factors which affected hypertension admission outcomes (died, discharged, or referred).[59] There was strong overlap in the identified risk factors (appendix). It will be interesting for future research when expanding on this work, to establish evidence-based, predictive AI modelling either for screening or diagnostic tools, or to identify and target the most promising areas for addressing patient lifestyle changes in SSA.

For peer review only

Many studies utilised KETs to facilitate task redistribution, which is a well evidenced strategy to improving healthcare provision in areas with low numbers of qualified doctors/specialists, for which the body of evidence relating to hypertension is growing.[60-65] Mobile technology provided decision support and record keeping tools aiming to empower non physician workers in providing primary hypertension care. While perceptions and feedback from clinical staff and patients was overall positive, several key areas were consistently reported as major challenges for uptake of KETs. Fundamental issues in infrastructure are still a barrier to mobile technology for healthcare, evidenced by reported issues with internet, network and power coverage. In addition, several articles reported concerns around the ability of patients to use the technology and understand the information which was relayed, with calls for future research to investigate the feasibility and efficacy of audio visual rather than text communication.[34 42] Such findings have informed further development of the PINGS intervention, with upcoming phase III trials using a reductionist approach, removing a smartphone component and replacing with phone calls and audio and text messages.[66]

A notable finding was that few studies reported statistically significant benefits of KET based interventions. Authors speculated that small sample sizes,[40] subject selection,[34 42] failed SMS delivery,[34] study design such that all patients received reminders,[32 45] free medication[32] or financial incentives[41] could have contributed to this. Likely reflecting difficulty in retaining patients in care in SSA. It was also observed that interventions proved most effective among the highest risk groups, where it may be easiest to detect positive changes. Although not always found to be significant, reductions in BP were observed which, although modest, would, from a clinical perspective be anticipated to impact CVD development on a population level.[67] Our findings also indicated strategies using SMS to promote positive patient behaviour changes were highly successful.[34 39 47] It remains to be seen however, whether self-reported behaviour changes translate into objective improvements in blood pressure reduction.

Strengths and limitations of this study

A major limitation of this systematic review was the heterogeneity of the included studies, which did not allow for quantitative synthesis of outcomes/results. Since this study also failed to identify any reports of use of AI, it is possible that extending the search beyond the scientific literature may have found cases where AI was intrinsic as part of manufactured technology already being used for healthcare in SSA. A strength of this study is that it is the first systematic review concerning use of KETs for hypertension healthcare in SSA, and in this way provides a comprehensive overview of the current state of the art and indicates gaps to be addressed in future research.

Conclusion

Our study indicates there is limited research on use of KETs for hypertension in SSA, particularly we did not identify any studies using AI. The study demonstrates that mobile health strategies provided positive impact on BP control, health knowledge and treatment adherence. Further, stakeholder perceptions towards technology for hypertension prevention and management were positive. Therefore, further primary studies should be conducted, with an emphasis on objective measures such as BP reduction or BP control. It remains to be seen whether AI may also prove beneficial, such as through development of further diagnostic aids, or boosting signals from cheap easily manufactured sensors.

Contributors:
KS, LP and FC designed the study. LP and FC coordinated and supervised the study. KS, BO, LP and FC designed the data collection and methodology. KS and BO Screened records, extracted data, and assessed quality. KS wrote the original draft. All authors critically revised and edited the manuscript.

Declaration of interests: We declare no competing interests

I, the Submitting Author has the right to grant and does grant on behalf of all authors of the Work (as defined in the below author licence), an exclusive licence and/or a non-exclusive licence for contributions from authors who are: i) UK Crown employees; ii) where BMJ has agreed a CC-BY licence shall apply, and/or iii) in accordance with the terms applicable for US Federal Government officers or employees acting as part of their official duties; on a worldwide, perpetual, irrevocable, royalty-free basis to BMJ Publishing Group Ltd ("BMJ") its licensees and where the relevant Journal is co-owned by BMJ to the co-owners of the Journal, to publish the Work in BMJ Open and any other BMJ products and to exploit all rights, as set out in our licence.

Licence statement
The Submitting Author accepts and understands that any supply made under these terms is made by BMJ to the Submitting Author unless you are acting as an employee on behalf of your employer or a postgraduate student of an affiliated institution which is paying any applicable article publishing charge ("APC") for Open Access

articles. Where the Submitting Author wishes to make the Work available on an Open Access basis (and intends to pay the relevant APC), the terms of reuse of such Open Access shall be governed by a Creative Commons licence – details of these licences and which Creative Commons licence will apply to this Work are set out in our licence referred to above.

For peer review only

References

1. (WHO) WHO. Cardiovascular diseases (CVDs) [Fact sheet]. 2017 [Available from: https://www.who.int/en/news-room/fact-sheets/detail/cardiovascular-diseases-(cvds).
2. WHO. The top 10 causes of death [cited 2021. Available from: https://www.who.int/news-room/fact-sheets/detail/the-top-10-causes-of-death.
3. Dzudie A, Rayner B, Ojji D, et al. Roadmap to achieve 25% hypertension control in Africa by 2025. *Cardiovascular journal of Africa* 2017;28(4):262-72. doi: 10.5830/CVJA-2017-040
4. Nyirenda MJ. Non-communicable diseases in sub-Saharan Africa: understanding the drivers of the epidemic to inform intervention strategies. *Int Health* 2016;8(3):157-8. doi: 10.1093/inthealth/ihw021 [published Online First: 2016/05/15]
5. Ataklte F, Erqou S, Kaptoge S, et al. Burden of Undiagnosed Hypertension in Sub-Saharan Africa. *Hypertension* 2015;65(2):291-98. doi: doi:10.1161/HYPERTENSIONAHA.114.04394
6. Piette JD, List J, Rana GK, et al. Mobile Health Devices as Tools for Worldwide Cardiovascular Risk Reduction and Disease Management. *Circulation* 2015;132(21):2012-27. doi: 10.1161/CIRCULATIONAHA.114.008723
7. Chang LW, Njie-Carr V, Kalenge S, et al. Perceptions and acceptability of mHealth interventions for improving patient care at a community-based HIV/AIDS clinic in Uganda: a mixed methods study. *AIDS Care* 2013;25(7):874-80. doi: 10.1080/09540121.2013.774315 [published Online First: 2013/03/05]
8. Vélez O, Okyere PB, Kanter AS, et al. A usability study of a mobile health application for rural Ghanaian midwives. *J Midwifery Womens Health* 2014;59(2):184-91. doi: 10.1111/jmwh.12071 [published Online First: 2014/01/10]
9. Bruxvoort K, Festo C, Kalolella A, et al. Cluster randomized trial of text message reminders to retail staff in tanzanian drug shops dispensing artemether-lumefantrine: effect on dispenser knowledge and patient adherence. *Am J Trop Med Hyg* 2014;91(4):844-53. doi: 10.4269/ajtmh.14-0126 [published Online First: 2014/07/07]
10. Mahmud N, Rodriguez J, Nesbit J. A text message-based intervention to bridge the healthcare communication gap in the rural developing world. *Technology and Health Care* 2010;18:137-44. doi: 10.3233/THC-2010-0576
11. Kamanga A, Moono P, Stresman G, et al. Rural health centres, communities and malaria case detection in Zambia using mobile telephones: a means to detect potential reservoirs of infection in unstable transmission conditions. *Malar J* 2010;9:96. doi: 10.1186/1475-2875-9-96 [published Online First: 2010/04/20]
12. Rajput ZA, Mbugua S, Amadi D, et al. Evaluation of an Android-based mHealth system for population surveillance in developing countries. *J Am Med Inform Assoc* 2012;19(4):655-9. doi: 10.1136/amiajnl-2011-000476 [published Online First: 2012/03/01]
13. World Health A. mHealth: use of appropriate digital technologies for public health: report by the Director-General. Geneva: World Health Organization, 2018.
14. WHO. A resolution on eHealth adopted at the 58th World Health Assembly, 2005.
15. GSMA. Mobile Economy Sub-Saharan Africa, 2019.
16. White A, Thomas DS, Ezeanochie N, et al. Health Worker mHealth Utilization: A Systematic Review. *Comput Inform Nurs* 2016;34(5):206-13. doi: 10.1097/cin.0000000000000231 [published Online First: 2016/03/10]
17. Braun R, Catalani C, Wimbush J, et al. Community health workers and mobile technology: a systematic review of the literature. *PLoS One* 2013;8(6):e65772. doi: 10.1371/journal.pone.0065772 [published Online First: 2013/06/19]
18. Osei E, Kuupiel D, Vezi PN, et al. Mapping evidence of mobile health technologies for disease diagnosis and treatment support by health workers in sub-Saharan Africa: a scoping review. *BMC Medical Informatics and Decision Making* 2021;21(1):11. doi: 10.1186/s12911-020-01381-x
19. Stephani V, Opoku D, Quentin W. A systematic review of randomized controlled trials of mHealth interventions against non-communicable diseases in developing countries. *BMC Public Health* 2016;16(1):572. doi: 10.1186/s12889-016-3226-3
20. Bloomfield GS, Vedanthan R, Vasudevan L, et al. Mobile health for non-communicable diseases in Sub-Saharan Africa: a systematic review of the literature and strategic framework for research. *Global Health* 2014;10:49. doi: 10.1186/1744-8603-10-49 [published Online First: 2014/06/15]
21. Jiang F, Jiang Y, Zhi H, et al. Artificial intelligence in healthcare: past, present and future. *Stroke and Vascular Neurology* 2017;2(4):230. doi: 10.1136/svn-2017-000101
22. Wahl B, Cossy-Gantner A, Germann S, et al. Artificial intelligence (AI) and global health: how can AI contribute to health in resource-poor settings? *BMJ Global Health* 2018;3(4):e000798. doi: 10.1136/bmjgh-2018-000798

For peer review only - http://bmjopen.bmj.com/site/about/guidelines.xhtml

23. Alami H, Rivard L, Lehoux P, et al. Artificial intelligence in health care: laying the Foundation for Responsible, sustainable, and inclusive innovation in low- and middle-income countries. *Globalization and Health* 2020;16(1):52. doi: 10.1186/s12992-020-00584-1

24. Moher D, Liberati A, Tetzlaff J, et al. Preferred Reporting Items for Systematic Reviews and Meta-Analyses: The PRISMA Statement. *PLOS Medicine* 2009;6(7):e1000097. doi: 10.1371/journal.pmed.1000097

25. Akl E, Altman D, Aluko P, et al. Cochrane Handbook for Systematic Reviews of Interventions2019.

26. Wallace BC, Dahabreh IJ, Trikalinos TA, et al. Closing the Gap between Methodologists and End-Users: R as a Computational Back-End. *Journal of Statistical Software; Vol 1, Issue 5 (2012)* 2012

27. Hong QN, Gonzalez-Reyes A, Pluye P. Improving the usefulness of a tool for appraising the quality of qualitative, quantitative and mixed methods studies, the Mixed Methods Appraisal Tool (MMAT). *J Eval Clin Pract* 2018;24(3):459-67. doi: 10.1111/jep.12884 [published Online First: 2018/02/22]

28. Kingue S, Angandji P, Menanga AP, et al. Efficiency of an intervention package for arterial hypertension comprising telemanagement in a Cameroonian rural setting: The TELEMED-CAM study. *The Pan African medical journal* 2013;15:153-53. doi: 10.11604/pamj.2013.15.153.2655

29. Ola-Olorun N, Afolabi MO, Oyebisi T, et al. Exploring Medicine Information Needs of Hypertensive Patients Using Short Message Service. *British Journal of Medicine and Medical Research* 2014;4:5368-80. doi: 10.9734/BJMMR/2014/8858

30. Joubert J, Nkomazana O, Mompati K, et al. A community survey of cardiovascular risk factors in an urban population in Botswana exploring potential for telemedicine. *European Research in Telemedicine / La Recherche Européenne en Télémédecine* 2014;3(3):95-103. doi: https://doi.org/10.1016/j.eurtel.2014.07.001

31. Leon N, Surender R, Bobrow K, et al. Improving treatment adherence for blood pressure lowering via mobile phone SMS-messages in South Africa: a qualitative evaluation of the SMS-text Adherence SuppoRt (StAR) trial. *BMC Fam Pract* 2015;16:80. doi: 10.1186/s12875-015-0289-7 [published Online First: 2015/07/04]

32. Bobrow K, Farmer AJ, Springer D, et al. Mobile Phone Text Messages to Support Treatment Adherence in Adults With High Blood Pressure (SMS-Text Adherence Support [StAR]): A Single-Blind, Randomized Trial. *Circulation* 2016;133(6):592-600. doi: 10.1161/circulationaha.115.017530 [published Online First: 2016/01/16]

33. Vedanthan R, Blank E, Tuikong N, et al. Usability and feasibility of a tablet-based Decision-Support and Integrated Record-keeping (DESIRE) tool in the nurse management of hypertension in rural western Kenya. *Int J Med Inform* 2015;84(3):207-19. doi: 10.1016/j.ijmedinf.2014.12.005 [published Online First: 2015/01/24]

34. Hacking D, Haricharan HJ, Brittain K, et al. Hypertension Health Promotion via Text Messaging at a Community Health Center in South Africa: A Mixed Methods Study. *JMIR Mhealth Uhealth* 2016;4(1):e22. doi: 10.2196/mhealth.4569 [published Online First: 2016/03/12]

35. Haricharan HJ, Heap M, Hacking D, et al. Health promotion via SMS improves hypertension knowledge for deaf South Africans. *BMC Public Health* 2017;17(1):663. doi: 10.1186/s12889-017-4619-7

36. Kleczka B, Musiega A, Rabut G, et al. Rubber stamp templates for improving clinical documentation: A paper-based, m-Health approach for quality improvement in low-resource settings. *Int J Med Inform* 2018;114:121-29. doi: 10.1016/j.ijmedinf.2017.10.014 [published Online First: 2017/11/07]

37. Mannik JR, Figol A, Churchill V, et al. Community-based screening for cardiovascular risk using a novel mHealth tool in rural Kenya. *BMJ Health & Care Informatics* 2018;25(3):176. doi: 10.14236/jhi.v25i3.1012

38. Nelissen HE, Cremers AL, Okwor TJ, et al. Pharmacy-based hypertension care employing mHealth in Lagos, Nigeria - a mixed methods feasibility study. *BMC Health Serv Res* 2018;18(1):934. doi: 10.1186/s12913-018-3740-3 [published Online First: 2018/12/06]

39. Sarfo F, Treiber F, Gebregziabher M, et al. PINGS (Phone-Based Intervention Under Nurse Guidance After Stroke): Interim Results of a Pilot Randomized Controlled Trial. *Stroke* 2018;49(1):236-39. doi: 10.1161/strokeaha.117.019591 [published Online First: 2017/12/10]

40. Vedanthan R, Kamano JH, DeLong AK, et al. Community Health Workers Improve Linkage to Hypertension Care in Western Kenya. *J Am Coll Cardiol* 2019;74(15):1897-906. doi: 10.1016/j.jacc.2019.08.003 [published Online First: 2019/09/06]

41. Owolabi MO, Gebregziabher M, Akinyemi RO, et al. Randomized Trial of an Intervention to Improve Blood Pressure Control in Stroke Survivors. *Circ Cardiovasc Qual Outcomes* 2019;12(12):e005904. doi: 10.1161/circoutcomes.119.005904 [published Online First: 2019/12/07]

42. Sarfo FS, Treiber F, Gebregziabher M, et al. Phone-based intervention for blood pressure control among Ghanaian stroke survivors: A pilot randomized controlled trial. *Int J Stroke* 2019;14(6):630-38. doi: 10.1177/1747493018816423 [published Online First: 2018/11/23]

For peer review only

43. Nichols M, Singh A, Sarfo FS, et al. Post-intervention qualitative assessment of mobile health technology to manage hypertension among Ghanaian stroke survivors. *Journal of the Neurological Sciences* 2019;406:116462. doi: https://doi.org/10.1016/j.jns.2019.116462
44. Cremers AL, Alege A, Nelissen HE, et al. Patients' and healthcare providers' perceptions and practices regarding hypertension, pharmacy-based care, and mHealth in Lagos, Nigeria: a mixed methods study. *Journal of hypertension* 2019;37(2):389-97. doi: 10.1097/HJH.0000000000001877
45. Barsky J, Hunter R, McAllister C, et al. Analysis of the Implementation, User Perspectives, and Feedback From a Mobile Health Intervention for Individuals Living With Hypertension (DREAM-GLOBAL): Mixed Methods Study. *JMIR Mhealth Uhealth* 2019;7(12):e12639. doi: 10.2196/12639 [published Online First: 2019/12/10]
46. Oduor E, Pang C, Wachira C, et al. Exploring Rural Community Practices in HIV Management for the Design of Technology for Hypertensive Patients Living with HIV. Proceedings of the 2019 on Designing Interactive Systems Conference. San Diego, CA, USA: Association for Computing Machinery, 2019:1595–606.
47. Adler AJ, Laar AK, Kotoh AM, et al. Barriers and facilitators to the implementation of a community-based hypertension improvement project in Ghana: a qualitative study of ComHIP. *BMC Health Services Research* 2020;20(1):67. doi: 10.1186/s12913-019-4774-x
48. Vedanthan R, Kumar A, Kamano JH, et al. Effect of Nurse-Based Management of Hypertension in Rural Western Kenya. *Glob Heart* 2020;15(1):77. doi: 10.5334/gh.856 [published Online First: 2020/12/11]
49. Aw M, Ochieng BO, Attambo D, et al. Critical appraisal of a mHealth-assisted community-based cardiovascular disease risk screening program in rural Kenya: an operational research study. *Pathog Glob Health* 2020;114(7):379-87. doi: 10.1080/20477724.2020.1816286 [published Online First: 2020/09/09]
50. Owoyemi A, Owoyemi J, Osiyemi A, et al. Artificial Intelligence for Healthcare in Africa. *Frontiers in Digital Health* 2020;2(6) doi: 10.3389/fdgth.2020.00006
51. Acheampong K, Nyamari JM, Ganu D, et al. Predictors of Hypertension among Adult Female Population in Kpone-Katamanso District, Ghana. *International Journal of Hypertension* 2019;2019:1876060. doi: 10.1155/2019/1876060
52. Masupe T, Thato J, Motlhatlhedi K, et al. A16812 Hypertension is Common and is Associated with Clustering of Cardiovascular Risk Factors Among Adults in Botswana. *Journal of Hypertension* 2018;36
53. Gebreselassie KZ, Padyab M. Epidemiology of Hypertension Stages in Two Countries in Sub-Sahara Africa: Factors Associated with Hypertension Stages. *International Journal of Hypertension* 2015;2015:959256. doi: 10.1155/2015/959256
54. Camara A, Baldé NM, Diakité M, et al. High prevalence, low awareness, treatment and control rates of hypertension in Guinea: results from a population-based STEPS survey. *J Hum Hypertens* 2016;30(4):237-44. doi: 10.1038/jhh.2015.92 [published Online First: 2015/08/28]
55. Pengpid S, Peltzer K. Prevalence, awareness, treatment and control of hypertension among adults in Kenya: cross-sectional national population-based survey. *East Mediterr Health J* 2020;26(8):923-32. doi: 10.26719/emhj.20.063 [published Online First: 2020/09/09]
56. Chowdhury MAB, Epnere K, Haque MA, et al. Urban rural differences in prevalence and risk factors of self-reported hypertension among Kenyan women: a population-based study. *Journal of Human Hypertension* 2020 doi: 10.1038/s41371-020-00435-x
57. Anjulo U, Haile D, Wolde A. Prevalence of Hypertension and Its Associated Factors Among Adults in Areka Town, Wolaita Zone, Southern Ethiopia. *Integr Blood Press Control* 2021;14:43-54. doi: 10.2147/ibpc.S295574 [published Online First: 2021/03/25]
58. Sarfo FS, Kyem G, Ovbiagele B, et al. One-Year Rates and Determinants of Poststroke Systolic Blood Pressure Control among Ghanaians. *Journal of Stroke and Cerebrovascular Diseases* 2017;26(1):78-86. doi: https://doi.org/10.1016/j.jstrokecerebrovasdis.2016.08.033
59. Nuamah K, Affran Bonful H, Danso Yeboah J, et al. Characteristics of Inpatient Hypertension Cases and Factors Associated with Admission Outcomes in Ashanti Region, Ghana: An Analytic Cross-Sectional Study. *Int J Hypertens* 2017;2017:6537956. doi: 10.1155/2017/6537956 [published Online First: 2018/01/24]
60. Anand TN, Joseph LM, Geetha AV, et al. Task sharing with non-physician health-care workers for management of blood pressure in low-income and middle-income countries: a systematic review and meta-analysis. *The Lancet Global Health* 2019;7(6):e761-e71. doi: 10.1016/S2214-109X(19)30077-4
61. Labhardt ND, Balo JR, Ndam M, et al. Task shifting to non-physician clinicians for integrated management of hypertension and diabetes in rural Cameroon: a programme assessment at two years. *BMC Health Serv Res* 2010;10:339. doi: 10.1186/1472-6963-10-339 [published Online First: 2010/12/15]
62. Iwelunmor J, Gyamfi J, Plange-Rhule J, et al. Exploring stakeholders' perceptions of a task-shifting strategy for hypertension control in Ghana: a qualitative study. *BMC Public Health* 2017;17(1):216. doi: 10.1186/s12889-017-4127-9

For peer review only - http://bmjopen.bmj.com/site/about/guidelines.xhtml

63. Ogedegbe G, Plange-Rhule J, Gyamfi J, et al. Health insurance coverage with or without a nurse-led task shifting strategy for hypertension control: A pragmatic cluster randomized trial in Ghana. *PLoS Med* 2018;15(5):e1002561. doi: 10.1371/journal.pmed.1002561 [published Online First: 2018/05/02]
64. Kengne AP, Awah PK, Fezeu LL, et al. Primary health care for hypertension by nurses in rural and urban sub-Saharan Africa. *J Clin Hypertens (Greenwich)* 2009;11(10):564-72. doi: 10.1111/j.1751-7176.2009.00165.x [published Online First: 2009/10/13]
65. Some D, Edwards JK, Reid T, et al. Task Shifting the Management of Non-Communicable Diseases to Nurses in Kibera, Kenya: Does It Work? *PLOS ONE* 2016;11(1):e0145634. doi: 10.1371/journal.pone.0145634
66. Sarfo FSA, A.; Bockarie, A.; Appiah, L.; Nguah, S. B.; Ayisi-Boateng, N. K.; Adamu, S.; Neizer, C.; Arthur, A.; Nyamekye, R.; Agyenim-Boateng, K.; Tagge, R.; Adusei-Mensah, N.; Ampofo, M.; Laryea, R.; Singh, A.; Amuasi, J. H.; Ovbiagele, B. Phone-Based Intervention under Nurse Guidance after Stroke (PINGS II) Study: Protocol for a Phase III Randomized Clinical Trial. *Journal of Stroke & Cerebrovascular Diseases* 2021;30(8):105888.
67. Ettehad D, Emdin CA, Kiran A, et al. Blood pressure lowering for prevention of cardiovascular disease and death: a systematic review and meta-analysis. *The Lancet* 2016;387(10022):957-67. doi: 10.1016/S0140-6736(15)01225-8

For peer review only

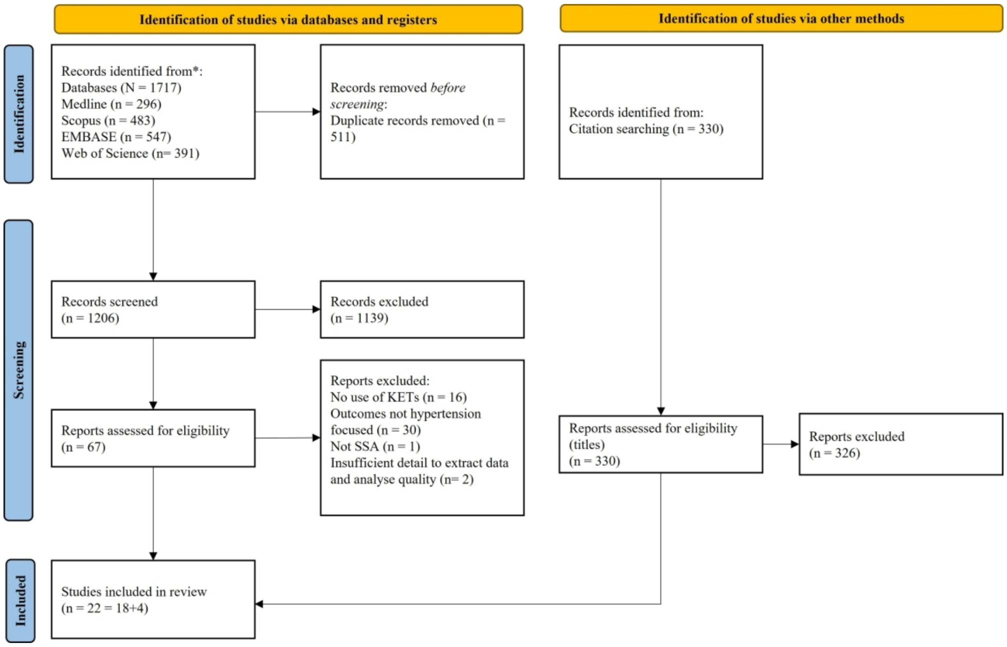

Figure 1. Study selection

257x164mm (300 x 300 DPI)

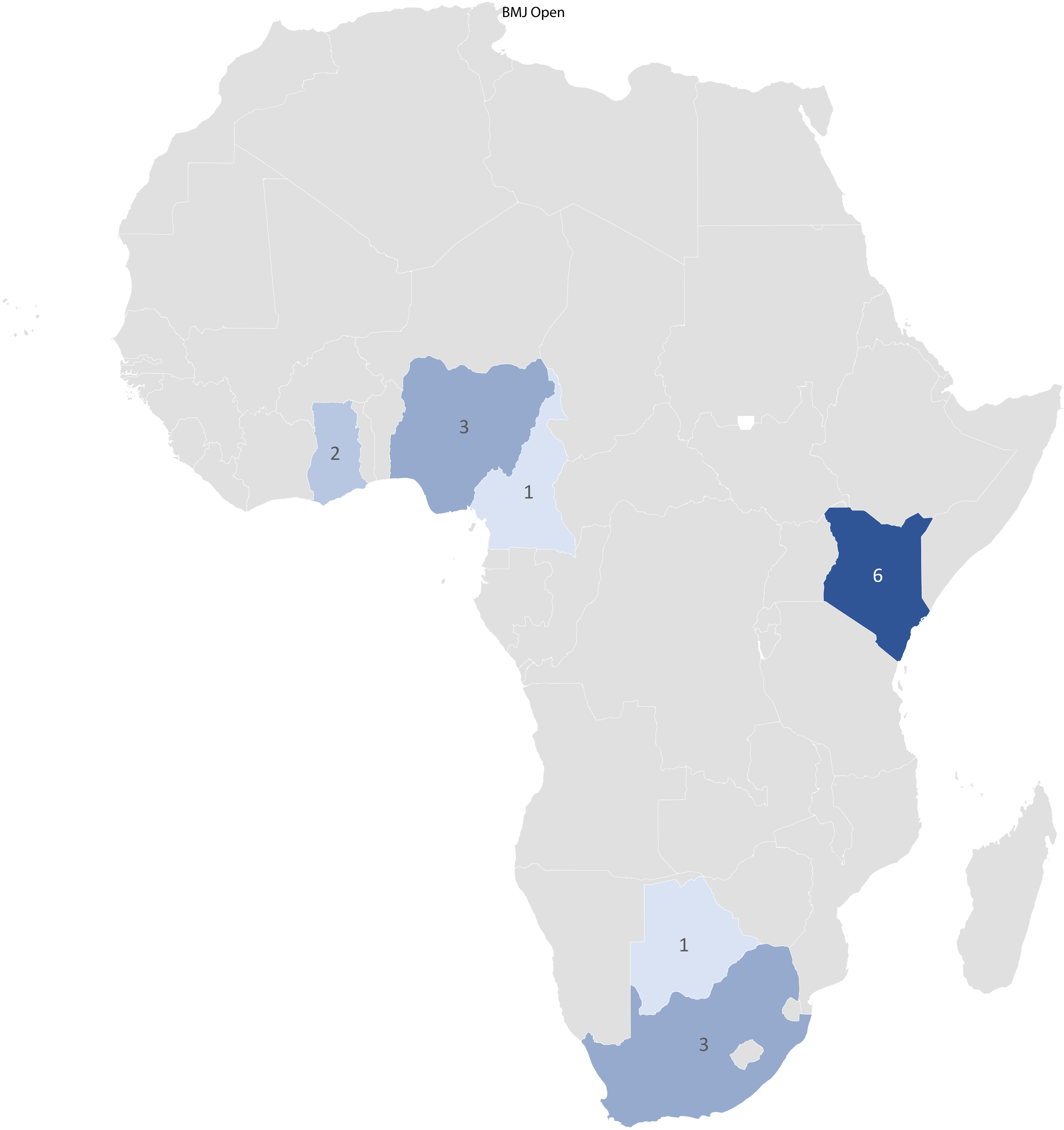
2
3
1
6
1
3
Powered by Bing
© GeoNames, Microsoft, TomTom, Wikipedia

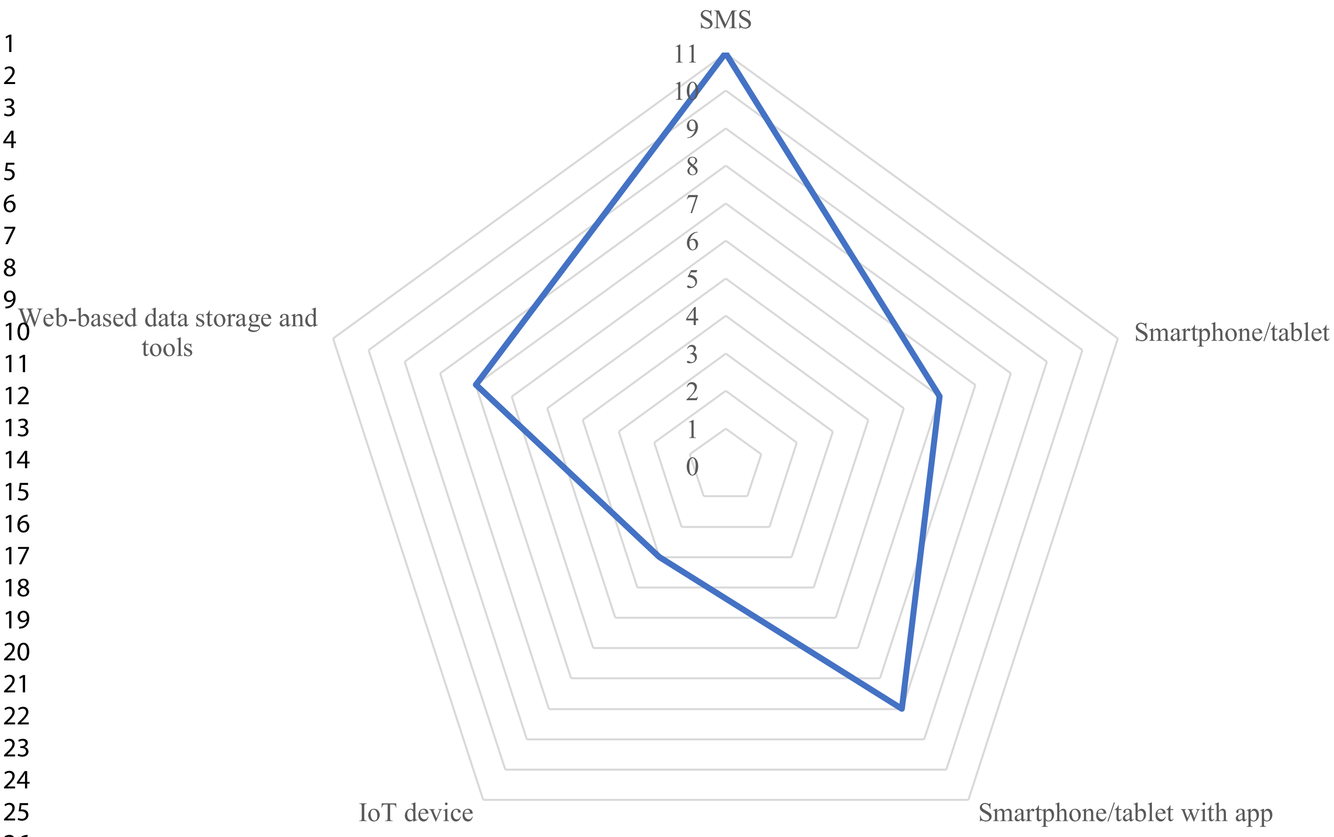
SMS
11
10
9
8
7
6
5
4
3
2
1
0
Smartphone/tablet
Smartphone/tablet with app
IoT device
Web-based data storage and tools

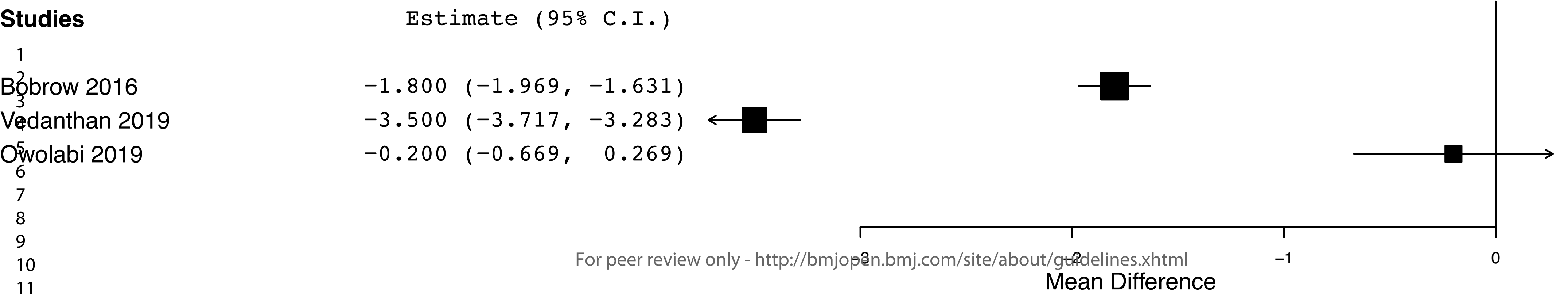

For peer review only - http://bmjopen.bmj.com/site/about/guidelines.xhtml

Test S1. Search terms

MEDLINE/Embase:
exp Cardiovascular Diseases/ or exp Hypertension/ or ("cardiovascular disease*" or CVD* or "hypertensi*" or "high blood pressure" or "blood pressure" or stroke or "heart disease").tw. and exp "Africa South of the Sahara"/ or (Sahara* or sub-Sahara* or "sub-Sahara* Africa" or SSA or Cameroon or "Central African Republic" or Chad or Congo or "democratic republic of the Congo" or "equatorial Guinea" or Gabon or "Sao Tome and Principe" or Burundi or Djibouti or Eritrea or Ethiopia or Kenya or Rwanda or Somalia or "south Sudan" or Sudan or Tanzania or Uganda or Angola or Botswana or Eswatini or Lesotho or Malawi or Mozambique or Namibia or Zambia or Zimbabwe or Benin or "Burkina Faso" or "Cabo Verde" or "Cote d'Ivoire" or Gambia or Ghana or Guinea or "Guinea-Bissau" or Liberia or Mali or Mauritania or Niger or Nigeria or Senegal or "Sierra Leone" or Togo).tw. and exp Artificial Intelligence/ or exp Artificial Intelligence/ or exp Cell Phone/ or exp "Internet of Things"/ or exp Telemedicine/ or ("artificial intelligence" or ai or "machine learning" or "deep learning" or "neural network*" or "reinforcement learning" or "naive bayes" or "decision tree" or "random forest" or "support vector machine" or "k-nearest neighbour" or "linear discriminant analysis" or classification or clustering or "supervised learning" or telehealth or telemedicine or mhealth or "mobile phone" or smartphone or "internet of things" or "cell phone").tw. OR (exp Artificial Intelligence/ or ("artificial intelligence" or ai or "machine learning").tw.) and ("logistic regression" or "logit regression" or "multiple regression").tw. and exp diagnosis/ or exp therapeutics exp treatment outcome/ or (prevent* or screen* or detection or diagnos* or management or treatment or control).tw.

Web of Science:
TS = (prevent* or screen* or diagnos* or treatment or therapeutics or management or control) and S =("artificial intelligence" or ai or "machine learning" or "deep learning" or "neural network*" or "reinforcement learning" or "naive bayes" or "decision tree" or "random forest" or "support vector machine" or "k-nearest neighbour" or "linear discriminant analysis" or classification or clustering or "supervised learning" or telehealth or telemedicine or mhealth or "mobile phone" or smartphone or "internet of things" or "cell phone") OR (TS=("artificial intelligence" or ai or "machine learning") AND TS=("logistic regression" or "logit regression" or "multiple regression")) and TS = ("Africa south of Sahara" or "Sahara" or "sub-Sahara*" or "sub-Sahara* Africa" or SSA or Cameroon or “Central African Republic” or Chad or Congo or “democratic republic of the Congo” or “equatorial guinea” or Gabon or “Sao Tome and Principe” or Burundi or Djibouti or Eritrea or Ethiopia or Kenya or Rwanda or Somalia or “South Sudan” or Sudan or Tanzania or Uganda or Angola or Botswana or Eswatini or Lesotho or Malawi or Mozambique or Namibia or Zambia or Zimbabwe or Benin or “Burkina Faso” or “Cabo Verde” or “Cote d’Ivoire” or Gambia or Ghana or Guinea or “Guinea-Bissau” or Liberia or Mali or Mauritania or Niger or Nigeria or Senegal or “Sierra Leone” or Togo) and TS=(cardiovascular disease CVD* or hypertensi* or high blood pressure or blood pressure or stroke or heart disease)

Scopus:
((TITLE-ABS-KEY ("cardiovascular disease*" OR cvd* OR "hypertensi*" OR "high blood pressure" OR "blood pressure" OR stroke OR "heart disease"))) AND (TITLE-ABS-KEY ("artificial intelligence" OR ai OR "machine learning" OR "deep learning" OR "neural network*" OR "reinforcement learning" OR "naive bayes" OR "decision tree" OR "random forest" OR "support vector machine" OR "k-nearest neighbour" OR "linear discriminant analysis" OR classification OR clustering OR "supervised learning" OR telehealth OR telemedicine OR mhealth OR "mobile phone" OR smartphone OR "internet of things" OR "cell phone") OR (TITLE-ABS-KEY ("artificial intelligence" OR ai OR "machine learning") AND TITLE-ABS-KEY ("logistic regression" OR "logit regression" OR "multiple regression"))) AND (TITLE-ABS-KEY ("Africa south of sahara" OR "sahara" OR "sub-sahara*" OR "sub-sahara* africa" OR ssa OR cameroon OR "Central African Republic" OR chad OR congo OR "democratic republic of the Congo" OR "equatorial guinea" OR gabon OR "Sao Tome and Principe" OR burundi OR djibouti OR eritrea OR ethiopia OR kenya OR rwanda OR somalia OR "South Sudan" OR sudan OR tanzania OR uganda OR angola OR botswana OR eswatini OR lesotho OR malawi OR mozambique OR namibia OR zambia OR zimbabwe OR benin OR "Burkina Faso" OR "Cabo Verde" OR "Cote d'Ivoire" OR gambia OR ghana OR guinea OR "Guinea-Bissau" OR liberia OR mali OR mauritania OR niger OR nigeria OR senegal OR "Sierra Leone" OR togo)) AND (TITLE-ABS-KEY (prevent* OR screen* OR diagnos* OR treatment OR therapeutics OR management OR control))

For peer review only

Table S1. Articles excluded on reading full text.

Authors	Source	Year	Title	Reason:
T.; Njuguna Mercer, B.; Bloomfield, G. S.; Dick, J.; Finkelstein, E.; Kamano, J.; Mwangi, A.; Naanyu, V.; Pastakia, S. D.; Valente, T. W.; Vedanthan, R.; Akwanalo, C.	Trials	2019	Strengthening Referral Networks for Management of Hypertension Across the Health System (STRENGTHS) in western Kenya: a study protocol of a cluster randomized trial	No KETs
I.; Joloba Ddumba, S.; Kakande, B.	Transactions of the Royal Society of Tropical Medicine and Hygiene	2019	Motivations for participating in the use of mobile smart phone app focusing on monitoring of hypertension among older persons in Uganda	Insufficient detail
F. S.; Adusei Sarfo, N.; Ampofo, M.; Kpeme, F. K.; Ovbiagele, B.	Journal of the Neurological Sciences	2018	Pilot trial of a tele-rehab intervention to improve outcomes after stroke in Ghana: A feasibility and user satisfaction study	Not hypertension
F. S.; Adamu Sarfo, S.; Awuah, D.; Sarfo-Kantanka, O.; Ovbiagele, B.	Journal of the Neurological Sciences	2017	Potential role of tele-rehabilitation to address barriers to implementation of physical therapy among West African stroke survivors: A cross-sectional survey	Not hypertension
F.; Pudney Lorgat, E.; van Deventer, H.; Chitsaz, S.	Cardiovascular Journal of Africa	2012	Robotically controlled ablation for atrial fibrillation: the first real-world experience in Africa with the Hansen robotic system	Not hypertension
R.; Kamano Vedanthan, J. H.; Naanyu, V.; Delong, A. K.; Were, M. C.; Finkelstein, E. A.; Menya, D.; Akwanalo, C. O.; Bloomfield, G. S.; Binanay, C. A.; Velazquez, E. J.; Hogan, J. W.; Horowitz, C. R.; Inui, T. S.; Kimaiyo, S.; Fuster, V.	Trials [Electronic Resource]	2014	Optimizing linkage and retention to hypertension care in rural Kenya (LARK hypertension study): study protocol for a randomized controlled trial	No KETs
E.; Nyota Oduor, T.; Wachira, C.; Osebe, S.; Remy, S. L.; Walcott, A.	Journal	Year	Medication Management Companion (MMC) for a rural kenyan community	No KETs
J. T.; Tham Kamwesiga, K.; Guidetti, S.	Disability and Rehabilitation	2017	Experiences of using mobile phones in everyday life among persons with stroke and their families in Uganda - a qualitative study	Not hypertension
J. T.; Eriksson Kamwesiga, G. M.; Tham, K.; Fors, U.; Ndiwalana, A.; von Koch, L.; Guidetti, S.	Global Health	2018	A feasibility study of a mobile phone supported family-centred ADL intervention, F@ce TM, after stroke in Uganda	Not hypertension
F. K.; Adoubi Diby, A.; Gnaba, A.; Ouattara, P.; Ayegnon, K. G.; Boidy, K.; Azagoh-Kouadio, R.; Meneas, G.; Manga, D.; Coulibaly, A.; Sall, F.; Nguessan, E.; Ehui, E.; Yangni-Angate, K. H.	European Research in Telemedicine	2015	Tele-expertise in the interpretation of the electrocardiogram of a black African population in the Ivory Coast (sub-Saharan Africa)	Not hypertension
G. S.; Wang Bloomfield, T. Y.; Boulware, L. E.; Califf, R. M.; Hernandez, A. F.; Velazquez, E. J.; Peterson, E. D.; Li, J. S.	Global heart	2015	Implementation of management strategies for diabetes and hypertension: from local to global health in cardiovascular diseases	No KETs
T.; Dewyer Aliku, A.; Namuyonga, J.; Ssinabulya, I.; Kamarembo, J.; Okello, E.; Bua, B.; Asiimwe, A.; Odong, F.; Akech, R.; Beaton, A.; DeStigter, K.; Lwabi, P.; Sable, C.	Global Heart	2018	Telemedicine Support of Cardiac Care In Northern Uganda: Leveraging Hand-held Echocardiography and Task Shifting	Not hypertension
M.; Sarfo Nichols, F. S.; Singh, A.; Qanungo, S.; Treiber, F.; Ovbiagele, B.; Saulson, R.; Patel, S.; Jenkins, C.	American Journal of the Medical Sciences	2017	Assessing Mobile Health Capacity and Task Shifting Strategies to Improve Hypertension Among Ghanaian Stroke Survivors	Not hypertension
H. L.; Duhig Nathan, K.; Vousden, N.; Lawley, E.; Seed, P. T.; Sandall, J.; Bellad, M. B.; Brown, A. C.; Chappell, L. C.; Goudar, S. S.; Gidiri, M. F.; Shennan, A. H.; Cradle- Trial Collaboration Grp	Trials	2018	Evaluation of a novel device for the management of high blood pressure and shock in pregnancy in low-resource settings: study protocol for a stepped-wedge cluster-randomised controlled trial (CRADLE-3 trial)	No KETs
J. S.; Inyiama Igwe, H. C.; Alo, U. R.; Ajah, I. A.	International Journal of Scientific and Technology Research	2020	Interpretation of eeg recordings for the purpose of diagnosing stroke disease	Not hypertension
J. S.; Inyiama Igwe, H. C.; Alo, U. R.; Ajah, I. A.	Journal		Classification of human brain signal for diagnosis of stroke disease using artificial neural network	Not hypertension
O.; Olabode Olabode, B. T.	Journal of Computer Science	2012	Cerebrovascular accident attack classification using multilayer feed forward artificial neural network with back propagation error	Not hypertension
N.; Imberti Maurizi, J. F.; Faragli, A.; Targetti, M.; Baldini, K.; Sall, A.; Cisse, A.; Gigli Berzolari, F.; Borrelli, P.; Avvantaggiato, F.; Perlini, S.; Marchionni, N.; Cecchi, F.; Parigi, G. B.; Olivotto, I.	European Heart Journal	2016	Comparative analysis of a 4-lead portable smartphone-based versus standard 12-lead electrocardiograph for cardiovascular screening in low-income settings	Not hypertension
N.; Faragli Maurizi, A.; Imberti, J.; Briante, N.; Targetti, M.; Baldini, K.; Sall, A.; Cisse, A.; Berzolari, F. G.; Borrelli, P.; Avvantaggiato, F.; Perlini, S.; Marchionni, N.; Cecchi, F.; Parigi, G.; Olivotto, I.	International Journal of Cardiology	2017	Cardiovascular screening in low-income settings using a novel 4-lead smartphone-based electrocardiograph (D-Heart®)	Not hypertension
J. J.; Salehian Manolakos, O.; Kraeker, C.; Manolakos, L.; Hunter, C. J.	Canadian Journal of Cardiology	2015	Rheumatic heart disease screening in Windhoek Namibia using portable echocardiography: A pilot project	Not hypertension
G. F.; Shirk Evans, A.; Muturi, P.; Soliman, E. Z.	Global Heart	2017	Feasibility of Using Mobile ECG Recording Technology to Detect Atrial Fibrillation in Low-Resource Settings	Not hypertension

For peer review only

Table S2. Quality analysis

	RefID	31	32	40	28	41	34	39	42	43	47	33	48	38	44	37	49	29	36	35	46	30	45
	First author	Leon	Bobrow	Vedanthan	Kingue	Owolabi	Hacking	Sarfo	Sarfo	Nichols	Adler	Vedanthan	Vedanthan	Nelissen	Cremers	Mannik	Aw	Ola-Olorun	Kleczka	Haricharan	Odour	Joubert	Barsky
	Year	2015	2016	2019	2013	2019	2016	2018	2019	2019	2020	2015	2020	2018	2019	2018	2020	2014	2018	2017	2019	2014	2019
	S1. Are there clear research questions?	Yes	Yes	Yes	Yes	Yes	Yes	Yes	Yes	Yes	Yes	Yes	Yes	Yes	Yes	Yes	Yes	Yes	Yes	Yes	Yes	Yes	Yes
SCREENING QUESTIONS	S2. Do the collected data allow to address the research questions?	Yes	Yes	Yes	Yes	Yes	Yes	Yes	Yes	Yes	Yes	Yes	Yes	Yes	Yes	Yes	Yes	Yes	Yes	Yes	Yes	Yes	Yes
	1.1. Is the qualitative approach appropriate to answer the research question?	Yes					Yes			Yes	Yes	Yes		Yes	Yes	Yes	Yes	Yes		Yes	Yes		Yes
	1.2. Are the qualitative data collection methods adequate to address the research question?	Yes					Yes			Yes	Yes	Yes		Yes	Yes	Yes	Yes	Yes		Yes	Yes		Yes
	1.3. Are the findings adequately derived from the data?	Yes					Yes			Yes	Yes	Yes		Yes	Yes	Can't tell	Yes	Yes		Yes	Yes		Can't tell
	1.4. Is the interpretation of results sufficiently substantiated by data?	Yes					Yes			Yes	Yes	Yes		Yes	Yes	No	Yes	Yes		Yes	Yes		Can't tell
1. QUALITATIVE STUDIES	1.5. Is there coherence between qualitative data sources, collection, analysis and interpretation?	Yes					Yes			Yes	Yes	Yes		Yes	Yes	Yes	Yes	Yes		Yes	Yes		Can't tell
	2.1. Is randomization appropriately performed?		Yes	No	No	Yes	No	Yes	Yes														Yes
	2.2. Are the groups comparable at baseline?		Yes	No	No	Yes	Yes	Yes	Yes														Can't tell
	2.3. Are there complete outcome data?		Yes	No	Yes	Yes	No	Yes	Yes														Can't tell
	2.4. Are outcome assessors blinded to the intervention provided?		Yes	No	No	Yes	Can't tell	Yes	Yes														Can't tell
2. RANDOMIZED CONTROLLED TRIALS	2.5 Did the participants adhere to the assigned intervention?		Yes	Can't tell	Yes	Yes	Yes	Yes	Yes														Can't tell
	3.1. Are the participants representative of the target population?												Yes	Yes	Yes			Yes	Yes	No			
	3.2. Are measurements appropriate regarding both the outcome and intervention (or exposure)?												Yes	No	Yes			Yes	Yes	Yes			
	3.3. Are there complete outcome data?												No	No	No			Yes	Yes	No			
	3.4. Are the confounders accounted for in the design and analysis?												Yes	Yes	Can't tell			Can't tell	Yes	Yes			
3. NON-RANDOMIZED STUDIES	3.5. During the study period, is the intervention administered (or exposure occurred) as intended?												No	No	Yes			Yes	Yes	Yes			
	4.1. Is the sampling strategy relevant to address the research question?															Yes	Yes					Yes	
	4.2. Is the sample representative of the target population?															Yes	No					Yes	
	4.3. Are the measurements appropriate?															Yes	Yes					Yes	
	4.4. Is the risk of nonresponse bias low?														Yes	Yes					Yes		
4. QUANTITATIVE DESCRIPTIVE STUDIES	4.5. Is the statistical analysis appropriate to answer the research question?														Yes	Yes					Yes		
	5.1. Is there an adequate rationale for using a mixed methods design to address the research question?					Yes							Yes	Yes	Yes	Yes	Yes		Yes			Yes	
	5.2. Are the different components of the study effectively integrated to answer the research question?					Yes							Yes	Yes	Yes	Yes	Yes		Yes			Yes	
	5.3. Are the outputs of the integration of qualitative and quantitative components adequately interpreted?					Yes							Yes	Yes	Can't tell	Yes	Yes		Yes			Can't tell	
	5.4. Are divergences and inconsistencies between quantitative and qualitative results adequately addressed?					Yes							Yes	Can't tell	Yes	Yes	Yes		Yes			Can't tell	
5. MIXED METHODS STUDIES	5.5. Do the different components of the study adhere to the quality criteria of each tradition of the methods involved?					No							No	Yes	Yes	Yes	Yes		No			No	
	% criteria met	100	100	0	40	90	70	100	100	100	100	100	70	73.33333333	82.35294118	86.66666667	92.30769231	90	90	80	100	62.5	30

Table S3. Extracted baseline and endpoint systolic blood pressure readings for randomised controlled trials

Author	Title	Year	Duration	Intervention type	Primary outcome	Secondary outcome	Monitoring tools	Groups	Number of participants (baseline)	Number of participants (endpoint)	Groups	SBP baseline	SD	SBP endpoint	SD	Groups	Change in SBP (From baseline-endpoint values)	SD (σd = sqrt(σ1^2 / n1 + σ2^2 / n2))
Bobrow et al.	Mobile Phone Text Messages to Support Treatment Adherence in Adults With High Blood Pressure (StAR): A Single-Blind, Randomized Trial	2016	12 months	SMS-Text Adherence suppoRt. Patients with high BP received SMS. Information only: Messages for motivation for medicine collection/taking or attending clinic, messages for education of hypertension. Interactive: Received same messages and were able to respond for changing an appointment, and changing the timing and language of the text-messages	Change in Systolic Blood Pressure at 12-months from baseline	Proportion of participants achieving mean SBP <140mmHg and mean DBP <90mmHg.<br>Health status (measured by self report questionnarire).<br>Proportion of scheduled clinic appointments attended.<br>Retention in clinical care. Satisfaction with clinic services and care.<br>Hospital admissions.<br>Self-reported adherence to medication.<br>Basic hypertension knowledge.<br>Number and type of medication changes made during trial.<br>Number of clinic visits.	validated oscillometric device. Recorded six sequential readings at three-minute intervals. The mean blood pressure was calculated by discarding the initial reading and calculating the mean from the five remaining readings.	Usual Care (UC)<br>Information Only (IO)<br>Interactive (I)										
								**UC**	457	396	**UC**	135.4	17.6	134.3	17.3	**UC**	-1.1	1.20
								**IO**	457	406	**IO**	135.1	16.9	132.1	16.6	**IO**	-3	1.14
								**I**	458	394	**I**	135.6	18.1	132.7	17.5	**I**	-2.9	1.22
Vedanthan et al.	Community Health Workers Improve Linkage to Hypertension Care in Western Kenya	2019	15 months	Patients with high BP. Tailored behavioral communication + mHealth. Paper based: Community health worker ugave tailored behavioural and motivational engagement.<br>Smartphone: CHW did same as PB, but had real-time decision support and data entry linked to electronic health record.<br>Cluster randomised	Co-primary outcomes:<br>Linkage to care<br>Change in SBP	N/A	automated Omron blood pressure machine, standard protocol (as described by World Health Organisation)	Usual Care (UC)<br>Paper Based (PB)<br>Smartphone (SP)										
								**UC**	422	355	**UC**	158.4	19.6	150	22.9	**UC**	-8.4	1.55
								**PB**	451	395	**PB**	158.3	20.3	150.2	21.6	**PB**	-8.1	1.45
								**SP**	465	356	**SP**	161.3	18.5	149.4	20.8	**SP**	-11.9	1.40
Owolabi et al.	Randomized Trial of an Intervention to Improve Blood Pressure Control in Stroke Survivors	2019	12 months	Patients with stroke-onset within one year.<br>Intervention: chronic care model components of delivery system redesign (increased follow-up visits, pre-appointment phone texts), self-management support (patient report card, post-clinic follow-up phone texts, waiting room educational video), and clinical information systems (patient report card as part of medical chart, hospital registry).	Mean change in systolic blood pressure at 12 months	N/A	BP measurements were obtained and averaged from each subject with use of the Omron HEM-907XL26 according to a standardized protocol provided by the manufacturer about cuff size, cuff application, body position, and time intervals when taking a measurement.											
								**UC (high BP)**	**79**	**74**	**UC**	159.1	23.8	148.5	22.8	**UC**	-10.6	3.77
								**Intervention (high BI**	**89**	**84**	**Intervention**	157.3	19.6	145.1	22.6	**Intervention**	-12.2	3.22
								**UC (all records)**	**199**	**188**	**UC**	138.1	24.5	136.2	21.2	**UC**	-1.9	2.33
								**Intervention (all reco**	**199**	**186**	**Intervention**	138.6	22.9	136.5	22.3	**Intervention**	-2.1	2.30

For peer review only

Table S4. Logistic regression study characteristics

Title	Type of study	Country/ location	Population	Age (mean, sd)	Other diseases	Hypertension definition	No. patients	AI method	Hypertension prevalence	Predictors	Parameters
Predictors of Hypertension among Adult Female Population in Kpone-Katamanso District, Ghana	Prospective cross sectionalal study	Ghana, Kpone-Katamanso District	Female adults (>=25)	49.6±13.3	Excluded pregnant or unable to fill out form	systolic blood pres- sure ≥140mmHg and diastolic blood pressure ≥90mmHg or both	216	Multivariable logistic regression analysis. Adjusted Odds Ratio (AOR) with a 95 % Confidence Interval (CI) was computed to show the level of certainty. A p value of <0.05 was considered as statistically significant.	73 (33.8%) hypertensive, 26 (36.0%) previously diagnosed and 47 (64.0%) newly diagnosed	Increasing age (65+ years [AOR =5.13, (95% CI: 2.20-11.99), p<0.001]), obesity (BMI) ≥30 kg/m2 were two times as likely as those with normal weight to be hypertensive [AOR 2.38, (95% CI; 1.14-4.95, p<0.02)]), and women who did not consume fresh fruits daily (three times as likely as those women who consume fresh fruit daily to be hypertensive [AOR 3.17 (95% CI; 1.05-9.55, p<0.04)])	The association between hypertension and the independent variables was investigated by using the binary logistic regression and p values of <0.7 were considered as a variable selection criterion. Variables with a p value less than 0.7 were entered in the multivariable analysis to control the potential effect of confounders.
Hypertension is common and is associated with clustering of cardiovascular risk factors among adults in botswana	Prospective cross sectionalal study	Botswana (2 cities, 3 villages)	Adults (>18)	Not given	No details	current use of antihypertensive medication or mean systolic blood pressure ≥ 140 mmHg and/or mean diastolic blood pressure ≥ 90 mmHg	3587	Logistic regression	1012 (27.8%) hypertensive, 213 (21%) of those on antihypertensive medications. Male 30.7% vs Female 25.7%, p < 0.01. on antihypertensive medication (M 7.8% vs F11.8%, p < 0.01)	hypertension was associated w self-reported history of diabetes mellitus (p < 0.01), previous heart attack (p < 0.01) & previous stroke (p = 0.05). Overweight (p < 0.01), tobacco use (p = 0.01) and alcohol consump- tion (p = 0.05) were strongly associated with hypertension	No further details
Epidemiology of Hypertension Stages in Two Countries in Sub-Sahara Africa: Factors Associated with Hypertension Stages	Retrospective. Studied using global ageing and adult health (SAGE), WAVE 1 dataset	South Africa, Ghana	Adults (>18)	Not given	Diagnosed of hypertension previously or taking antihypertensive medication were excluded from analysis due to overlapping.	Defined using JNC7 blood pressure classification considering previous diagnosis and treatment	7545 (2980 from SA and 4565 from Ghana)	Multivariate multinomial logistic regression analysis	Weighted prevalence of prehypertension and hypertension in Ghana was 30.7% and 42.4%, respectively, and that of SA was 29.4% and 46%, respectively	After adjusting for the independent variables, only age (OR = 1.32, 95% CI: 1.14–1.53), income (OR = 1.9, 95% CI: 1.04–3.47), and BMI (OR = 1.16, 95% CI: 1.1–1.22) remained independent predictors for stage 1 hypertension in Ghana. In SA, age (OR = 2.27, 95% CI: 1.53–3.36), sex (OR = 0.28, 95% CI: 0.08–1), and BMI (OR = 1.15, 95% CI: 1.07–1.25) were independent predictors of stage 1 hypertension.	First, each variable was evaluated independently in a bivariate multinomial logistic regression analysis with different stages of hypertension as dependent variable to generate unadjusted OR with respon- dents' characteristics in each country separately. Those vari- ables with *p* values less than 0.2 were retained and entered into multinomial logistic regression model in ascending step-wise manner to determine variables that were independently associated with the stages of hypertension. A probability level of *p* < 0.05 was considered significant.
High prevalence, low awareness, treatment and control rates of hypertension in Guinea: results from a population-based STEPS survey	Prospective cross sectionalal study	Guinea (Rural Lower Guinea)	adult aged between 15 and 64 years	30.9 (± 13.0)	diabetes mellitus	systolic (and/or diastolic) blood pressure ⩾ 140 (90) mm Hg or use of antihypertensive medications	2491	Logistic regression	age-sex standardised: 29.9% (95% confidence interval (CI) 29.8–30.0). 29.4% (29.3–29.5) in men and 30.4% (30.4–30.6) in women. 75.8% of hypertensive participants were undetected before the survey, 34.9% of those aware of their hypertensive status were receiving treatment, of whom 16.3% were at target control levels	age, no formal education, obesity and diabetes were independently associated with hypertension. None of the interaction terms tested was significant.	All significant factors in univariable analysis (based on P o 0.10) were entered into a multivariable logistic regression model. Stepwise backward procedures were then used to retain the final predictors of hypertension (P o 0.05).
One-Year Rates and Determinants of Poststroke Systolic Blood Pressure Control among Ghanaians	retrospective observational study	Kumasi, Ghana (second largest city)	Stroke survivors,	59.9 ± 13.9 years	Stroke	Systolic Blood Pressure was deemed to be on target if clinic SBP was <140mmHg according to JNC-8 guidelines	602	multivariable logistic regression model was employed to assess the determinants of clinic SBP ≥140mmHg during follow-up	89.8% had hypertension	independent predictors of ≥ 1 SBP of ≥140mmHg post-enrollment on multivariable logistic regression analysis were systolic blood pressure at enrollment into clinic and the average number of anti-hypertensive medications prescribed over the course of follow-up with adjusted OR (95% CI) of 1.31 (1.17-1.47) and 1.30 (1.06-1.60) respectively	bivariable analysis, factors associated with the dependent variable at a p-value level of 0.10 were included in the multivariable model. In all analysis, two-tailed p-values <0.05 were considered statistically significant with no adjustments for multiple comparisons.
Prevalence, awareness, treatment and control of hypertension among adults in Kenya: cross-sectional national population-based survey	Prospective cross sectionalal study	Kenya	adults aged 18–69 years	38.0 (29–52)	No	Hypertensive: BP > 140/90 mm Hg or use of antihypertensive medication. Controlled: BP < 140/90 mmHg	4500	Multivariable logistic regression analysis.	28.6% of the population had hypertension, 29.2% among men and 27.9% among women, 29.4% were aware, 6.5% were currently using antihypertensive medication, and 12.5% [illegible]	older age, higher education, overweight and obesity, past month binge drinking, and type 2 diabetes were positive- ly associated with hypertension. In addition, underweight was negatively associated with hypertension.	Variables from bi- variate analysis with a significance level of P < 0.20 were included in the multivariable model. Multicollinearity among variables was checked by calculat- ing their variance inflation factor and none exceeded 2. P < 0.05 was considered significant
Evaluation of a community-based hypertension improvement program (ComHIP) in Ghana: data from a baseline survey	Retrospective cohort study	Ghana (Eastern Region)	Adults (>30)	49	No	○ Stage one hypertension: SBP ≥ 140 and < 160 mm/Hg, or DBP ≥90 and <100 mm/HG ○ Stage two hypertension: SBP ≥ 160- < 180 mm/Hg or DBP ≥100- <110 mm/HG ○ Stage three hypertension: SBP ≥ 180 mm/Hg or DBP ≥110 mm/Hg [2]	2337 (1170 comparison district, 1167 intervention)	Logistic regression	32.4% were hypertensive: 18.9% of them presented with stage one hypertension, 8.2% with stage two and 4.9% with stage three. 46.2% of the hypertensive individ- uals were aware of their condition	After adjusting for age, the risk factors with the greatest association with hypertension were being overweight (aOR = 2.30; 95% CI 1.53–3.46) or obese (aOR = 3.61; 95% CI 2.37–5.51). Older individuals were more likely to be aware of their hypertension status than younger people. After adjusting for age people with a family history of hypertension or CVD, or having an unhealthy waist hip ratio, were more likely to be aware of their hypertension status.	For each potential explana- tory variable we created a separate logistic regression model against the outcomes "hypertension status" and "hypertension awareness" separately. Each model was re- peated unadjusted by other variables and adjusted by age, sex and district. The models for the outcome "hypertension awareness" were restricted to people with hypertension only. P value less than 0.05 denotes statis- tical significance.
Urban rural differences in prevalence and risk factors of self-reported hypertension among Kenyan women: a population-based study	Retrospective, using Kenya Demographic and Health Survey (KDHS) data	Kenya (rural and urban)	Women (<49)	Not given	Excluded pregnant	self-reported hyper- tension	12,590 (urban 4726, rural 7864)	Multivariable logistic regression. Before entering the independent variables into the multivariable models, we checked the variation inflation factor (VIF) to avoid the problem of multi-collinearity. The VIF ranged from 1.05 to 2.59 for the rural and 1.02 to 2.49 for the urban sample.	overall prevalence of hypertension 9.38% (95% CI: 8.72, 10.08) higher among urban women 11.61% (95% CI: 10.46, 12.86), than rural women 7.86% (95% CI: 7.11, 8.68)	Overall, increasing age, overweight and obese, diabetes, and high levels of education were factors significantly associated with higher prevalence of hyper- tension in both rural and urban areas.	bivariate analysis (Chi-square) was performed to test the association of selected categorical variables with the dependent variable. An arbitrary p value of <0.20 was used as criteria to include the variables in the multivariable logistic regression model to control for con- founding effects, and the results were considered statisti- cally significant at p value of ≤0.05 for both rural and urban sample.

Table S5. PRISMA checklist

Section and Topic	Item #	Checklist item	Location where item is reported
**TITLE**			
Title	1	Identify the report as a systematic review.	1
**ABSTRACT**			
Abstract	2	See the PRISMA 2020 for Abstracts checklist.	2
**INTRODUCTION**			
Rationale	3	Describe the rationale for the review in the context of existing knowledge.	3
Objectives	4	Provide an explicit statement of the objective(s) or question(s) the review addresses.	3
**METHODS**			
Eligibility criteria	5	Specify the inclusion and exclusion criteria for the review and how studies were grouped for the syntheses.	3
Information sources	6	Specify all databases, registers, websites, organisations, reference lists and other sources searched or consulted to identify studies. Specify the date when each source was last searched or consulted.	3
Search strategy	7	Present the full search strategies for all databases, registers and websites, including any filters and limits used.	Supplementary
Selection process	8	Specify the methods used to decide whether a study met the inclusion criteria of the review, including how many reviewers screened each record and each report retrieved, whether they worked independently, and if applicable, details of automation tools used in the process.	3
Data collection process	9	Specify the methods used to collect data from reports, including how many reviewers collected data from each report, whether they worked independently, any processes for obtaining or confirming data from study investigators, and if applicable, details of automation tools used in the process.	4
Data items	10a	List and define all outcomes for which data were sought. Specify whether all results that were compatible with each outcome domain in each study were sought (e.g. for all measures, time points, analyses), and if not, the methods used to decide which results to collect.	4
	10b	List and define all other variables for which data were sought (e.g. participant and intervention characteristics, funding sources). Describe any assumptions made about any missing or unclear information.	4
Study risk of bias assessment	11	Specify the methods used to assess risk of bias in the included studies, including details of the tool(s) used, how many reviewers assessed each study and whether they worked independently, and if applicable, details of automation tools used in the process.	4
Effect measures	12	Specify for each outcome the effect measure(s) (e.g. risk ratio, mean difference) used in the synthesis or presentation of results.	4
Synthesis methods	13a	Describe the processes used to decide which studies were eligible for each synthesis (e.g. tabulating the study intervention characteristics and comparing against the planned groups for each synthesis (item #5)).	4
	13b	Describe any methods required to prepare the data for presentation or synthesis, such as handling of missing summary statistics, or data conversions.	4
	13c	Describe any methods used to tabulate or visually display results of individual studies and syntheses.	4
	13d	Describe any methods used to synthesize results and provide a rationale for the choice(s). If meta-analysis was performed, describe the	4

For peer review only

Section and Topic	Item #	Checklist item	Location where item is reported
		model(s), method(s) to identify the presence and extent of statistical heterogeneity, and software package(s) used.	
	13e	Describe any methods used to explore possible causes of heterogeneity among study results (e.g. subgroup analysis, meta-regression).	N/A
	13f	Describe any sensitivity analyses conducted to assess robustness of the synthesized results.	N/A
Reporting bias assessment	14	Describe any methods used to assess risk of bias due to missing results in a synthesis (arising from reporting biases).	N/A
Certainty assessment	15	Describe any methods used to assess certainty (or confidence) in the body of evidence for an outcome.	N/A
**RESULTS**			
Study selection	16a	Describe the results of the search and selection process, from the number of records identified in the search to the number of studies included in the review, ideally using a flow diagram.	4
	16b	Cite studies that might appear to meet the inclusion criteria, but which were excluded, and explain why they were excluded.	Supplementary
Study characteristics	17	Cite each included study and present its characteristics.	Table 1
Risk of bias in studies	18	Present assessments of risk of bias for each included study.	7 and Supplementary
Results of individual studies	19	For all outcomes, present, for each study: (a) summary statistics for each group (where appropriate) and (b) an effect estimate and its precision (e.g. confidence/credible interval), ideally using structured tables or plots.	8
Results of syntheses	20a	For each synthesis, briefly summarise the characteristics and risk of bias among contributing studies.	8
	20b	Present results of all statistical syntheses conducted. If meta-analysis was done, present for each the summary estimate and its precision (e.g. confidence/credible interval) and measures of statistical heterogeneity. If comparing groups, describe the direction of the effect.	8
	20c	Present results of all investigations of possible causes of heterogeneity among study results.	N/A
	20d	Present results of all sensitivity analyses conducted to assess the robustness of the synthesized results.	N/A
Reporting biases	21	Present assessments of risk of bias due to missing results (arising from reporting biases) for each synthesis assessed.	N/A
Certainty of evidence	22	Present assessments of certainty (or confidence) in the body of evidence for each outcome assessed.	N/A
**DISCUSSION**			
Discussion	23a	Provide a general interpretation of the results in the context of other evidence.	8
	23b	Discuss any limitations of the evidence included in the review.	8
	23c	Discuss any limitations of the review processes used.	9
	23d	Discuss implications of the results for practice, policy, and future research.	9

For peer review only

Section and Topic	Item #	Checklist item	Location where item is reported
**OTHER INFORMATION**			
Registration and protocol	24a	Provide registration information for the review, including register name and registration number, or state that the review was not registered.	3
	24b	Indicate where the review protocol can be accessed, or state that a protocol was not prepared.	3
	24c	Describe and explain any amendments to information provided at registration or in the protocol.	3
Support	25	Describe sources of financial or non-financial support for the review, and the role of the funders or sponsors in the review.	1, 4, 9
Competing interests	26	Declare any competing interests of review authors.	9
Availability of data, code and other materials	27	Report which of the following are publicly available and where they can be found: template data collection forms; data extracted from included studies; data used for all analyses; analytic code; any other materials used in the review.	N/A

For peer review only

Section and Topic	Item #	Checklist item	Location where item is reported
**TITLE**			
Title	1	Identify the report as a systematic review.	1
**ABSTRACT**			
Abstract	2	See the PRISMA 2020 for Abstracts checklist.	2
**INTRODUCTION**			
Rationale	3	Describe the rationale for the review in the context of existing knowledge.	3
Objectives	4	Provide an explicit statement of the objective(s) or question(s) the review addresses.	3
**METHODS**			
Eligibility criteria	5	Specify the inclusion and exclusion criteria for the review and how studies were grouped for the syntheses.	3
Information sources	6	Specify all databases, registers, websites, organisations, reference lists and other sources searched or consulted to identify studies. Specify the date when each source was last searched or consulted.	3
Search strategy	7	Present the full search strategies for all databases, registers and websites, including any filters and limits used.	Supplementary
Selection process	8	Specify the methods used to decide whether a study met the inclusion criteria of the review, including how many reviewers screened each record and each report retrieved, whether they worked independently, and if applicable, details of automation tools used in the process.	3
Data collection process	9	Specify the methods used to collect data from reports, including how many reviewers collected data from each report, whether they worked independently, any processes for obtaining or confirming data from study investigators, and if applicable, details of automation tools used in the process.	4
Data items	10a	List and define all outcomes for which data were sought. Specify whether all results that were compatible with each outcome domain in each study were sought (e.g. for all measures, time points, analyses), and if not, the methods used to decide which results to collect.	4
	10b	List and define all other variables for which data were sought (e.g. participant and intervention characteristics, funding sources). Describe any assumptions made about any missing or unclear information.	4
Study risk of bias assessment	11	Specify the methods used to assess risk of bias in the included studies, including details of the tool(s) used, how many reviewers assessed each study and whether they worked independently, and if applicable, details of automation tools used in the process.	4
Effect measures	12	Specify for each outcome the effect measure(s) (e.g. risk ratio, mean difference) used in the synthesis or presentation of results.	4
Synthesis methods	13a	Describe the processes used to decide which studies were eligible for each synthesis (e.g. tabulating the study intervention characteristics and comparing against the planned groups for each synthesis (item #5)).	4
	13b	Describe any methods required to prepare the data for presentation or synthesis, such as handling of missing summary statistics, or data conversions.	4
	13c	Describe any methods used to tabulate or visually display results of individual studies and syntheses.	4
	13d	Describe any methods used to synthesize results and provide a rationale for the choice(s). If meta-analysis was performed, describe the model(s), method(s) to identify the presence and extent of statistical heterogeneity, and software package(s) used.	4

Section and Topic	Item #	Checklist item	Location where item is reported
	13e	Describe any methods used to explore possible causes of heterogeneity among study results (e.g. subgroup analysis, meta-regression).	N/A
	13f	Describe any sensitivity analyses conducted to assess robustness of the synthesized results.	N/A
Reporting bias assessment	14	Describe any methods used to assess risk of bias due to missing results in a synthesis (arising from reporting biases).	N/A
Certainty assessment	15	Describe any methods used to assess certainty (or confidence) in the body of evidence for an outcome.	N/A
**RESULTS**			
Study selection	16a	Describe the results of the search and selection process, from the number of records identified in the search to the number of studies included in the review, ideally using a flow diagram.	4
	16b	Cite studies that might appear to meet the inclusion criteria, but which were excluded, and explain why they were excluded.	Supplementary
Study characteristics	17	Cite each included study and present its characteristics.	Table 1
Risk of bias in studies	18	Present assessments of risk of bias for each included study.	7 and Supplementary
Results of individual studies	19	For all outcomes, present, for each study: (a) summary statistics for each group (where appropriate) and (b) an effect estimate and its precision (e.g. confidence/credible interval), ideally using structured tables or plots.	8
Results of syntheses	20a	For each synthesis, briefly summarise the characteristics and risk of bias among contributing studies.	8
	20b	Present results of all statistical syntheses conducted. If meta-analysis was done, present for each the summary estimate and its precision (e.g. confidence/credible interval) and measures of statistical heterogeneity. If comparing groups, describe the direction of the effect.	8
	20c	Present results of all investigations of possible causes of heterogeneity among study results.	N/A
	20d	Present results of all sensitivity analyses conducted to assess the robustness of the synthesized results.	N/A
Reporting biases	21	Present assessments of risk of bias due to missing results (arising from reporting biases) for each synthesis assessed.	N/A
Certainty of evidence	22	Present assessments of certainty (or confidence) in the body of evidence for each outcome assessed.	N/A
**DISCUSSION**			
Discussion	23a	Provide a general interpretation of the results in the context of other evidence.	8
	23b	Discuss any limitations of the evidence included in the review.	8
	23c	Discuss any limitations of the review processes used.	9
	23d	Discuss implications of the results for practice, policy, and future research.	9
**OTHER INFORMATION**			

For peer review only

Section and Topic	Item #	Checklist item	Location where item is reported
Registration and protocol	24a	Provide registration information for the review, including register name and registration number, or state that the review was not registered.	3
	24b	Indicate where the review protocol can be accessed, or state that a protocol was not prepared.	3
	24c	Describe and explain any amendments to information provided at registration or in the protocol.	3
Support	25	Describe sources of financial or non-financial support for the review, and the role of the funders or sponsors in the review.	1, 4, 9
Competing interests	26	Declare any competing interests of review authors.	9
Availability of data, code and other materials	27	Report which of the following are publicly available and where they can be found: template data collection forms; data extracted from included studies; data used for all analyses; analytic code; any other materials used in the review.	N/A

For peer review only

BMJ Open

Use of technology to prevent, detect, manage, and control hypertension in sub-Saharan Africa: a systematic review

Journal:	*BMJ Open*
Manuscript ID	bmjopen-2021-058840.R1
Article Type:	Original research
Date Submitted by the Author:	19-Jan-2022
Complete List of Authors:	Stokes, Katherine; University of Warwick,<br>Oronti, Busola; University of Warwick<br>Cappuccio, Francesco Paolo; University of Warwick, Warwick Medical School, Division of Health Sciences<br>Pecchia, Leandro ; University of Warwick,
**Primary Subject Heading**:	Cardiovascular medicine
Secondary Subject Heading:	Cardiovascular medicine, Global health
Keywords:	Hypertension < CARDIOLOGY, Telemedicine < BIOTECHNOLOGY & BIOINFORMATICS, Information technology < BIOTECHNOLOGY & BIOINFORMATICS

SCHOLARONE™
Manuscripts

I, the Submitting Author has the right to grant and does grant on behalf of all authors of the Work (as defined in the below author licence), an exclusive licence and/or a non-exclusive licence for contributions from authors who are: i) UK Crown employees; ii) where BMJ has agreed a CC-BY licence shall apply, and/or iii) in accordance with the terms applicable for US Federal Government officers or employees acting as part of their official duties; on a worldwide, perpetual, irrevocable, royalty-free basis to BMJ Publishing Group Ltd ("BMJ") its licensees and where the relevant Journal is co-owned by BMJ to the co-owners of the Journal, to publish the Work in this journal and any other BMJ products and to exploit all rights, as set out in our licence.

The Submitting Author accepts and understands that any supply made under these terms is made by BMJ to the Submitting Author unless you are acting as an employee on behalf of your employer or a postgraduate student of an affiliated institution which is paying any applicable article publishing charge ("APC") for Open Access articles. Where the Submitting Author wishes to make the Work available on an Open Access basis (and intends to pay the relevant APC), the terms of reuse of such Open Access shall be governed by a Creative Commons licence – details of these licences and which Creative Commons licence will apply to this Work are set out in our licence referred to above.

Other than as permitted in any relevant BMJ Author's Self Archiving Policies, I confirm this Work has not been accepted for publication elsewhere, is not being considered for publication elsewhere and does not duplicate material already published. I confirm all authors consent to publication of this Work and authorise the granting of this licence.

For peer review only

Title	**Use of technology to prevent, detect, manage, and control hypertension in sub-Saharan Africa: a systematic review**
Authors' names	Katy Stokes[1*], Busola Oronti[1], Francesco P Cappuccio[2], Leandro Pecchia[1]
Address for each author	1. School of Engineering, University of Warwick, Coventry, UK<br>2. Medical School, University of Warwick, Coventry, UK
Authors' names and positions	Katy Stokes<br>PhD student<br>Busola Oronti<br>PhD student<br>Francesco P Cappuccio<br>Professor<br>Leandro Pecchia<br>Professor
Corresponding author	*Correspondence to: Katy.Stokes@warwick.ac.uk

For peer review only

For peer review only - http://bmjopen.bmj.com/site/about/guidelines.xhtml

Abstract

Objective To identify and assess the use of technologies, including mobile health technology, internet of things devices and artificial intelligence in hypertension healthcare in sub-Saharan Africa (SSA).

Design Systematic review.

Data sources Medline, Embase, Scopus and Web of Science.

Eligibility criteria Studies addressing outcomes related to the use of technologies for hypertension healthcare (all points in the healthcare cascade) in SSA.

Methods Databases were searched from inception to Aug 2, 2021. Screening, data extraction and risk of bias assessment were done in duplicate. Data was extracted on study design, setting, technology(s) employed and outcomes. Blood pressure (BP) reduction due to intervention was extracted from a subset of randomised controlled trials. Methodological quality was assessed using the Mixed Methods Appraisal Tool (MMAT).

Results 1717 hits were retrieved, 1206 de-duplicated studies were screened, and 67 full texts were assessed for eligibility. 22 studies were included, all reported on clinical investigations. Two studies were observational, and 20 evaluated technology-based interventions. Outcomes included BP reduction/control, treatment adherence, retention in care, awareness/knowledge of hypertension and completeness of medical records. All studies utilised mobile technology, three linked with IoT devices. SMS was the most popular method of targeting patients (n=6). Moderate BP reduction was achieved in three randomised controlled trials. Patients and healthcare providers reported positive perceptions towards the technologies. No studies using AI were identified.

Conclusions There are a range of successful applications of key enabling technologies in SSA, including BP reduction, increased health knowledge and treatment adherence following targeted mobile technology interventions. There is evidence to support use of mobile technology for hypertension management in SSA. However, current application of technologies is highly heterogenous and key barriers exist, limiting efficacy and uptake in SSA. More research is needed, addressing objective measures such as BP reduction in robust randomised studies.

Registration PROSPERO CRD42020223043.

Funding KS is funded by the Medical Research Council Doctoral Training Partnership [grant number MR/N014294/1].

Strengths and limitations of this study

- This is the first systematic review for use of technologies for hypertension healthcare in sub-Saharan Africa, providing a comprehensive review of the state of the art.
- Heterogeneity of included studies was too high for meta-analysis; therefore, results are reported narratively.
- Grey literature was not searched.
- The search was limited to studies published in English language.

For peer review only

Introduction

Cardiovascular disease (CVD) remains the most common cause of death due to non-communicable disease (NCD) worldwide, with 78% of deaths occurring in low- and middle-income countries (LMICs).[1 2] Hypertension (high blood pressure (BP)) is considered by the World Health Organisation (WHO) to be the leading risk factor for developing CVD[1] and by the Pan-African Society of Cardiology (PASCAR) as the highest priority area for reducing heart disease and stroke in Africa.[3] In sub-Saharan Africa (SSA), the prevalence of hypertension is high, especially in younger subjects, estimated at 46% of the adult population, in contrast with 35% in high-income countries.[3] Reasons proposed for this include urbanization, increase in life expectancy and lifestyle factors such as poor diet, physical inactivity and smoking.[4] A meta-analysis performed by Atakite et al. in 2011 reports that of those with hypertension in SSA, only 27% were aware of their condition, 18% were receiving treatment and 7% had controlled blood pressure.[5]

Low numbers of trained healthcare providers combined with a lack of evidence-based guidance and a high-cost in accessing healthcare services for patients in SSA are major challenges.[6] Cost-effective technologies will likely play a critical role to overcome such barriers, through decision support tools;[7 8] dissemination of health information including education and treatment reminders[9 10] and collection and storage of medical data.[11 12] Indeed, the value of information and communication technologies (ICT) to health services has been recognised by the WHO for over ten years.[13] eHealth is defined by the WHO as "the cost-effective and secure use of ICT in support of health and health-related fields, including health-care services, health surveillance, health literacy, and health education, knowledge and research".[14] In this way, eHealth can be delivered through several Key Enabling Technologies (KETs): mobile phone technology, Artificial Intelligence (AI) and the Internet of Things (IoT). Mobile phone use is high in SSA, 45% of the population subscribe to mobile services and this use is projected to increase.[15] Research interests into use of mobile phones for healthcare purposes in SSA concern either infectious disease or maternal and child health,[16-18] but attention to NCD is growing.[19 20] AI has many possible definitions, in essence describing a motivation to replicate and automate human cognitive functions, having a myriad of healthcare applications[21] which have been exploited in high-income countries. Although research in LMICs is relatively limited,[22] drivers such as high disease burden, few qualified health care workers and increasing phone and internet connection may drive a rapid advance in AI for healthcare in LMICs.[23] Wahl et al. describe uses of AI in resource-poor settings, including expert systems assisting or compensating for a lack of personnel, health monitoring using natural language processing and signal processing for diagnostics.[22]

Successful application of the afore mentioned technologies for tackling hypertension relies on a strong evidence base in design and implementation. In this way, this work seeks to systematically review the literature regarding the application of mobile phone technologies, AI and the IoT as KETs for healthcare provision for hypertension in SSA. The primary objective is to determine how and which KETs have been used, secondary concerns include study design, setting, quality and findings of outcomes relating to hypertension.

Methods

Search strategy and selection criteria

The systematic review of KETs for healthcare provision for hypertension in SSA followed Preferred Reporting Items for Systematic Reviews and Meta-Analyses (PRISMA) 2020 guidelines.[24] We searched Embase, MEDLINE, Web of Science and Scopus electronic databases for studies published in English language only. The search was run from database inception to Nov 23rd, 2020 and updated Aug 2nd, 2021.

Search terms covered hypertension (e.g., "hypertension", "high blood pressure"), Artificial Intelligence (e.g., "AI", "machine learning"), mobile phones (e.g., "mobile phon*", "mobile"), internet of things (e.g., "internet of things", "iot"), point of healthcare cascade (e.g., "prevention", "screening") and countries of SSA (full strings in Supplementary S1).

Studies seeking to assess the application of KETs in SSA, for any point in the healthcare cascade for hypertension, were considered for inclusion. There were no restrictions set on study methodology in terms of participant recruitment, age, or comorbidity. Studies were required to be conducted using populations from SSA countries, or from a pool of countries including at least one SSA country. For inclusion, studies must have provided an evaluation of the use of KETs for any aspect of healthcare for hypertension or used AI models for predicting or detecting significant events. Studies which focused only on prevalence or risk factors, i.e., used statistical methods but did not develop AI based predictive models, were considered out of the scope of this review. The study protocol was registered with PROSPERO: International Prospective Register of Systematic Reviews and is found at: http://www.crd.york.ac.uk/prospero – registration number CRD42020223043. Ethical approval was not required.

For peer review only - http://bmjopen.bmj.com/site/about/guidelines.xhtml

Data analysis

Screening was completed independently by two authors (KS and BO). A reference search was conducted on any relevant review articles retrieved. For included studies, data were abstracted to a shared Microsoft Excel document, covering study design, study setting and population (age, demographics, comorbidity), details of KET used, study outcomes, controls/comparators (where applicable), target user (where applicable) and indications of acceptability to user (if provided). For randomised controlled trials, we sought to extract mean baseline and endpoint blood pressure measurements (in mm Hg), with standard deviation, for the intervention and control groups. If standard deviation was not reported, it was calculated using the confidence interval, as described in the Cochrane Handbook for Systematic Reviews of Interventions.[25] In the event that participants were lost to follow-up, the final number of participants that completed the study protocol was extracted. As heterogeneity among studies was high (no two studies evaluated the same intervention), we used a random-effects model to establish the effect of KET based interventions on systolic blood pressure reduction. We did the analysis in Open Meta-Analyst,[26] an open-source, cross-platform software for meta-analysis.

Methodological quality was assessed independently by two review authors (KS and BO) using the 2018 Mixed Methods Appraisal Tool (MMAT)[27] for assessing the quality of either quantitative, qualitative, or mixed methods studies. Criteria were graded as 'unmet', 'met' or 'can't tell'. For mixed methods studies, provided most criteria were met (three or more out of five) for each component, the components were considered to have adhered to their respective quality criteria (criterion 5.5).

Role of funding source

The funder of the study had no role in study design, data collection, data analysis, data interpretation, or writing of the report.

Patient and Public Involvement

Patients and the public were not involved in this research.

Research Ethics Approval

Ethical approval was not required.

Results

Searching MEDLINE, Scopus, Embase and Web of Science yielded 1717 titles. After duplicate removal, 1206 titles/abstracts were screened, with 1139 excluded. Of the remaining 67 full-texts, 18 studies were found to meet the inclusion criteria (**figure 1**). A further four studies were identified through a linear search of the bibliographies of relevant reviews identified during the initial screening. **Table 1** contains a summary of the characteristics of the included studies.

Figure 1. Study selection

Table 1. Characteristics of included studies

	Study location	Population (age (sd))	Duration	Sample size
**Kingue et al. 2013[28]**	Yaounde, capital city of Cameroon and rural health districts (within 50-250km), Telemedicine centre based at Yaounde General Hospital	Age >15, with hypertension not at target level (SBP (or DBP) >=140 (90) mmHg or >=130 (80) mmHg (for those with diabetes or nephropathy). (Control: 57.6 (12.1), Intervention: 59.9 (10.4))	24 weeks	30 healthcare centres (10 intervention, 20 control). Total: 268 participants (A: Intervention n=165, B: Control n=103)
**Ola-Olorun et al., 2014[29]**	Nigeria, (Outpatient clinic Obafemi Awolowo University Teaching Hospital)	Long term hypertension patients		Total: 187 participants (exposed to SMS, n=111)
**Joubert et al., 2014[30]**	Botswana (suburb)	Adults (>18) (39 (16))	NA	Total: 92 participants
**Leon et al. 2015[31] (STAR)**	South Africa, Cape Town, Primary care facility of a large public sector clinic,	A diverse sample of population of Bobrow et al. 2016 [33]	NA	22 trial participants took part in 2 focus groups, 15 individual in-depth interviews
**Vedanthan et al., 2015[32]**	Kenya (rural)	Nurses, clinical officer	NA	Total: 13 participants (12 nurses, 1 clinical officer)

For peer review only - http://bmjopen.bmj.com/site/about/guidelines.xhtml

**Bobrow et al. 2016[33] (STAR)**	As above, Leon et al. 2015[31]	Adults (>=21) with access and ability to use a mobile phone for SMS; diagnosed with hypertension; prescribed blood pressure lowering medication; and with SBP <220 mm Hg and a <120 mm Hg at enrolment. (Usual care: 54.7 (11.6), Information only: 53.9 (11.2), Interactive: 54.2 (11.6))	12 months	Total: 1372 participants (A: information-only SMS text-messages n=457, B: interactive SMS text-messages n=458, C: usual care n=457)
**Hacking et al. 2016[34]**	South Africa: Gugulethu township of Cape Town (densely populated, poor urban settlement)	Patients of hypertension clinic. (52.83 (11.62))	17 weeks	Total: 223 participants, (Intervention n=109, Control n=114)
**Haricharan et al., 2017[35]**	South Africa, Cape Town	Convenience sample	28 weeks	Total: 41 participants
**Kleczka et al., 2018[36]**	Kenya, Nairobi Health Centre	Patient charts classified with hypertension	6 months	Total: 70 patients' charts (291 clinical encounters for HTN across 49 patients (149 pre-intervention and 142 post-intervention))
**Mannik et al., 2018[37] (AFYACHAT)**	Kenya (rural), Two rural primary health clinics: Isiolo District, Marakwet District	Adults (>40 years) (50 (43–60))	22 months	Total: 2865 participants
**Nelissen et al., 2018[38]**	Nigeria (Lagos)	Hypertensive adults (54.9 (11.9))	6-8 months	Total: 336 participants
**Sarfo et al., 2018[39] (PINGS)**	Ghana, Outpatient Neurology clinic (Komfo Anokye Teaching Hospital KATH)	Adults, >18, recently confirmed stroke (<1month) by CT, with uncontrolled hypertension (SBP≥140 mmHg) (55 (13))	3 months / 9 months	Total: 60 participants (Intervention n=30, Control n=30)
**Yedanthan et al. 2019[40]**	Western Kenya: rural healthcare facilities in Kosirai and Turbo divisions	Adults, with elevated BP (SBP ≥ 140 or DBP ≥ 90). (60.8 (14.2))	15 months	Total: 1460 participants (A: usual care n=491, B: paper-based n=500, C: smartphone n=469)
**Owolabi et al. 2019[41] (THRIVES)**	Nigeria, A range of facilities chosen to represent the diverse South-western population and hospital types	Adults >=18 with access to a mobile phone, recently discharged from hospital following a stroke. (57.2 (SD 11.7))	12 months	Total: 400 participants (Intervention n=200, Control n=200)
**Sarfo et al., 2019[42] (PINGS)**	As above, Sarfo et al., 2018[39]	Adults, >18, recently confirmed stroke (<1month) by CT, with uncontrolled hypertension (SBP≥140 mmHg)	9 months	Total: 60 participants (Intervention n=30, Control n=30)
**Nichols et al., 2019[43] (PINGS)**	As above, Sarfo et al., 2018[39]			24 patients, 8 caregivers, 7 research team
**Cremers et al., 2019[44]**	As above, Nelissen et al., 2018 [38]	As above, Nelissen et al., 2018 [38]	NA	In-depth interviews total: 30 patients (9 community pharmacists, 6 cardiologists) Structured interviews total: 328 patients
**Barsky et al., 2019[45]**	Tanzania (rural)	Adults (>=18) with uncontrolled hypertension. Either own mobile or be willing to take one	10 months	Total: 130 participants
**Oduor et al., 2019[46]**	Kenya (rural)	Adults with HIV and hypertension	NA	Total: 36 participants (27 medical practitioners, 9 patients)
**Adler et al., 2020[47]**	Ghana, Lower Manya-Krobo District (84% urban population)	Patients, nurses, clinicians, physician's assistant, pharmacist		Total: 55 participants (15 patients, 7 nurses, 1 clinician, 1 physician assistant, 1 pharmacist)
**Yedanthan et al., 2020[48]**	As above, Vedanthan et al., 2015 [32]	Adults (>35) Confirmed diagnosis of hypertension (61 (13.2))	3 months	Total: 1051 participants (180 under care of nurse, 871 under care of clinical officer)
**Aw et al., 2020[49] (AFYACHAT)**	As above, Mannik et al., 2018[37]	Adults (>40 years) (50 (43–59))	5-8 months	Total: 1650 participants

Study design, participants, location, and aims were highly heterogenous (**table 1**). The distribution of studies by country is shown in **figure 2**. All included articles reported on clinical investigations.[28-49] Two were observational studies [30 46] and 20 concerned evaluation of KET based interventions.[28 29 31-45 47-49] Both observational studies utilised mixed methods to explore either current use of technology for hypertension management or hypertension prevalence, understanding and awareness. Several of the interventional studies fell within the same larger study, granting fourteen unique experimental studies. Separate articles within these studies reported on different aspects such as impact of intervention, feasibility, and perceptions. Eight studies were randomised controlled trials.[28 31 33 34 39-43 45 47] Quantitative primary outcomes were BP reduction and BP control. Other outcomes included treatment adherence, retention in care, awareness/knowledge of hypertension and completeness of medical records. Length of exposure was highly heterogeneous, ranging from 17 weeks to two years.

Quality was highest for qualitative and quantitative descriptive methods and relatively low for randomised or non-randomised studies (Supplementary Table S2). 11 of 13 studies comprising qualitative components satisfied all criteria,[29 31-35 38 43 46 47 49] whereas insufficient reporting of results in two studies meant it

For peer review only

For peer review only - http://bmjopen.bmj.com/site/about/guidelines.xhtml

was not possible to determine if findings had been adequately derived or substantiated from the data.[37 45] In terms of randomised controlled trials, four met all quality criteria[33 39 41 42], three did not use appropriate randomisation methods[28 34 40] and three did not report complete outcome data.[34 40 45] Notably, Barsky et al. did not report sufficient information to judge four out of five criteria[45] and Vedanthan et al. failed to meet any criteria.[40] Lack of complete outcome data was also an issue in four out of six non-randomised studies.[35 38 44 48] Three of four quantitative descriptive studies met all quality criteria[30 37 42] with one study subject to voluntary selection bias, which was discussed by the authors.[49]

All studies employed adult populations, with varying age requirements (table 1). Five studies used subjects or data collected in a predominantly urban setting,[31 34-36 38], six in rural locations,[28 32 37 40 45 46] two in both[30 41] and one study did not provide specific location or population demographic details.[29] The majority of the experimental research recruited subjects with elevated BP[40] or confirmed hypertension/prescribed anti-hypertensives,[28 29 31 32 34 36 38 39 47] with the aim of improving hypertension control, treatment adherence or health knowledge, otherwise, the aim of the study was to test a health tool for identifying hypertension or general CVD risk factors within a certain population.[37] In terms of comorbidity or other conditions, one study focused on patients having recently suffered stroke (with or without hypertension),[41] one focused on diabetic patients,[28] one concerned HIV positive hypertensive subjects[46] and one employed a convenience sample of a Deaf community.[35] In three cases, participants were required to have access to a mobile phone for inclusion in the study.[31 41 47]

Figure 2: Distribution of studies across sub-Saharan Africa.
Countries are coloured based on the number of studies conducted (darker indicates more studies) and annotated with frequency (where a large study had several associated publications, the location is reported once).

Table 2 describes the different applications of technologies and their frequency of use. All studies utilised mobile phone technology, including SMS, phone calls and mobile applications (apps), either alone or in combination. Odour et al. evaluated the general use and perceptions of medical practitioners and patients towards technology, in particular mobile technology, in dealing with hypertension and HIV. IoT devices employed were automatic BP monitors and their use was also facilitated by mobile phones.

Table 2. Summary of KETs utilised in study pool

	SMS (11)	Smartphone/ Tablet with App (8)	Mobile/Smartphone/ Tablet without App (6)	IoT (3)	Web-based Data Storage and Tools (7)	Description of technology	User
StAR: SMS-text Adherence Support[31 33] †*	+		+			SMS sent to patients to elicit behavioural changes, focusing on providing educational and motivational messages about hypertension and its treatment	Patient
LARK: Linkage And Retention to hypertension care in rural Kenya, Vedanthan et al. 2019[40] †*		+			+	Smartphone linked to electronic health record: Provides CHW with automatically updated list of patients requiring follow-up and real-time decision support using clinically approved care algorithms	CHW
Kingue et al. 2013[28] †*	+		+			Mobile phone communication: Links with telemedicine centre via SMS, voicemail, and phone calls. Real-time feedback to aid decision making.	Healthcare provider
Owolabi et al. 2019[41] †*	+				+	SMS messages for appointment reminders and self-management support.	Patient and care provider
Hacking et al. 2016[34] †*	+					SMS messages containing information on hypertension and healthy lifestyle suggestions.	Patient
PINGS: Phone-based Intervention under Nurse Guidance after Stroke[39 42 43] †*	+	+		+	+	BP reading device, connects via blue tooth and smart phone given to patients, stores and reports BP measurements and medication intake. Also, motivational SMS based on adherence to medication.	Patient
ComHIP: community-based hypertension management project[47] †*	+		+	+	+	Telemedicine consultation by CVD nurse with physician in order to refer serious hypertension on, ICT messages for healthy lifestyles, treatment adherence support and treatment refill reminders, Cloud based EMR system linked with SMS/voice messaging for treatment adherence, reminders and	Patient and care provider

For peer review only - http://bmjopen.bmj.com/site/about/guidelines.xhtml

						health messaging, OMRON digital sphygmomanometer	
DESIRE: Decision-Support and Integrated Record-keeping[32 38 48] †		+				Tablet-based Decision Support and Integrated Record- keeping	Nurses/ clinical officers
Pharmacy task shift[44] †		+				mHealth mobile application to facilitate communication between pharmacists and cardiologists	Pharmacists and remote cardiologists
AFYACHAT: health chat[37 49] †	+	+			+	mHealth mobile application for data collection including an algorithmic risk stratification based on WHO guidelines	CHWs
Ola-Olorun et al., 2014[29] †	+					SMS messaging to connect patient to pharmacist and also to deliver reminders for medication and clinic appointments to patients	Patient and pharmacist
Kleczka et al., 2018[36] †			+		+	Digital data extraction and management, including guidelines for specific diseases to be stamped, filled and digitised using mobile phones	Clinical staff
Haricharan et al., 2017[35] †	+					SMS containing information on hypertension (e.g., symptoms, consequences) and tips for healthy living (e.g., eating habits, exercise)	Patient (public, Deaf)
Barsky et al., 2019[45] †*	+	+		+	+	Bluetooth-enabled blood pressure monitor, linked to a mobile phone with DREAM-GLOBAL app to collect readings. Central server assessed readings as normal or high. SMS directed to patient to prompt seeking healthcare	CHW, patient
Oduor et al., 2019[46]	+	+	+			Any reported by participants	Patients and care providers
Joubert et al., 2014[30]		+	+			Tablet computer used to collect survey data and transmit via tele-contact	Clinical staff

† Interventional studies
* Randomised control trials

SMS messages were mostly targeted to patients, for health knowledge improvement,[34 35] motivation/improved treatment adherence[29 39 41] or both.[31] Content included reminders for taking medication or attending clinics/appointments, educational information covering general healthy lifestyle suggestions (e.g., eating habits, exercise), or hypertension information (e.g., symptoms, further health consequences, medication information). SMS were also used in combination with other elements in broader interventions to facilitate decision support for healthcare providers (e.g., through direct feedback of risk stratification)[28 37] or to provide an interactive connection between patients and pharmacists[29] or specialist telehealth connection (e.g., direct feedback from cardiologist to pharmacists).[28 44]

Apps were targeted to health care providers, most frequently community health workers (CHWs). Three studies utilised apps designed to mediate BP reading collection and dissemination of results for risk assessment and follow-up.[42 45 49] The DREAM-GLOBAL app received BP readings from an automatic monitor via Bluetooth (UA-767 Plus BT), relayed the readings to a remote central server, which then calculated the average reading and transmitted the patients results to their primary health care provider by fax, and to the patient themselves by SMS.[45] For high BP readings, the patient was notified to seek advice with their health care provider. Similarly, in the Phone-based Intervention under Nurse Guidance after Stroke (PINGS) study, the same Bluetooth BP monitor was linked with an app for monitoring and reporting measurements as well as medication intake. Participants monitored their own BP, following training from a study nurse. Levels of medication intake were monitored, and tailored motivational SMS were delivered to participants based on these results.[39] The AFYACHAT mobile app functioned in a similar way, BP readings were entered by the CHW, along with other patient data, the app then provided an algorithmic risk stratification (based on WHO's Prevention of CVD: Pocket Guidelines for Assessment and Management of Cardiovascular Risk, 2007) via SMS.[37 49] The Decision-Support and Integrated Record-Keeping (DESIRE) tool, an app designed for nurses to use on tablets, also provided clinical decision support, here through the AMPATH hypertension management algorithm, which is based on WHO clinical algorithms.[32] The DESIRE tool included functions for data entry and validation, decision support, alerts, and reminders, and viewing historical data on. Other authors discussed the feasibility of mobile technology facilitated

For peer review only

screening for hypertension. Joubert et al. collected survey data using a tablet computer to collect and relay patient information to a central database.[30] Another focus of research was digitisation and storage of patient data from previous paper-based systems. Kleczkaa et al. described use of rubber stamp templates containing checklists of clinical practice guidelines; smartphone cameras were used to take images of these templates which were then manually synched to a cloud-based database, with plans for further automation.[36] A cloud-based health records system was also utilised as part of the ComHIP hypertension improvement project, which facilitated delivery of SMS and aimed to allow all levels of health providers access to patients' records.

Concerning outcomes relating either to reduction of BP or improved BP control, the majority of experimental studies reported that their interventions resulted in improvements.[28 33 38 40 42] However, analysis by Nelissen et al.[38] found that the mHealth app element of their intervention was not associated with the observed BP improvements, based on duration of patient activity measured by the app.[38] Four studies detected no difference[41] or statistically insignificant changes[33 39 40] between control and intervention groups. Vedanthan et al. observed significant reduction in both systolic and diastolic BP regardless of whether their tablet-based decision support tool was used by nurses or clinical officers but did not have a control group. Heterogeneity in both outcomes investigated and reported prevented quantitative comparison. Results from three randomised controlled trials which reported baseline and endpoint values for systolic BP are presented in **figure 3**. It should be emphasised that these trials differed greatly in their intervention plan, study design and location (see table 1), and therefore, it was not appropriate to report an overall effect. Bobrow et al. and Owolabi et al. met all quality criteria, however Vedanthan et al.[48] failed to meet any, with authors describing difficulties in data collection and high levels of missing data. Some authors stated it had not been feasible to power studies to detect significant BP reduction, for example, the three month interim results of the PINGS trial did not find significant BP reduction due to the intervention until after nine months, when the proportion of participants with controlled BP became significantly higher in the intervention arm (46.7% vs 40%).[39 42] In some cases, authors noted that effects varied between subjects based on initial hypertension severity. An instance of this is Kingue et al., where greater overall improvements (BP improved or BP at target) in participants with stage 3 hypertension was observed.[28] Owolabi et al. also observed a significant reduction in blood pressure for a subset of subjects with baseline BP>140.90mmHg, but not an overall significant reduction for all participants.[41]

Figure 3. Change in mean systolic blood pressure (mmHg) between control and intervention groups.

In terms of health knowledge improvement, in one study CVD nursers reported that their own hypertension awareness and knowledge increased, as well as that of the community, due to the ComHIP project.[47] Hacking et al. found no statistical change in overall health knowledge, however medication adherence was significantly higher due to intervention and self-reported behaviour change improvements.[34] Haricharan et al. reported significant improvements in overall knowledge of healthy living and hypertension following exposure to informative SMS messaging.[35] PINGS resulted in significant medication adherence improvements,[39] and TELEMED-CAM saw significantly higher medical visit adherence in their intervention group.[28] Ola-Olorun et al. reported positive perceptions towards a SMS based medicine information exchange between patients and pharmacists, patients requested information on adverse medication effects.[29] Clinical documentation improved for all non-communicable diseases investigated by Kleczka et al., with a 21% improvement in hypertension documentation scoring.[36]

When perceptions towards the proposed technology were gathered, interventions were well received by patients[29 31 34 47] and health professionals.[29 37 44 47] Reported concerns included access to/stability of internet connection,[47] power availability,[37 44 47] cost,[44 47] increased workload,[38] understanding of SMS wording,[32] unfamiliarity with mobile technology or technology not being 'user-friendly',[34 44] and duplication in digital patient records.[49] Focus groups and interviews conducted by Adler et al. indicated that health providers and policy makers identified major challenges in the use of a cloud-based health records system, which would require heavy reliance on outside resources.[47]

Discussion

Our systematic review of the literature found broad and diverse applications of KETs for tackling hypertension in SSA. The findings indicated there is still relatively limited published research, particularly of controlled trials. All studies leveraged mobile phones for purposes of screening for hypertension, improving patient knowledge/treatment adherence or aiding non physician healthcare workers in providing hypertension care. Other reviews targeted to SSA have focused on assessing either specific technological applications or different NCDs and all noted a lack of published research.[18 20 50] Muiruri et al.'s 2019 narrative literature review of telehealth interventions for hypertension in SSA[50] identified just eight studies and in 2021 Osei et al.[18] identified only 12 studies in a scoping review of mobile health (mHealth) for diagnosis or treatment of any disease in SSA. These authors also commented as we do on the paucity of studies of robust design, particularly those including control groups.[18 50]

For peer review only - http://bmjopen.bmj.com/site/about/guidelines.xhtml

Overall, our identified studies reported success in their outcomes, with overwhelmingly positive responses from participants towards the use of KETs. Consistent with our findings, other reviews comment on overall good acceptance of technologies by health workers[18 20] and SMS for health knowledge improvement and behaviour changes were identified as providing particularly promising positive results.[50] However, we found very few studies were able to demonstrate statistically significant improvements over standard care, when evaluating objective measures such as BP reduction. This may indicate persistent difficulties in designing and implementing technology-based healthcare solutions in low-resource settings. Such difficulties were also evident from the quality analysis, for example a frequent issue identified in the quality analysis for both randomised and nonrandomised trials was a lack of complete outcome data, with authors describing difficulties with missing information and loss to follow-up as high as 50% in one study[35] and 58% in another,[48] an important consideration for future studies.

No publications using AI were identified in this study. Owoyemi et al.[51] suggested that reasons for this may include: limited available data, a lack of policy and legal framework, associated cost of uptake and inadequate infrastructure. Future research may explore predictive AI modelling either for screening and diagnostic tools, and to identify and target the most promising areas for addressing patient lifestyle changes in SSA.

Many studies utilised KETs to facilitate task redistribution, which is a well evidenced strategy to improving healthcare provision in areas with low numbers of qualified doctors/specialists, for which the body of evidence relating to hypertension is growing.[52-57] Mobile technology provided decision support and record keeping tools aiming to empower non physician workers in providing primary hypertension care. While perceptions and feedback from clinical staff and patients was overall positive, several key areas were consistently reported as major challenges for uptake of KETs. Fundamental issues in infrastructure are still a barrier to mobile technology for healthcare, evidenced by reported issues with internet, network and power coverage. In addition, several articles reported concerns around the ability of patients to use the technology and understand the information which was relayed, with calls for future research to investigate the feasibility and efficacy of audio visual rather than text communication.[34 42] Such findings have informed further development of the PINGS intervention, with upcoming phase III trials using a reductionist approach, removing a smartphone component and replacing with phone calls and audio and text messages.[58]

A notable finding was that few studies reported statistically significant benefits of KET based interventions. Authors speculated that small sample sizes,[40] subject selection,[34 42] failed SMS delivery,[34] study design such that all patients received reminders,[33 45] free medication[33] or financial incentives[41] could have contributed to this. Likely reflecting difficulty in retaining patients in care in SSA. It was also observed that interventions proved most effective among the highest risk groups, where it may be easiest to detect positive changes. Although not always found to be significant, reductions in BP were observed which, although modest, would, from a clinical perspective be anticipated to impact CVD development on a population level.[59] Our findings also indicated strategies using SMS to promote positive patient behaviour changes were highly successful.[34 39 47] It remains to be seen however, whether self-reported behaviour changes translate into objective improvements in blood pressure reduction.

Strengths and limitations of this study

A major limitation of this systematic review was the heterogeneity of the included studies, which did not allow for quantitative synthesis of outcomes/results. Since this study also failed to identify any reports of use of AI, it is possible that extending the search beyond the scientific literature may have found cases where AI was intrinsic as part of manufactured technology already being used for healthcare in SSA. A strength of this study is that it is the first systematic review concerning use of KETs for hypertension healthcare in SSA, and in this way provides a comprehensive overview of the current state of the art and indicates gaps to be addressed in future research.

Conclusion

Our study indicates there is limited research on use of KETs for hypertension in SSA, particularly we did not identify any studies using AI. The study demonstrates that mobile health strategies provided positive impact on BP control, health knowledge and treatment adherence. Further, stakeholder perceptions towards technology for hypertension prevention and management were positive. Therefore, further primary studies should be conducted, with an emphasis on objective measures such as BP reduction or BP control. It remains to be seen whether AI may also prove beneficial, such as through development of further diagnostic aids, or boosting signals from cheap easily manufactured sensors.

Contributors:
KS, LP and FC designed the study. LP and FC coordinated and supervised the study. KS, BO, LP and FC designed the data collection and methodology. KS and BO Screened records, extracted data, and assessed quality. KS wrote the original draft. All authors critically revised and edited the manuscript.

For peer review only - http://bmjopen.bmj.com/site/about/guidelines.xhtml

Data sharing statement: All data relevant to the study are included in the article or uploaded as supplementary information. No additional data available.
Declaration of interests: We declare no competing interests.

I, the Submitting Author has the right to grant and does grant on behalf of all authors of the Work (as defined in the below author licence), an exclusive licence and/or a non-exclusive licence for contributions from authors who are: i) UK Crown employees; ii) where BMJ has agreed a CC-BY licence shall apply, and/or iii) in accordance with the terms applicable for US Federal Government officers or employees acting as part of their official duties; on a worldwide, perpetual, irrevocable, royalty-free basis to BMJ Publishing Group Ltd ("BMJ") its licensees and where the relevant Journal is co-owned by BMJ to the co-owners of the Journal, to publish the Work in BMJ Open and any other BMJ products and to exploit all rights, as set out in our licence.

Licence statement
The Submitting Author accepts and understands that any supply made under these terms is made by BMJ to the Submitting Author unless you are acting as an employee on behalf of your employer or a postgraduate student of an affiliated institution which is paying any applicable article publishing charge ("APC") for Open Access articles. Where the Submitting Author wishes to make the Work available on an Open Access basis (and intends to pay the relevant APC), the terms of reuse of such Open Access shall be governed by a Creative Commons licence – details of these licences and which Creative Commons licence will apply to this Work are set out in our licence referred to above.

For peer review only

References

1. (WHO) WHO. Cardiovascular diseases (CVDs) [Fact sheet]. 2017 [Available from: https://www.who.int/en/news-room/fact-sheets/detail/cardiovascular-diseases-(cvds).
2. WHO. The top 10 causes of death [cited 2021. Available from: https://www.who.int/news-room/fact-sheets/detail/the-top-10-causes-of-death.
3. Dzudie A, Rayner B, Ojji D, et al. Roadmap to achieve 25% hypertension control in Africa by 2025. *Cardiovascular journal of Africa* 2017;28(4):262-72. doi: 10.5830/CVJA-2017-040
4. Nyirenda MJ. Non-communicable diseases in sub-Saharan Africa: understanding the drivers of the epidemic to inform intervention strategies. *Int Health* 2016;8(3):157-8. doi: 10.1093/inthealth/ihw021 [published Online First: 2016/05/15]
5. Ataklte F, Erqou S, Kaptoge S, et al. Burden of Undiagnosed Hypertension in Sub-Saharan Africa. *Hypertension* 2015;65(2):291-98. doi: doi:10.1161/HYPERTENSIONAHA.114.04394
6. Piette JD, List J, Rana GK, et al. Mobile Health Devices as Tools for Worldwide Cardiovascular Risk Reduction and Disease Management. *Circulation* 2015;132(21):2012-27. doi: 10.1161/CIRCULATIONAHA.114.008723
7. Chang LW, Njie-Carr V, Kalenge S, et al. Perceptions and acceptability of mHealth interventions for improving patient care at a community-based HIV/AIDS clinic in Uganda: a mixed methods study. *AIDS Care* 2013;25(7):874-80. doi: 10.1080/09540121.2013.774315 [published Online First: 2013/03/05]
8. Vélez O, Okyere PB, Kanter AS, et al. A usability study of a mobile health application for rural Ghanaian midwives. *J Midwifery Womens Health* 2014;59(2):184-91. doi: 10.1111/jmwh.12071 [published Online First: 2014/01/10]
9. Bruxvoort K, Festo C, Kalolella A, et al. Cluster randomized trial of text message reminders to retail staff in tanzanian drug shops dispensing artemether-lumefantrine: effect on dispenser knowledge and patient adherence. *Am J Trop Med Hyg* 2014;91(4):844-53. doi: 10.4269/ajtmh.14-0126 [published Online First: 2014/07/07]
10. Mahmud N, Rodriguez J, Nesbit J. A text message-based intervention to bridge the healthcare communication gap in the rural developing world. *Technology and Health Care* 2010;18:137-44. doi: 10.3233/THC-2010-0576
11. Kamanga A, Moono P, Stresman G, et al. Rural health centres, communities and malaria case detection in Zambia using mobile telephones: a means to detect potential reservoirs of infection in unstable transmission conditions. *Malar J* 2010;9:96. doi: 10.1186/1475-2875-9-96 [published Online First: 2010/04/20]
12. Rajput ZA, Mbugua S, Amadi D, et al. Evaluation of an Android-based mHealth system for population surveillance in developing countries. *J Am Med Inform Assoc* 2012;19(4):655-9. doi: 10.1136/amiajnl-2011-000476 [published Online First: 2012/03/01]
13. World Health A. mHealth: use of appropriate digital technologies for public health: report by the Director-General. Geneva: World Health Organization, 2018.
14. WHO. A resolution on eHealth adopted at the 58th World Health Assembly, 2005.
15. GSMA. Mobile Economy Sub-Saharan Africa, 2019.
16. White A, Thomas DS, Ezeanochie N, et al. Health Worker mHealth Utilization: A Systematic Review. *Comput Inform Nurs* 2016;34(5):206-13. doi: 10.1097/cin.0000000000000231 [published Online First: 2016/03/10]
17. Braun R, Catalani C, Wimbush J, et al. Community health workers and mobile technology: a systematic review of the literature. *PLoS One* 2013;8(6):e65772. doi: 10.1371/journal.pone.0065772 [published Online First: 2013/06/19]
18. Osei E, Kuupiel D, Vezi PN, et al. Mapping evidence of mobile health technologies for disease diagnosis and treatment support by health workers in sub-Saharan Africa: a scoping review. *BMC Medical Informatics and Decision Making* 2021;21(1):11. doi: 10.1186/s12911-020-01381-x
19. Stephani V, Opoku D, Quentin W. A systematic review of randomized controlled trials of mHealth interventions against non-communicable diseases in developing countries. *BMC Public Health* 2016;16(1):572. doi: 10.1186/s12889-016-3226-3
20. Bloomfield GS, Vedanthan R, Vasudevan L, et al. Mobile health for non-communicable diseases in Sub-Saharan Africa: a systematic review of the literature and strategic framework for research. *Global Health* 2014;10:49. doi: 10.1186/1744-8603-10-49 [published Online First: 2014/06/15]
21. Jiang F, Jiang Y, Zhi H, et al. Artificial intelligence in healthcare: past, present and future. *Stroke and Vascular Neurology* 2017;2(4):230. doi: 10.1136/svn-2017-000101
22. Wahl B, Cossy-Gantner A, Germann S, et al. Artificial intelligence (AI) and global health: how can AI contribute to health in resource-poor settings? *BMJ Global Health* 2018;3(4):e000798. doi: 10.1136/bmjgh-2018-000798

For peer review only

23. Alami H, Rivard L, Lehoux P, et al. Artificial intelligence in health care: laying the Foundation for Responsible, sustainable, and inclusive innovation in low- and middle-income countries. *Globalization and Health* 2020;16(1):52. doi: 10.1186/s12992-020-00584-1
24. Moher D, Liberati A, Tetzlaff J, et al. Preferred Reporting Items for Systematic Reviews and Meta-Analyses: The PRISMA Statement. *PLOS Medicine* 2009;6(7):e1000097. doi: 10.1371/journal.pmed.1000097
25. Akl E, Altman D, Aluko P, et al. Cochrane Handbook for Systematic Reviews of Interventions2019.
26. Wallace BC, Dahabreh IJ, Trikalinos TA, et al. Closing the Gap between Methodologists and End-Users: R as a Computational Back-End. *Journal of Statistical Software; Vol 1, Issue 5 (2012)* 2012
27. Hong QN, Gonzalez-Reyes A, Pluye P. Improving the usefulness of a tool for appraising the quality of qualitative, quantitative and mixed methods studies, the Mixed Methods Appraisal Tool (MMAT). *J Eval Clin Pract* 2018;24(3):459-67. doi: 10.1111/jep.12884 [published Online First: 2018/02/22]
28. Kingue S, Angandji P, Menanga AP, et al. Efficiency of an intervention package for arterial hypertension comprising telemanagement in a Cameroonian rural setting: The TELEMED-CAM study. *The Pan African medical journal* 2013;15:153-53. doi: 10.11604/pamj.2013.15.153.2655
29. Ola-Olorun N, Afolabi MO, Oyebisi T, et al. Exploring Medicine Information Needs of Hypertensive Patients Using Short Message Service. *British Journal of Medicine and Medical Research* 2014;4:5368-80. doi: 10.9734/BJMMR/2014/8858
30. Joubert J, Nkomazana O, Mompati K, et al. A community survey of cardiovascular risk factors in an urban population in Botswana exploring potential for telemedicine. *European Research in Telemedicine / La Recherche Européenne en Télémédecine* 2014;3(3):95-103. doi: https://doi.org/10.1016/j.eurtel.2014.07.001
31. Leon N, Surender R, Bobrow K, et al. Improving treatment adherence for blood pressure lowering via mobile phone SMS-messages in South Africa: a qualitative evaluation of the SMS-text Adherence SuppoRt (StAR) trial. *BMC Fam Pract* 2015;16:80. doi: 10.1186/s12875-015-0289-7 [published Online First: 2015/07/04]
32. Vedanthan R, Blank E, Tuikong N, et al. Usability and feasibility of a tablet-based Decision-Support and Integrated Record-keeping (DESIRE) tool in the nurse management of hypertension in rural western Kenya. *Int J Med Inform* 2015;84(3):207-19. doi: 10.1016/j.ijmedinf.2014.12.005 [published Online First: 2015/01/24]
33. Bobrow K, Farmer AJ, Springer D, et al. Mobile Phone Text Messages to Support Treatment Adherence in Adults With High Blood Pressure (SMS-Text Adherence Support [StAR]): A Single-Blind, Randomized Trial. *Circulation* 2016;133(6):592-600. doi: 10.1161/circulationaha.115.017530 [published Online First: 2016/01/16]
34. Hacking D, Haricharan HJ, Brittain K, et al. Hypertension Health Promotion via Text Messaging at a Community Health Center in South Africa: A Mixed Methods Study. *JMIR Mhealth Uhealth* 2016;4(1):e22. doi: 10.2196/mhealth.4569 [published Online First: 2016/03/12]
35. Haricharan HJ, Heap M, Hacking D, et al. Health promotion via SMS improves hypertension knowledge for deaf South Africans. *BMC Public Health* 2017;17(1):663. doi: 10.1186/s12889-017-4619-7
36. Kleczka B, Musiega A, Rabut G, et al. Rubber stamp templates for improving clinical documentation: A paper-based, m-Health approach for quality improvement in low-resource settings. *Int J Med Inform* 2018;114:121-29. doi: 10.1016/j.ijmedinf.2017.10.014 [published Online First: 2017/11/07]
37. Mannik JR, Figol A, Churchill V, et al. Community-based screening for cardiovascular risk using a novel mHealth tool in rural Kenya. *BMJ Health & Care Informatics* 2018;25(3):176. doi: 10.14236/jhi.v25i3.1012
38. Nelissen HE, Cremers AL, Okwor TJ, et al. Pharmacy-based hypertension care employing mHealth in Lagos, Nigeria - a mixed methods feasibility study. *BMC Health Serv Res* 2018;18(1):934. doi: 10.1186/s12913-018-3740-3 [published Online First: 2018/12/06]
39. Sarfo F, Treiber F, Gebregziabher M, et al. PINGS (Phone-Based Intervention Under Nurse Guidance After Stroke): Interim Results of a Pilot Randomized Controlled Trial. *Stroke* 2018;49(1):236-39. doi: 10.1161/strokeaha.117.019591 [published Online First: 2017/12/10]
40. Vedanthan R, Kamano JH, DeLong AK, et al. Community Health Workers Improve Linkage to Hypertension Care in Western Kenya. *J Am Coll Cardiol* 2019;74(15):1897-906. doi: 10.1016/j.jacc.2019.08.003 [published Online First: 2019/09/06]
41. Owolabi MO, Gebregziabher M, Akinyemi RO, et al. Randomized Trial of an Intervention to Improve Blood Pressure Control in Stroke Survivors. *Circ Cardiovasc Qual Outcomes* 2019;12(12):e005904. doi: 10.1161/circoutcomes.119.005904 [published Online First: 2019/12/07]
42. Sarfo FS, Treiber F, Gebregziabher M, et al. Phone-based intervention for blood pressure control among Ghanaian stroke survivors: A pilot randomized controlled trial. *Int J Stroke* 2019;14(6):630-38. doi: 10.1177/1747493018816423 [published Online First: 2018/11/23]

For peer review only

43. Nichols M, Singh A, Sarfo FS, et al. Post-intervention qualitative assessment of mobile health technology to manage hypertension among Ghanaian stroke survivors. *Journal of the Neurological Sciences* 2019;406:116462. doi: https://doi.org/10.1016/j.jns.2019.116462
44. Cremers AL, Alege A, Nelissen HE, et al. Patients' and healthcare providers' perceptions and practices regarding hypertension, pharmacy-based care, and mHealth in Lagos, Nigeria: a mixed methods study. *Journal of hypertension* 2019;37(2):389-97. doi: 10.1097/HJH.0000000000001877
45. Barsky J, Hunter R, McAllister C, et al. Analysis of the Implementation, User Perspectives, and Feedback From a Mobile Health Intervention for Individuals Living With Hypertension (DREAM-GLOBAL): Mixed Methods Study. *JMIR Mhealth Uhealth* 2019;7(12):e12639. doi: 10.2196/12639 [published Online First: 2019/12/10]
46. Oduor E, Pang C, Wachira C, et al. Exploring Rural Community Practices in HIV Management for the Design of Technology for Hypertensive Patients Living with HIV. Proceedings of the 2019 on Designing Interactive Systems Conference. San Diego, CA, USA: Association for Computing Machinery, 2019:1595–606.
47. Adler AJ, Laar AK, Kotoh AM, et al. Barriers and facilitators to the implementation of a community-based hypertension improvement project in Ghana: a qualitative study of ComHIP. *BMC Health Services Research* 2020;20(1):67. doi: 10.1186/s12913-019-4774-x
48. Vedanthan R, Kumar A, Kamano JH, et al. Effect of Nurse-Based Management of Hypertension in Rural Western Kenya. *Glob Heart* 2020;15(1):77. doi: 10.5334/gh.856 [published Online First: 2020/12/11]
49. Aw M, Ochieng BO, Attambo D, et al. Critical appraisal of a mHealth-assisted community-based cardiovascular disease risk screening program in rural Kenya: an operational research study. *Pathog Glob Health* 2020;114(7):379-87. doi: 10.1080/20477724.2020.1816286 [published Online First: 2020/09/09]
50. Muiruri C, Manavalan P, Jazowski SA, et al. Opportunities to Leverage Telehealth Approaches Along the Hypertension Control Cascade in Sub-Saharan Africa. *Curr Hypertens Rep* 2019;21(10):75. doi: 10.1007/s11906-019-0983-2 [published Online First: 2019/08/28]
51. Owoyemi A, Owoyemi J, Osiyemi A, et al. Artificial Intelligence for Healthcare in Africa. *Frontiers in Digital Health* 2020;2(6) doi: 10.3389/fdgth.2020.00006
52. Anand TN, Joseph LM, Geetha AV, et al. Task sharing with non-physician health-care workers for management of blood pressure in low-income and middle-income countries: a systematic review and meta-analysis. *The Lancet Global Health* 2019;7(6):e761-e71. doi: 10.1016/S2214-109X(19)30077-4
53. Labhardt ND, Balo JR, Ndam M, et al. Task shifting to non-physician clinicians for integrated management of hypertension and diabetes in rural Cameroon: a programme assessment at two years. *BMC Health Serv Res* 2010;10:339. doi: 10.1186/1472-6963-10-339 [published Online First: 2010/12/15]
54. Iwelunmor J, Gyamfi J, Plange-Rhule J, et al. Exploring stakeholders' perceptions of a task-shifting strategy for hypertension control in Ghana: a qualitative study. *BMC Public Health* 2017;17(1):216. doi: 10.1186/s12889-017-4127-9
55. Ogedegbe G, Plange-Rhule J, Gyamfi J, et al. Health insurance coverage with or without a nurse-led task shifting strategy for hypertension control: A pragmatic cluster randomized trial in Ghana. *PLoS Med* 2018;15(5):e1002561. doi: 10.1371/journal.pmed.1002561 [published Online First: 2018/05/02]
56. Kengne AP, Awah PK, Fezeu LL, et al. Primary health care for hypertension by nurses in rural and urban sub-Saharan Africa. *J Clin Hypertens (Greenwich)* 2009;11(10):564-72. doi: 10.1111/j.1751-7176.2009.00165.x [published Online First: 2009/10/13]
57. Some D, Edwards JK, Reid T, et al. Task Shifting the Management of Non-Communicable Diseases to Nurses in Kibera, Kenya: Does It Work? *PLOS ONE* 2016;11(1):e0145634. doi: 10.1371/journal.pone.0145634
58. Sarfo FSA, A.; Bockarie, A.; Appiah, L.; Nguah, S. B.; Ayisi-Boateng, N. K.; Adamu, S.; Neizer, C.; Arthur, A.; Nyamekye, R.; Agyenim-Boateng, K.; Tagge, R.; Adusei-Mensah, N.; Ampofo, M.; Laryea, R.; Singh, A.; Amuasi, J. H.; Ovbiagele, B. Phone-Based Intervention under Nurse Guidance after Stroke (PINGS II) Study: Protocol for a Phase III Randomized Clinical Trial. *Journal of Stroke & Cerebrovascular Diseases* 2021;30(8):105888.
59. Ettehad D, Emdin CA, Kiran A, et al. Blood pressure lowering for prevention of cardiovascular disease and death: a systematic review and meta-analysis. *The Lancet* 2016;387(10022):957-67. doi: 10.1016/S0140-6736(15)01225-8

For peer review only - http://bmjopen.bmj.com/site/about/guidelines.xhtml

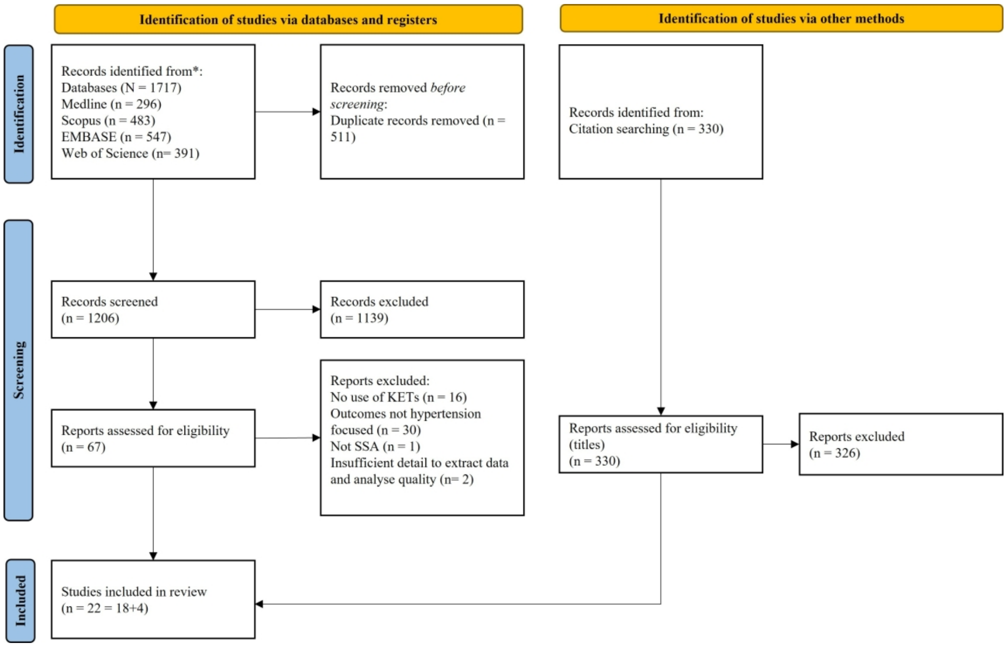

Figure 1. Study selection

258x164mm (330 x 330 DPI)

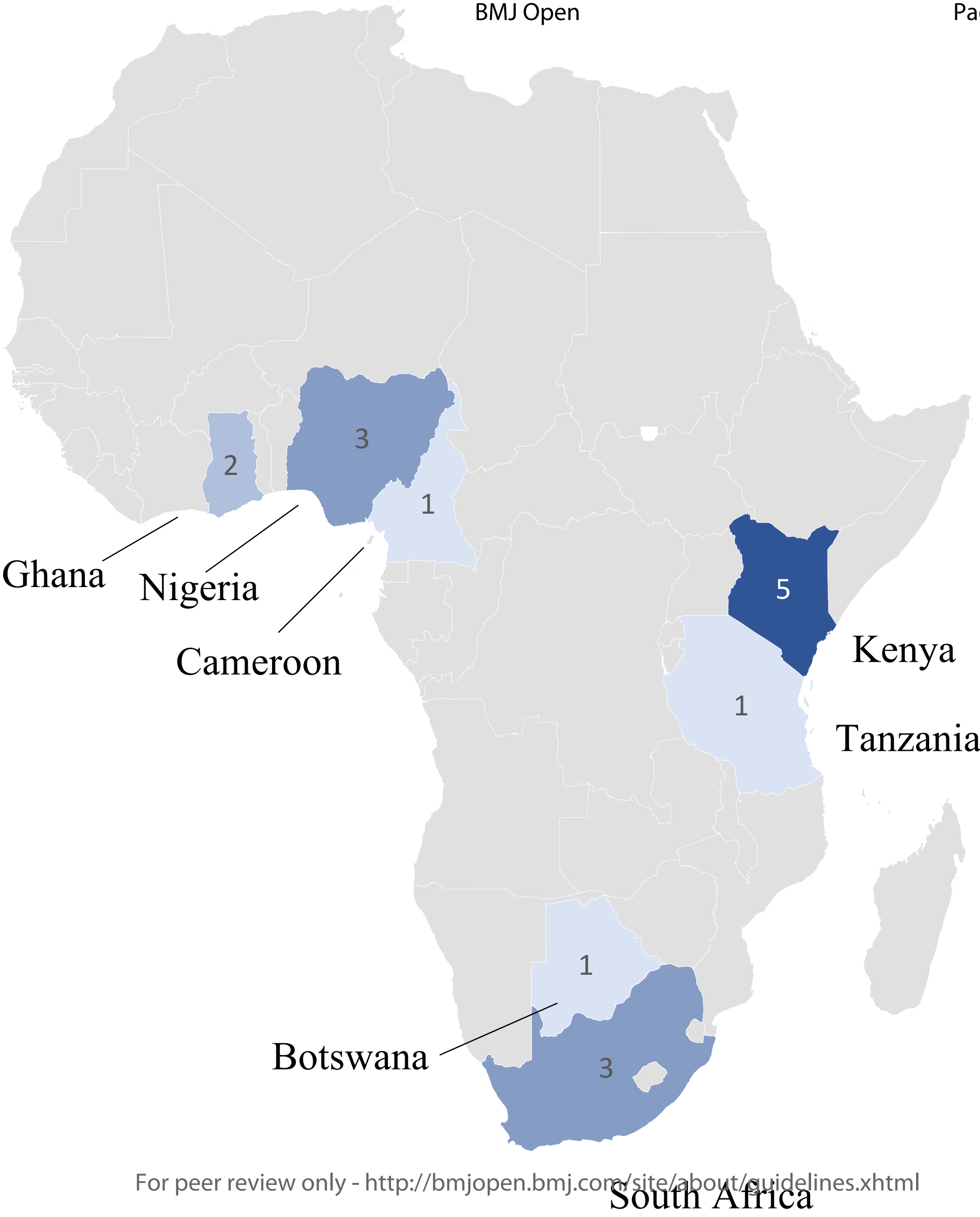
3
2
1
5
1
1
3
Ghana
Nigeria
Cameroon
Kenya
Tanzania
Botswana
South Africa

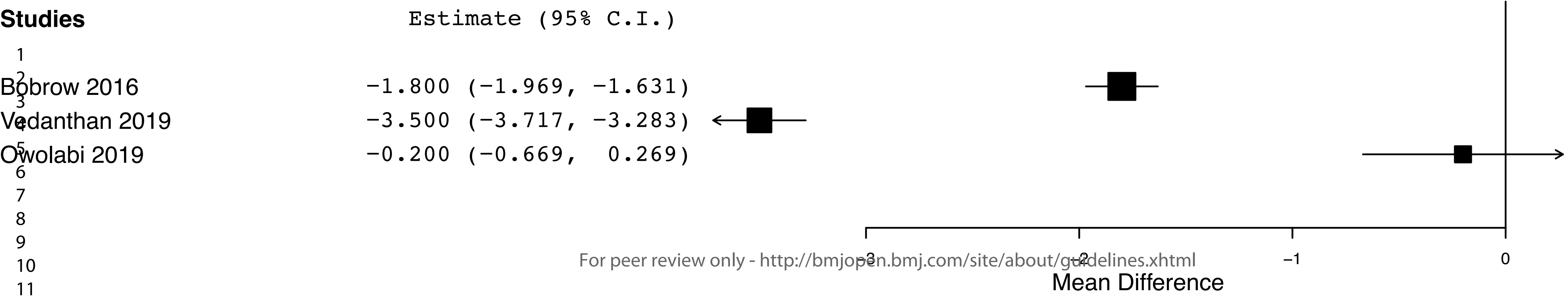

For peer review only - http://bmjopen.bmj.com/site/about/guidelines.xhtml

Test S1. Search terms

MEDLINE/Embase:
exp Cardiovascular Diseases/ or exp Hypertension/ or ("cardiovascular disease*" or CVD* or "hypertensi*" or "high blood pressure" or "blood pressure" or stroke or "heart disease").tw. and exp "Africa South of the Sahara"/ or (Sahara* or sub-Sahara* or "sub-Sahara* Africa" or SSA or Cameroon or "Central African Republic" or Chad or Congo or "democratic republic of the Congo" or "equatorial Guinea" or Gabon or "Sao Tome and Principe" or Burundi or Djibouti or Eritrea or Ethiopia or Kenya or Rwanda or Somalia or "south Sudan" or Sudan or Tanzania or Uganda or Angola or Botswana or Eswatini or Lesotho or Malawi or Mozambique or Namibia or Zambia or Zimbabwe or Benin or "Burkina Faso" or "Cabo Verde" or "Cote d'Ivoire" or Gambia or Ghana or Guinea or "Guinea-Bissau" or Liberia or Mali or Mauritania or Niger or Nigeria or Senegal or "Sierra Leone" or Togo).tw. and exp Artificial Intelligence/ or exp Artificial Intelligence/ or exp Cell Phone/ or exp "Internet of Things"/ or exp Telemedicine/ or ("artificial intelligence" or ai or "machine learning" or "deep learning" or "neural network*" or "reinforcement learning" or "naive bayes" or "decision tree" or "random forest" or "support vector machine" or "k-nearest neighbour" or "linear discriminant analysis" or classification or clustering or "supervised learning" or telehealth or telemedicine or mhealth or "mobile phone" or smartphone or "internet of things" or "cell phone").tw. OR (exp Artificial Intelligence/ or ("artificial intelligence" or ai or "machine learning").tw.) and ("logistic regression" or "logit regression" or "multiple regression").tw. and exp diagnosis/ or exp therapeutics exp treatment outcome/ or (prevent* or screen* or detection or diagnos* or management or treatment or control).tw.

Web of Science:
TS = (prevent* or screen* or diagnos* or treatment or therapeutics or management or control) and S =("artificial intelligence" or ai or "machine learning" or "deep learning" or "neural network*" or "reinforcement learning" or "naive bayes" or "decision tree" or "random forest" or "support vector machine" or "k-nearest neighbour" or "linear discriminant analysis" or classification or clustering or "supervised learning" or telehealth or telemedicine or mhealth or "mobile phone" or smartphone or "internet of things" or "cell phone") OR (TS=("artificial intelligence" or ai or "machine learning") AND TS=("logistic regression" or "logit regression" or "multiple regression")) and TS = ("Africa south of Sahara" or "Sahara" or "sub-Sahara*" or "sub-Sahara* Africa" or SSA or Cameroon or “Central African Republic” or Chad or Congo or “democratic republic of the Congo” or “equatorial guinea” or Gabon or “Sao Tome and Principe” or Burundi or Djibouti or Eritrea or Ethiopia or Kenya or Rwanda or Somalia or “South Sudan” or Sudan or Tanzania or Uganda or Angola or Botswana or Eswatini or Lesotho or Malawi or Mozambique or Namibia or Zambia or Zimbabwe or Benin or “Burkina Faso” or “Cabo Verde” or “Cote d’Ivoire” or Gambia or Ghana or Guinea or “Guinea-Bissau” or Liberia or Mali or Mauritania or Niger or Nigeria or Senegal or “Sierra Leone” or Togo) and TS=(cardiovascular disease CVD* or hypertensi* or high blood pressure or blood pressure or stroke or heart disease)

Scopus:
((TITLE-ABS-KEY ("cardiovascular disease*" OR cvd* OR "hypertensi*" OR "high blood pressure" OR "blood pressure" OR stroke OR "heart disease"))) AND (TITLE-ABS-KEY ("artificial intelligence" OR ai OR "machine learning" OR "deep learning" OR "neural network*" OR "reinforcement learning" OR "naive bayes" OR "decision tree" OR "random forest" OR "support vector machine" OR "k-nearest neighbour" OR "linear discriminant analysis" OR classification OR clustering OR "supervised learning" OR telehealth OR telemedicine OR mhealth OR "mobile phone" OR smartphone OR "internet of things" OR "cell phone") OR (TITLE-ABS-KEY ("artificial intelligence" OR ai OR "machine learning") AND TITLE-ABS-KEY ("logistic regression" OR "logit regression" OR "multiple regression"))) AND (TITLE-ABS-KEY ("Africa south of sahara" OR "sahara" OR "sub-sahara*" OR "sub-sahara* africa" OR ssa OR cameroon OR "Central African Republic" OR chad OR congo OR "democratic republic of the Congo" OR "equatorial guinea" OR gabon OR "Sao Tome and Principe" OR burundi OR djibouti OR eritrea OR ethiopia OR kenya OR rwanda OR somalia OR "South Sudan" OR sudan OR tanzania OR uganda OR angola OR botswana OR eswatini OR lesotho OR malawi OR mozambique OR namibia OR zambia OR zimbabwe OR benin OR "Burkina Faso" OR "Cabo Verde" OR "Cote d'Ivoire" OR gambia OR ghana OR guinea OR "Guinea-Bissau" OR liberia OR mali OR mauritania OR niger OR nigeria OR senegal OR "Sierra Leone" OR togo)) AND (TITLE-ABS-KEY (prevent* OR screen* OR diagnos* OR treatment OR therapeutics OR management OR control))

For peer review only

Table S1. Articles excluded on reading full text.

Authors	Source	Year	Title	Reason:
T.; Njuguna Mercer, B.; Bloomfield, G. S.; Dick, J.; Finkelstein, E.; Kamano, J.; Mwangi, A.; Naanyu, V.; Pastakia, S. D.; Valente, T. W.; Vedanthan, R.; Akwanalo, C.	Trials	2019	Strengthening Referral Networks for Management of Hypertension Across the Health System (STRENGTHS) in western Kenya: a study protocol of a cluster randomized trial	No KETs
I.; Joloba Ddumba, S.; Kakande, B.	Transactions of the Royal Society of Tropical Medicine and Hygiene	2019	Motivations for participating in the use of mobile smart phone app focusing on monitoring of hypertension among older persons in Uganda	Insufficient detail
F. S.; Adusei Sarfo, N.; Ampofo, M.; Kpeme, F. K.; Ovbiagele, B.	Journal of the Neurological Sciences	2018	Pilot trial of a tele-rehab intervention to improve outcomes after stroke in Ghana: A feasibility and user satisfaction study	Not hypertension
F. S.; Adamu Sarfo, S.; Awuah, D.; Sarfo-Kantanka, O.; Ovbiagele, B.	Journal of the Neurological Sciences	2017	Potential role of tele-rehabilitation to address barriers to implementation of physical therapy among West African stroke survivors: A cross-sectional survey	Not hypertension
F.; Pudney Lorgat, E.; van Deventer, H.; Chitsaz, S.	Cardiovascular Journal of Africa	2012	Robotically controlled ablation for atrial fibrillation: the first real-world experience in Africa with the Hansen robotic system	Not hypertension
R.; Kamano Vedanthan, J. H.; Naanyu, V.; Delong, A. K.; Were, M. C.; Finkelstein, E. A.; Menya, D.; Akwanalo, C. O.; Bloomfield, G. S.; Binanay, C. A.; Velazquez, E. J.; Hogan, J. W.; Horowitz, C. R.; Inui, T. S.; Kimaiyo, S.; Fuster, V.	Trials [Electronic Resource]	2014	Optimizing linkage and retention to hypertension care in rural Kenya (LARK hypertension study): study protocol for a randomized controlled trial	No KETs
E.; Nyota Oduor, T.; Wachira, C.; Osebe, S.; Remy, S. L.; Walcott, A.	Journal	Year	Medication Management Companion (MMC) for a rural kenyan community	No KETs
J. T.; Tham Kamwesiga, K.; Guidetti, S.	Disability and Rehabilitation	2017	Experiences of using mobile phones in everyday life among persons with stroke and their families in Uganda - a qualitative study	Not hypertension
J. T.; Eriksson Kamwesiga, G. M.; Tham, K.; Fors, U.; Ndiwalana, A.; von Koch, L.; Guidetti, S.	Global Health	2018	A feasibility study of a mobile phone supported family-centred ADL intervention, F@ce TM, after stroke in Uganda	Not hypertension
F. K.; Adoubi Diby, A.; Gnaba, A.; Ouattara, P.; Ayegnon, K. G.; Boidy, K.; Azagoh-Kouadio, R.; Meneas, G.; Manga, D.; Coulibaly, A.; Sall, F.; Nguessan, E.; Ehui, E.; Yangni-Angate, K. H.	European Research in Telemedicine	2015	Tele-expertise in the interpretation of the electrocardiogram of a black African population in the Ivory Coast (sub-Saharan Africa)	Not hypertension
G. S.; Wang Bloomfield, T. Y.; Boulware, L. E.; Califf, R. M.; Hernandez, A. F.; Velazquez, E. J.; Peterson, E. D.; Li, J. S.	Global heart	2015	Implementation of management strategies for diabetes and hypertension: from local to global health in cardiovascular diseases	No KETs
T.; Dewyer Aliku, A.; Namuyonga, J.; Ssinabulya, I.; Kamarembo, J.; Okello, E.; Bua, B.; Asiimwe, A.; Odong, F.; Akech, R.; Beaton, A.; DeStigter, K.; Lwabi, P.; Sable, C.	Global Heart	2018	Telemedicine Support of Cardiac Care In Northern Uganda: Leveraging Hand-held Echocardiography and Task Shifting	Not hypertension
M.; Sarfo Nichols, F. S.; Singh, A.; Qanungo, S.; Treiber, F.; Ovbiagele, B.; Saulson, R.; Patel, S.; Jenkins, C.	American Journal of the Medical Sciences	2017	Assessing Mobile Health Capacity and Task Shifting Strategies to Improve Hypertension Among Ghanaian Stroke Survivors	Not hypertension
H. L.; Duhig Nathan, K.; Vousden, N.; Lawley, E.; Seed, P. T.; Sandall, J.; Bellad, M. B.; Brown, A. C.; Chappell, L. C.; Goudar, S. S.; Gidiri, M. F.; Shennan, A. H.; Cradle- Trial Collaboration Grp	Trials	2018	Evaluation of a novel device for the management of high blood pressure and shock in pregnancy in low-resource settings: study protocol for a stepped-wedge cluster-randomised controlled trial (CRADLE-3 trial)	No KETs
J. S.; Inyiama Igwe, H. C.; Alo, U. R.; Ajah, I. A.	International Journal of Scientific and Technology Research	2020	Interpretation of eeg recordings for the purpose of diagnosing stroke disease	Not hypertension
J. S.; Inyiama Igwe, H. C.; Alo, U. R.; Ajah, I. A.	Journal		Classification of human brain signal for diagnosis of stroke disease using artificial neural network	Not hypertension
O.; Olabode Olabode, B. T.	Journal of Computer Science	2012	Cerebrovascular accident attack classification using multilayer feed forward artificial neural network with back propagation error	Not hypertension
N.; Imberti Maurizi, J. F.; Faragli, A.; Targetti, M.; Baldini, K.; Sall, A.; Cisse, A.; Gigli Berzolari, F.; Borrelli, P.; Avvantaggiato, F.; Perlini, S.; Marchionni, N.; Cecchi, F.; Parigi, G. B.; Olivotto, I.	European Heart Journal	2016	Comparative analysis of a 4-lead portable smartphone-based versus standard 12-lead electrocardiograph for cardiovascular screening in low-income settings	Not hypertension
N.; Faragli Maurizi, A.; Imberti, J.; Briante, N.; Targetti, M.; Baldini, K.; Sall, A.; Cisse, A.; Berzolari, F. G.; Borrelli, P.; Avvantaggiato, F.; Perlini, S.; Marchionni, N.; Cecchi, F.; Parigi, G.; Olivotto, I.	International Journal of Cardiology	2017	Cardiovascular screening in low-income settings using a novel 4-lead smartphone-based electrocardiograph (D-Heart®)	Not hypertension
J. J.; Salehian Manolakos, O.; Kraeker, C.; Manolakos, L.; Hunter, C. J.	Canadian Journal of Cardiology	2015	Rheumatic heart disease screening in Windhoek Namibia using portable echocardiography: A pilot project	Not hypertension
G. F.; Shirk Evans, A.; Muturi, P.; Soliman, E. Z.	Global Heart	2017	Feasibility of Using Mobile ECG Recording Technology to Detect Atrial Fibrillation in Low-Resource Settings	Not hypertension

For peer review only

Table S2. Quality analysis

	RefID	31	32	40	28	41	34	39	42	43	47	33	48	38	44	37	49	29	36	35	46	30	45
	**First author**	**Leon**	**Bobrow**	**Vedanthan**	**Kingue**	**Owolabi**	**Hacking**	**Sarfo**	**Sarfo**	**Nichols**	**Adler**	**Vedanthan**	**Vedanthan**	**Nelissen**	**Cremers**	**Mannik**	**Aw**	**Ola-Olorun**	**Kleczka**	**Haricharan**	**Odour**	**Joubert**	**Barsky**
	**Year**	**2015**	**2016**	**2019**	**2013**	**2019**	**2016**	**2018**	**2019**	**2019**	**2020**	**2015**	**2020**	**2018**	**2019**	**2018**	**2020**	**2014**	**2018**	**2017**	**2019**	**2014**	**2019**
**SCREENING QUESTIONS**	S1. Are there clear research questions?	Yes	Yes	Yes	Yes	Yes	Yes	Yes	Yes	Yes	Yes	Yes	Yes	Yes	Yes	Yes	Yes	Yes	Yes	Yes	Yes	Yes	Yes
	S2. Do the collected data allow to address the research questions?	Yes	Yes	Yes	Yes	Yes	Yes	Yes	Yes	Yes	Yes	Yes	Yes	Yes	Yes	Yes	Yes	Yes	Yes	Yes	Yes	Yes	Yes
**1. QUALITATIVE STUDIES**	1.1. Is the qualitative approach appropriate to answer the research question?	Yes					Yes			Yes	Yes	Yes		Yes	Yes	Yes	Yes	Yes		Yes	Yes		Yes
	1.2. Are the qualitative data collection methods adequate to address the research question?	Yes					Yes			Yes	Yes	Yes		Yes	Yes	Yes	Yes	Yes		Yes	Yes		Yes
	1.3. Are the findings adequately derived from the data?	Yes					Yes			Yes	Yes	Yes		Yes	Yes	Can't tell	Yes	Yes		Yes	Yes		Can't tell
	1.4. Is the interpretation of results sufficiently substantiated by data?	Yes					Yes			Yes	Yes	Yes		Yes	Yes	No	Yes	Yes		Yes	Yes		Can't tell
	1.5. Is there coherence between qualitative data sources, collection, analysis and interpretation?	Yes					Yes			Yes	Yes	Yes		Yes	Yes	Yes	Yes	Yes		Yes	Yes		Can't tell
**2. RANDOMIZED CONTROLLED TRIALS**	2.1. Is randomization appropriately performed?		Yes	No	No	Yes	No	Yes	Yes														Yes
	2.2. Are the groups comparable at baseline?		Yes	No	No	Yes	Yes	Yes	Yes														Can't tell
	2.3. Are there complete outcome data?		Yes	No	Yes	Yes	No	Yes	Yes														Can't tell
	2.4. Are outcome assessors blinded to the intervention provided?		Yes	No	No	Yes	Can't tell	Yes	Yes														Can't tell
	2.5 Did the participants adhere to the assigned intervention?		Yes	Can't tell	Yes	Yes	Yes	Yes	Yes														Can't tell
**3. NON-RANDOMIZED STUDIES**	3.1. Are the participants representative of the target population?												Yes	Yes	Yes			Yes	Yes	No			
	3.2. Are measurements appropriate regarding both the outcome and intervention (or exposure)?												Yes	No	Yes			Yes	Yes	Yes			
	3.3. Are there complete outcome data?												No	No	No			Yes	Yes	No			
	3.4. Are the confounders accounted for in the design and analysis?												Yes	Yes	Can't tell			Can't tell	Yes	Yes			
	3.5. During the study period, is the intervention administered (or exposure occurred) as intended?												No	No	Yes			Yes	Yes	Yes			
**4. QUANTITATIVE DESCRIPTIVE STUDIES**	4.1. Is the sampling strategy relevant to address the research question?															Yes	Yes					Yes	
	4.2. Is the sample representative of the target population?															Yes	No					Yes	
	4.3. Are the measurements appropriate?															Yes	Yes					Yes	
	4.4. Is the risk of nonresponse bias low?														Yes	Yes					Yes		
	4.5. Is the statistical analysis appropriate to answer the research question?														Yes	Yes					Yes		
**5. MIXED METHODS STUDIES**	5.1. Is there an adequate rationale for using a mixed methods design to address the research question?					Yes							Yes	Yes	Yes	Yes	Yes		Yes			Yes	
	5.2. Are the different components of the study effectively integrated to answer the research question?					Yes							Yes	Yes	Yes	Yes	Yes		Yes			Yes	
	5.3. Are the outputs of the integration of qualitative and quantitative components adequately interpreted?					Yes							Yes	Yes	Can't tell	Yes	Yes		Yes			Can't tell	
	5.4. Are divergences and inconsistencies between quantitative and qualitative results adequately addressed?					Yes							Yes	Can't tell	Yes	Yes	Yes		Yes			Can't tell	
	5.5. Do the different components of the study adhere to the quality criteria of each tradition of the methods involved?					No							No	Yes	Yes	Yes	Yes		No			No	
	% criteria met	100	100	0	40	90	70	100	100	100	100	100	70	73.33333333	82.35294118	86.66666667	92.30769231	90	90	80	100	62.5	30

For peer review only

Table S3. Extracted baseline and endpoint systolic blood pressure readings for randomised controlled trials

Author	Title	Year	Duration	Intervention type	Primary outcome	Secondary outcome	Monitoring tools	Groups	Number of participants (baseline)	Number of participants (endpoint)	Groups
Bobrow et al.	Mobile Phone Text Messages to Support Treatment Adherence in Adults With High Blood Pressure (StAR): A Single-Blind, Randomized Trial	2016	12 months	SMS-Text Adherence suppoRt. Patients with high BP received SMS. Information only: Messages for motivation for medicine collection/taking or attending clinic, messages for education of hypertension. Interactive: Received same messages and were able to respond for changing an appointment, and changing the timing and language of the text-messages	Change in Systolic Blood Pressure at 12-months from baseline	Proportion of participants achieving mean SBP <140mmHg and mean DBP <90mmHg.<br>Health status (measured by self report questionnarire).<br>Proportion of scheduled clinic appointments attended.<br>Retention in clinical care. Satisfaction with clinic services and care.<br>Hospital admissions.<br>Self-reported adherence to medication.<br>Basic hypertension knowledge.<br>Number and type of medication changes made during trial.<br>Number of clinic visits.	validated oscillometric device. Recorded six sequential readings at three-minute intervals. The mean blood pressure was calculated by discarding the initial reading and calculating the mean from the five remaining readings.	Usual Care (UC)<br>Information Only (IO)<br>Interactive (I)			
								**UC**	457	396	**UC**
								**IO**	457	406	**IO**
								**I**	458	394	**I**
Vedanthan et al.	Community Health Workers Improve Linkage to Hypertension Care in Western Kenya	2019	15 months	Patients with high BP. Tailored behavioral communication + mHealth. Paper based: Community health worker ugave tailored behavioural and motivational engagement. Smartphone: CHW did same as PB, but had real-time decision support and data entry linked to electronic health record.<br>Cluster randomised	Co-primary outcomes:<br>Linkage to care<br>Change in SBP	N/A	automated Omron blood pressure machine, standard protocol (as described by World Health Organisation)	Usual Care (UC)<br>Paper Based (PB)<br>Smartphone (SP)			
								**UC**	422	355	**UC**
								**PB**	451	395	**PB**
								**SP**	465	356	**SP**
Owolabi et al.	Randomized Trial of an Intervention to Improve Blood Pressure Control in Stroke Survivors	2019	12 months	Patients with stroke-onset within one year. Intervention: chronic care model components of delivery system redesign (increased follow-up visits, pre-appointment phone texts), self-management support (patient report card, post-clinic follow-up phone texts, waiting room educational video), and clinical information systems (patient report card as part of medical chart, hospital registry).	Mean change in systolic blood pressure at 12 months	N/A	BP measurements were obtained and averaged from each subject with use of the Omron HEM-907XL26 according to a standardized protocol provided by the manufacturer about cuff size, cuff application, body position, and time intervals when taking a measurement.				
								**UC (high BP)**	**79**	**74**	**UC**
								**Intervention (high B**	**89**	**84**	**Interventio**
								**UC (all records)**	**199**	**188**	**UC**
								**Intervention (all reco**	**199**	**186**	**Interventio**

For peer review only

Table S4. PRISMA checklist

Section and Topic	Item #	Checklist item	Location where item is reported
**TITLE**			
Title	1	Identify the report as a systematic review.	1
**ABSTRACT**			
Abstract	2	See the PRISMA 2020 for Abstracts checklist.	2
**INTRODUCTION**			
Rationale	3	Describe the rationale for the review in the context of existing knowledge.	3
Objectives	4	Provide an explicit statement of the objective(s) or question(s) the review addresses.	3
**METHODS**			
Eligibility criteria	5	Specify the inclusion and exclusion criteria for the review and how studies were grouped for the syntheses.	3
Information sources	6	Specify all databases, registers, websites, organisations, reference lists and other sources searched or consulted to identify studies. Specify the date when each source was last searched or consulted.	3
Search strategy	7	Present the full search strategies for all databases, registers and websites, including any filters and limits used.	Supplementary
Selection process	8	Specify the methods used to decide whether a study met the inclusion criteria of the review, including how many reviewers screened each record and each report retrieved, whether they worked independently, and if applicable, details of automation tools used in the process.	3
Data collection process	9	Specify the methods used to collect data from reports, including how many reviewers collected data from each report, whether they worked independently, any processes for obtaining or confirming data from study investigators, and if applicable, details of automation tools used in the process.	4
Data items	10a	List and define all outcomes for which data were sought. Specify whether all results that were compatible with each outcome domain in each study were sought (e.g. for all measures, time points, analyses), and if not, the methods used to decide which results to collect.	4
	10b	List and define all other variables for which data were sought (e.g. participant and intervention characteristics, funding sources). Describe any assumptions made about any missing or unclear information.	4
Study risk of bias assessment	11	Specify the methods used to assess risk of bias in the included studies, including details of the tool(s) used, how many reviewers assessed each study and whether they worked independently, and if applicable, details of automation tools used in the process.	4
Effect measures	12	Specify for each outcome the effect measure(s) (e.g. risk ratio, mean difference) used in the synthesis or presentation of results.	4
Synthesis methods	13a	Describe the processes used to decide which studies were eligible for each synthesis (e.g. tabulating the study intervention characteristics and comparing against the planned groups for each synthesis (item #5)).	4
	13b	Describe any methods required to prepare the data for presentation or synthesis, such as handling of missing summary statistics, or data conversions.	4
	13c	Describe any methods used to tabulate or visually display results of individual studies and syntheses.	4
	13d	Describe any methods used to synthesize results and provide a rationale for the choice(s). If meta-analysis was performed, describe the model(s), method(s) to identify the presence and extent of statistical heterogeneity, and software package(s) used.	4

For peer review only

Section and Topic	Item #	Checklist item	Location where item is reported
	13e	Describe any methods used to explore possible causes of heterogeneity among study results (e.g. subgroup analysis, meta-regression).	N/A
	13f	Describe any sensitivity analyses conducted to assess robustness of the synthesized results.	N/A
Reporting bias assessment	14	Describe any methods used to assess risk of bias due to missing results in a synthesis (arising from reporting biases).	N/A
Certainty assessment	15	Describe any methods used to assess certainty (or confidence) in the body of evidence for an outcome.	N/A
**RESULTS**			
Study selection	16a	Describe the results of the search and selection process, from the number of records identified in the search to the number of studies included in the review, ideally using a flow diagram.	4
	16b	Cite studies that might appear to meet the inclusion criteria, but which were excluded, and explain why they were excluded.	Supplementary
Study characteristics	17	Cite each included study and present its characteristics.	Table 1
Risk of bias in studies	18	Present assessments of risk of bias for each included study.	7 and Supplementary
Results of individual studies	19	For all outcomes, present, for each study: (a) summary statistics for each group (where appropriate) and (b) an effect estimate and its precision (e.g. confidence/credible interval), ideally using structured tables or plots.	8
Results of syntheses	20a	For each synthesis, briefly summarise the characteristics and risk of bias among contributing studies.	8
	20b	Present results of all statistical syntheses conducted. If meta-analysis was done, present for each the summary estimate and its precision (e.g. confidence/credible interval) and measures of statistical heterogeneity. If comparing groups, describe the direction of the effect.	8
	20c	Present results of all investigations of possible causes of heterogeneity among study results.	N/A
	20d	Present results of all sensitivity analyses conducted to assess the robustness of the synthesized results.	N/A
Reporting biases	21	Present assessments of risk of bias due to missing results (arising from reporting biases) for each synthesis assessed.	N/A
Certainty of evidence	22	Present assessments of certainty (or confidence) in the body of evidence for each outcome assessed.	N/A
**DISCUSSION**			
Discussion	23a	Provide a general interpretation of the results in the context of other evidence.	8
	23b	Discuss any limitations of the evidence included in the review.	8
	23c	Discuss any limitations of the review processes used.	9
	23d	Discuss implications of the results for practice, policy, and future research.	9
**OTHER INFORMATION**			

For peer review only

Section and Topic	Item #	Checklist item	Location where item is reported
Registration and protocol	24a	Provide registration information for the review, including register name and registration number, or state that the review was not registered.	3
	24b	Indicate where the review protocol can be accessed, or state that a protocol was not prepared.	3
	24c	Describe and explain any amendments to information provided at registration or in the protocol.	3
Support	25	Describe sources of financial or non-financial support for the review, and the role of the funders or sponsors in the review.	1, 4, 9
Competing interests	26	Declare any competing interests of review authors.	9
Availability of data, code and other materials	27	Report which of the following are publicly available and where they can be found: template data collection forms; data extracted from included studies; data used for all analyses; analytic code; any other materials used in the review.	N/A

For peer review only

Section and Topic	Item #	Checklist item	Location where item is reported
**TITLE**			
Title	1	Identify the report as a systematic review.	1
**ABSTRACT**			
Abstract	2	See the PRISMA 2020 for Abstracts checklist.	2
**INTRODUCTION**			
Rationale	3	Describe the rationale for the review in the context of existing knowledge.	3
Objectives	4	Provide an explicit statement of the objective(s) or question(s) the review addresses.	3
**METHODS**			
Eligibility criteria	5	Specify the inclusion and exclusion criteria for the review and how studies were grouped for the syntheses.	3
Information sources	6	Specify all databases, registers, websites, organisations, reference lists and other sources searched or consulted to identify studies. Specify the date when each source was last searched or consulted.	3
Search strategy	7	Present the full search strategies for all databases, registers and websites, including any filters and limits used.	Supplementary
Selection process	8	Specify the methods used to decide whether a study met the inclusion criteria of the review, including how many reviewers screened each record and each report retrieved, whether they worked independently, and if applicable, details of automation tools used in the process.	3
Data collection process	9	Specify the methods used to collect data from reports, including how many reviewers collected data from each report, whether they worked independently, any processes for obtaining or confirming data from study investigators, and if applicable, details of automation tools used in the process.	4
Data items	10a	List and define all outcomes for which data were sought. Specify whether all results that were compatible with each outcome domain in each study were sought (e.g. for all measures, time points, analyses), and if not, the methods used to decide which results to collect.	4
	10b	List and define all other variables for which data were sought (e.g. participant and intervention characteristics, funding sources). Describe any assumptions made about any missing or unclear information.	4
Study risk of bias assessment	11	Specify the methods used to assess risk of bias in the included studies, including details of the tool(s) used, how many reviewers assessed each study and whether they worked independently, and if applicable, details of automation tools used in the process.	4
Effect measures	12	Specify for each outcome the effect measure(s) (e.g. risk ratio, mean difference) used in the synthesis or presentation of results.	4
Synthesis methods	13a	Describe the processes used to decide which studies were eligible for each synthesis (e.g. tabulating the study intervention characteristics and comparing against the planned groups for each synthesis (item #5)).	4
	13b	Describe any methods required to prepare the data for presentation or synthesis, such as handling of missing summary statistics, or data conversions.	4
	13c	Describe any methods used to tabulate or visually display results of individual studies and syntheses.	4
	13d	Describe any methods used to synthesize results and provide a rationale for the choice(s). If meta-analysis was performed, describe the model(s), method(s) to identify the presence and extent of statistical heterogeneity, and software package(s) used.	4

For peer review only

Section and Topic	Item #	Checklist item	Location where item is reported
	13e	Describe any methods used to explore possible causes of heterogeneity among study results (e.g. subgroup analysis, meta-regression).	N/A
	13f	Describe any sensitivity analyses conducted to assess robustness of the synthesized results.	N/A
Reporting bias assessment	14	Describe any methods used to assess risk of bias due to missing results in a synthesis (arising from reporting biases).	N/A
Certainty assessment	15	Describe any methods used to assess certainty (or confidence) in the body of evidence for an outcome.	N/A
**RESULTS**			
Study selection	16a	Describe the results of the search and selection process, from the number of records identified in the search to the number of studies included in the review, ideally using a flow diagram.	4
	16b	Cite studies that might appear to meet the inclusion criteria, but which were excluded, and explain why they were excluded.	Supplementary
Study characteristics	17	Cite each included study and present its characteristics.	Table 1
Risk of bias in studies	18	Present assessments of risk of bias for each included study.	7 and Supplementary
Results of individual studies	19	For all outcomes, present, for each study: (a) summary statistics for each group (where appropriate) and (b) an effect estimate and its precision (e.g. confidence/credible interval), ideally using structured tables or plots.	8
Results of syntheses	20a	For each synthesis, briefly summarise the characteristics and risk of bias among contributing studies.	8
	20b	Present results of all statistical syntheses conducted. If meta-analysis was done, present for each the summary estimate and its precision (e.g. confidence/credible interval) and measures of statistical heterogeneity. If comparing groups, describe the direction of the effect.	8
	20c	Present results of all investigations of possible causes of heterogeneity among study results.	N/A
	20d	Present results of all sensitivity analyses conducted to assess the robustness of the synthesized results.	N/A
Reporting biases	21	Present assessments of risk of bias due to missing results (arising from reporting biases) for each synthesis assessed.	N/A
Certainty of evidence	22	Present assessments of certainty (or confidence) in the body of evidence for each outcome assessed.	N/A
**DISCUSSION**			
Discussion	23a	Provide a general interpretation of the results in the context of other evidence.	8
	23b	Discuss any limitations of the evidence included in the review.	8
	23c	Discuss any limitations of the review processes used.	9
	23d	Discuss implications of the results for practice, policy, and future research.	9
**OTHER INFORMATION**			

For peer review only

Section and Topic	Item #	Checklist item	Location where item is reported
Registration and protocol	24a	Provide registration information for the review, including register name and registration number, or state that the review was not registered.	3
	24b	Indicate where the review protocol can be accessed, or state that a protocol was not prepared.	3
	24c	Describe and explain any amendments to information provided at registration or in the protocol.	3
Support	25	Describe sources of financial or non-financial support for the review, and the role of the funders or sponsors in the review.	1, 4, 9
Competing interests	26	Declare any competing interests of review authors.	9
Availability of data, code and other materials	27	Report which of the following are publicly available and where they can be found: template data collection forms; data extracted from included studies; data used for all analyses; analytic code; any other materials used in the review.	N/A

For peer review only
